# The effects of higher versus lower protein delivery in critically ill patients: an updated systematic review and meta-analysis of randomized controlled trials with trial sequential analysis

**DOI:** 10.1186/s13054-023-04783-1

**Published:** 2024-01-06

**Authors:** Zheng-Yii Lee, Ellen Dresen, Charles Chin Han Lew, Julia Bels, Aileen Hill, M. Shahnaz Hasan, Lu Ke, Arthur van Zanten, Marcel C. G. van de Poll, Daren K. Heyland, Christian Stoppe

**Affiliations:** 1 Department of Anaesthesiology, Faculty of Medicine, University of Malaya, 50603 Kuala Lumpur, Malaysia; 2 Department of Cardiac Anesthesiology and Intensive Care Medicine, Charité, Berlin, Germany; 3 University Hospital Würzburg, Department of Anaesthesiology, Intensive Care, Emergency and Pain Medicine, Würzburg, Germany; 4 Department of Dietetics and Nutrition, Ng Teng Fong General Hospital, 1 Jurong East Street 21, Singapore, 609606 Singapore; 5 Department of Intensive Care Medicine, Maastricht University Medical Centre, Maastricht, 6229HX The Netherlands; 6 NUTRIM School for Nutrition and Translational Research in Metabolism, Maastricht University, Universiteitssingel 40, 6229 ER Maastricht, The Netherlands; 7 Department of Anesthesiology and Department Intensive Care Medicine, University Hospital RWTH Aachen, Pauwelsstraße 30, 52074 Aachen, Germany; 8 Department of Critical Care Medicine, Jinling Hospital, Medical School of Nanjing University, No. 305 Zhongshan East Road, Nanjing, 210000 Jiangsu Province China; 9 Department of Intensive Care Medicine, Gelderse Vallei Hospital, Ede & Wageningen University & Research, Wageningen, The Netherlands; 10 Clinical Evaluation Research Unit, Department of Critical Care Medicine, Queen’s University, Kingston, ON K7L 3N6 Canada

**Keywords:** Critical illness, Protein, Physical rehabilitation, Systematic review

## Abstract

**Background:**

A recent large multicentre trial found no difference in clinical outcomes but identified a possibility of increased mortality rates in patients with acute kidney injury (AKI) receiving higher protein. These alarming findings highlighted the urgent need to conduct an updated systematic review and meta-analysis to inform clinical practice.

**Methods:**

From personal files, citation searching, and three databases searched up to 29-5-2023, we included randomized controlled trials (RCTs) of adult critically ill patients that compared higher vs lower protein delivery with similar energy delivery between groups and reported clinical and/or patient-centred outcomes. We conducted random-effect meta-analyses and subsequently trial sequential analyses (TSA) to control for type-1 and type-2 errors. The main subgroup analysis investigated studies with and without combined early physical rehabilitation intervention. A subgroup analysis of AKI vs no/not known AKI was also conducted.

**Results:**

Twenty-three RCTs (*n* = 3303) with protein delivery of 1.49 ± 0.48 vs 0.92 ± 0.30 g/kg/d were included. Higher protein delivery was not associated with overall mortality (risk ratio [RR]: 0.99, 95% confidence interval [CI] 0.88–1.11; *I*^2^ = 0%; 21 studies; low certainty) and other clinical outcomes. In 2 small studies, higher protein combined with early physical rehabilitation showed a trend towards improved self-reported quality-of-life physical function measurements at day-90 (standardized mean difference 0.40, 95% CI − 0.04 to 0.84; *I*^2^ = 30%). In the AKI subgroup, higher protein delivery significantly increased mortality (RR 1.42, 95% CI 1.11–1.82; *I*^2^ = 0%; 3 studies; confirmed by TSA with high certainty, and the number needed to harm is 7). Higher protein delivery also significantly increased serum urea (mean difference 2.31 mmol/L, 95% CI 1.64–2.97; *I*^2^ = 0%; 7 studies).

**Conclusion:**

Higher, compared with lower protein delivery, does not appear to affect clinical outcomes in general critically ill patients but may increase mortality rates in patients with AKI. Further investigation of the combined early physical rehabilitation intervention in non-AKI patients is warranted.

**Prospero ID:**

CRD42023441059.

**Supplementary Information:**

The online version contains supplementary material available at 10.1186/s13054-023-04783-1.

## Background

The role of protein dosage in critically ill patients is of considerable interest as it is thought to improve clinical outcomes by attenuating protein losses during critical illness and supporting the patients’ recovery in later phases [1]. Consequently, clinical nutrition societies generally recommend higher protein delivery, whereas these recommendations are based on a low level of evidence, leading to varying dosage recommendations (ranging from 1.2 to 2.5 g/kg body weight [BW]/day) and uncertainties in the clinical practice [2–4] due to the unclear benefits and risks [5].

A previous systematic review and meta-analysis (SRMA) included 19 randomized controlled trials (RCTs) and 1731 patients comparing higher (~ 1.3 g/kg BW/day) vs. lower (~ 0.9 g/kg BW/day) protein delivery (with similar energy delivery between groups) found that higher protein delivery was not associated with overall mortality but significantly attenuated muscle loss in five small RCTs [6]. A trend towards shorter durations of mechanical ventilation (MV) and intensive care unit (ICU) length of stay (LOS) with higher protein delivery was also demonstrated [6]. Following this SRMA, several RCTs were published, and one of them represents the large multinational, multicentre EFFORT protein trial. This trial compared higher (1.6 g/ kg BW/day) vs. lower (0.9 g/kg BW/day) protein delivery and could not confirm any benefits or improved outcomes with higher protein delivery [7]. Rather, these results indicate that higher protein delivery may increase mortality censored at 60 days in patients with acute kidney injury (AKI) and high organ failure scores [7]. Although the EFFORT protein trial may itself already impact clinical practice, it is crucial to aggregate all available data to provide the best evidence to inform and guide clinical practice. Accordingly, the new relevant data from the EFFORT Protein trial and other recent RCTs need to be included in the updated SRMA to achieve greater precision on the pooled estimates*.* However, since the risks of type-I and -II errors may persist, trial sequential analysis (TSA) can be employed to detect such errors and thereby increase the certainty of the aggregated findings. Additionally, TSA quantifies the sample sizes required for clinically meaningful outcomes and offer insight into the potential futility of future trials, guiding feasibility, and choice of outcome measures. [8]

Currently, evaluation of biochemical and patient-centred outcomes is lacking in published SRMAs. The lack of these outcomes precludes a comprehensive understanding of the associated biochemical sequelae of higher protein delivery. Similarly, the pooled estimate of combining early physical rehabilitation and higher protein delivery on patient-centred outcomes is lacking. Since early physical rehabilitation may improve protein utilization, it is essential to quantify their synergistic effects.

In light of these considerations, there is an urgent need to update the previous SRMA to address the following objectives: (1) compare the effect of higher vs. lower protein delivery (with similar energy between groups) on clinical outcomes in critically ill patients with and without acute kidney injury (AKI) and early physical rehabilitation and (2) summarize the biochemical sequelae and physical function outcomes of higher protein delivery.

## Methodology

We conducted this SRMA according to the PRISMA 2020 guidelines [9]. The PRISMA 2020 checklist is shown in Additional file 1: supplementary methods. The study protocol was registered in PROSPERO (CRD42023441059).

### Eligibility criteria

We included RCTs of (1) adult (age ≥ 18) critically ill patients (mechanically ventilated or if uncertain, the control group mortality had to be greater than 5% to ensure including truly critically ill patients) that (2) compared protein doses with delivery via enteral (EN) formula, EN protein supplementation, parenteral nutrition (PN), or intravenous (IV) amino acids, (3) reported similar energy delivery between groups, and (4) reported clinical and/or patient-centred outcomes.

Studies among elective surgical or non-critically ill patients or studies with only laboratory, metabolic, or nutritional outcomes were excluded. Studies that investigated the effect of immunonutrition (e.g. glutamine or arginine) were also excluded. Quasi-randomized trials and studies published in abstract form were excluded. *Post hoc*, since our search also retrieved studies with a combination of protein and early physical rehabilitation, and the latter may enhance protein utilization, we also included studies with such combined interventions.

### Information source and search strategies

An updated systematic search in MEDLINE, EMBASE, and CENTRAL through OVID was conducted with relevant subject headings and keywords from our last search (1 April 2022) [6] to (29 May 2023) without language restrictions. Personal files and the reference list of previous SRMAs were reviewed. Additional file 1: Table S1 shows the search strategies. ClinicalTrials.gov was also searched for ongoing studies (Additional file 1: Table S2).

### Study selection process

Search results were exported into Covidence (Veritas Health Innovation, Melbourne, Australia) to remove duplicates and screen for potential eligible studies using the title and abstract of the articles (ZYL). The potential studies were retrieved, and two authors evaluated the full text independently (ZYL, ED). Disagreements were discussed with two other authors (CCHL and CS).

### Data collection process

Data items were collected independently by two authors (ZYL, ED) in a standardized data abstraction form and thereafter summarized into tables. Details of data handling are in Additional file 1: Supplementary Methods.

### Study quality and risk-of-bias assessment

The quality of the included trials was evaluated independently by two authors (ZYL, ED) using the Canadian Critical Care Nutrition (CCN) Methodological Quality System and the Cochrane Risk of Bias version 2 (ROB2). [10]. The overall ROB2 assessment was categorized as low risk of bias, some concerns, or high risk of bias. The risk-of-bias traffic light and summary plots were generated by the Risk-of-bias VISualization (robvis) tool [11]. The use of the CCN Methodological Quality System allows us to compare critical care nutrition trials across time and topics. The scoring table is shown in Additional file 1: Table S3. Any disagreements were discussed with two other authors (CCHL and CS).

### Outcomes

Overall mortality is the primary outcome; all other outcomes are secondary. These latter outcomes are: (i) nutritional outcomes, (ii) clinical outcomes, (iii) muscle outcomes, (iv) discharge to rehabilitation facilities, (v) quality of life (QOL) physical measurements, and (vi) biochemical outcomes (details of each outcome are in Additional file 1: Supplementary Methods). Outcomes with at least 2 studies were pooled and reported.

### Subgroup analysis

The following subgroup analyses were planned *a priori*: low vs other risk of bias, single vs multicentre trial, EN vs exclusive PN/intravenous amino acids, and AKI vs no/not known AKI. The subgroup analysis of AKI was performed in one study that enrolled exclusively AKI patients [12] and two studies that reported mortality outcomes in their subgroup of patients with AKI (Nephroprotect trial [12] and EFFORT protein trial [7]). For the Nephroprotect trial, we used the data from their secondary analysis that reported 90-day mortality outcome among patients with baseline kidney dysfunction (creatinine > 168 umol/L at the time of enrolment) and/or baseline risk of progression of AKI (creatinine increased over the previous 24 h by at least 20% to over 120 μmol/L) [13]. In both trials [7, 12], there were groups of patients with and without AKI. To ascertain the mortality count and total sample size for the no/not known AKI subgroup, the mortality count and total sample size of the AKI subgroup were subtracted from the overall mortality count and total sample size, respectively.

*Post hoc*, since we included studies combining higher protein and early physical rehabilitation, we added the subgroup analysis of studies with and without early physical rehabilitation. One study randomized patient to 3 groups (Group 1: usual care, Group 2: low protein + cycle ergometry, Group 3: high protein + cycle ergometry) [17], and we included groups 1 and 3 in our meta-analysis.

### Data analysis

Dichotomous outcomes were presented as risk ratio (RR), while continuous outcomes were presented as mean difference (MD) or standardized mean difference (SMD). For AKI subgroup analysis on mortality outcome, we performed an additional analysis to present the effect measure as risk difference (RD) in order to obtain the number needed to harm (1/RD). The DerSimonian–Laird random-effect model was used to account for the different patients’ characteristics, dosing, duration, and starting time of the protein delivery. Heterogeneity was quantified by the I^2^ measure. Publication bias was visualized by the funnel plot. Egger’s test was conducted for meta-analyses that included > 10 studies using STATA 16.1 (StataCorp LLC, Texas) [14]. All meta-analyses and tests for subgroup differences were conducted using RevMan 5.4 (Cochrane IMS, Oxford, UK). A two-sided *p* value of < 0.05 was considered statistically significant, and a *p* value of < 0.10 was considered a trend. [15]

### Trial sequential analysis

To control for type-I and type-II errors, TSA was performed using the TSA software (0.9.5.10 Beta, The Copenhagen Trial Unit, Denmark) with pre-specified parameters detailed in Additional file 1: Supplementary Methods.

### Certainty of the evidence

The Grading of Recommendations Assessment, Development, and Evaluation (GRADE) system was used to rate the certainty of evidence for outcomes analysed with TSA [16]. The quality of the evidence was rated as high, moderate, low, and very low by considering the risk of bias, inconsistency, indirectness, imprecision, and publication bias. The percentage of diversity-adjusted required information size (DARIS) achieved, and the TSA-adjusted 95% confidence interval for relative risk and mean difference were used to aid the assessment of imprecision in GRADE. GRADEpro was used to prepare the GRADE evidence profile table.

## Results

### Study selection

Our search identified an additional 853 articles (391 from MEDLINE, 350 from EMBASE, and 113 from CENTRAL). After removing duplicates and article screening and review, we included 23 RCTs (an additional 4 RCTs [7, 17–19] from our previous SRMA). The detailed study selection flow is presented in Additional file 1: Fig. S1. The list of excluded studies and reasons for exclusion are presented in Additional file 1: Table S4. Our search on ClinicalTrials.gov and personal files identified 13 ongoing or unpublished related trials (Additional file 1: Table S2).

### Studies and patients’ characteristics

Twenty-three RCTs with 3,303 patients were included. The study characteristics are summarized in Table 1. Sample sizes ranged from 20 to 1,301. Patients’ baseline characteristics and the detailed nutritional data are summarized in Additional File 1: Tables S5 and S6.

**Table 1:** Study population, nutrition route, and timing of intervention

Author, year (country)	N*	Population (number of centre)	EN	PN	Start intervention		Days on intervention	
					High	Low	High	Low
1. Clifton 1985 [29] (USA)	20	Severe head injury (1)	EN only	–	Balance period: ~ 7–14d after injury	Balance period: ~ 7–14d after injury	~ 7d	~ 7d
2. Saffle 1990 [19] (USA)	49	Acute burns with % total burn surface area ≥ 25% (1)	EN only	–	After fluid resuscitation and placement of feeding tube	After fluid resuscitation and placement of feeding tube	~ 22d	~ 22d
3. Mesejo 2003 [37] (Spain)	50	EN ≥ 5 d, APACHE II 10–25, BMI ≥ 30, no kidney/liver failure (2)	EN only	–	~ ≤ 48 h of ICU admission	~ ≤ 48 h of ICU admission	5d	5d
4. Zhou 2006 [30] (China)	51	Severe stroke with GCS < 12 (1)	EN only	–	≤ 5d of acute stroke	≤ 5 days of acute stroke	– (up to 14d)	– (up to 14d)
5. Singer 2007 [12] (Israel)	14	MV with non-oliguric acute renal failure and required PN (1)	–	PN only	D2 of ICU admission	D2 of ICU admission	3d	3d
6. Rugeles 2013 [33] (Columbia)	80	Medical, EN ≥ 96 h (1)	EN only	Exclude patients that need PN	~ ≤ 48 h of ICU admission	~ ≤ 48 h of ICU admission	≥ 96 h (up to 7d)	≥ 96 h (up to 7d)
7. Doig 2015 [20] (Australia)	474	Mixed, Stay ≥ 2d (16)	Decide by the attending physician		D1-2 of ICU admission	D1-2 of ICU admission	At D7, *n* = 124 (Until ICU DC: ICU LOS 11.6d)	At D7, *n* = 120 (Until ICU DC: ICU LOS 10.7d)
8. Ferrie 2015 [21] (Australia)	120	Mixed, ≥ 3d on PN (1)	–	PN only	1 (1–2) d in ICU	1 (1–2) d in ICU	10.0 (6.8–14.0) d (up to 10d)	9.5 (7.0–13.5) d (up to 10d)
9. Jakob 2017 [22] (Switzerland)	90	Mixed, EN ≥ 3d, stay ≥ 5d (1)	EN first	PN is only allowed if intolerant to EN	Time to reach full caloric goal: 2.2 (0.8–3.7)d	Time to reach full caloric goal: 2.0 (1.3–2.7)d	5.0 (3.6–6.4) d (up to 10d)	7.0 (5.3–8.7) d (up to 10d)
10. Fetterplace 2018 [23] (Australia)	60	Mixed, MV within 48 h and remained ≥ 72 h (1)	EN first	PN is allowed at the discretion of the treating physician	Time EN start: 13 ± 8 h	Time EN start: 20 ± 10 h	At D7, *n* = 15 (up to 15d)	At D7, *n* = 12 (up to 15d)
11. van Zanten 2018 [24] (Netherlands)	44	Mixed, MV, BMI ≥ 25, EN ≤ 48 h- > 5d (4)	EN first	SPN is allowed if necessary	D1-2 of ICU admission	D1-2 of ICU admission	At D10, *n* = 16 (up to 28d)	At D10, *n* = 13 (up to 28d)
12. Vega-Alava 2018 [36] (Philippines)	40	MV, EN (1)	EN only	–	~ ≤ 24 h of ICU admission	~ ≤ 24 h of ICU admission	–	–
13. Azevedo 2019 [25] (Brazil)	120	Mixed, MV, Stay > 2d (1)	EN first	SPN is allowed after 5 d if the caloric goal is not achieved	~ ≤ 3d in the ICU (IC to adjust caloric intake)	~ ≤ 3d in the ICU (IC to adjust caloric intake)	– (up to 14d)	– (up to 14d)
14. Danielis 2019 [26] (Italy)	40	Mixed, MV within 12 h, BMI 18.5 to 30 kg/m [2], no acute/chronic renal or hepatic failure (1)	EN first	SPN is allowed to make up the energy shortfall	Once admitted to the ICU and assessed for eligibility	Once admitted to the ICU and assessed for eligibility	– (up to the end of MV, onset of acute renal or hepatic failure, transfer to another hospital or death)	
15. Bukhari 2020^ [35] (Indonesia)	33	ICU patients not contraindicated or intolerant to EN (1)	EN only	–	Within 24–48 h of ICU admission	Within 24–48 h of ICU admission	3 d	3 d
16. Chapple 2020 [27] (Australia)	116	Mixed, MV, EN > 2d (6)	EN first	SPN is allowed if deemed necessary by the treating physician	~ 19 h of ICU admission	~ 17.6 h of ICU admission	8.7 ± 7.3d (up to 28d)	8.1 ± 6.3d (up to 28d)
17. Nakamura 2020 [28] (Japan)	117	Mixed, no lower limb injury, no die or discharge < D10 (1)	EN first	SPN is allowed to reach energy goal within 3d (no IV AA)	Time EN start: < 48 h ICU admission	Time EN start: < 48 h ICU admission	EN: 8 (5–9) d. Oral up to D10	EN: 8 (5–9) d. Oral up to D10
18. Carteron 2021 [31] (France)	195	Brain injured (GCS < 8), expected MV > 48 h (1)	EN only	–	Within 36 h of ICU admission	Within 36 h of ICU admission	At D10, *n* = 52 (up to 10d)	At D10, *n* = 60 (up to 10d)
19. Dresen 2021 (Germany) [34]	42	Surgical, MV, after stay ≥ 10d, expected stay ≥ 30d (1)	EN first	If the nutrition target was not achieved within 24 h, initiate SPN	After ≥ 10 d in the ICU	After ≥ 10 d in the ICU	At D25, *n* = 15 (up to 28d)	At D25, *n* = 12 (up to 28d)
20. Heyland 2023# [7] (International)	1301	Mixed, MV within 96 h of ICU admission and remained ≥ 48 h with a ‘high’ nutrition risk (85)	EN first	TPN or SPN is allowed if deemed necessary by the treating physician	Within 96 h of MV	Within 96 h of MV	Up to 28 d in the ICU	Up to 28 d in the ICU
*Studies with combined high protein and early physical rehabilitation*								
21. Badjatia 2020 [32] (USA)	25	SAH, Stay > 7d, BMI 15 to 40 (1)	EN or oral intake	–	Time EN start: < 24 h of aneurysmal repair	Time EN start: < 24 h of aneurysmal repair	12 d (range 9–14)	12 d (range 9–14)
22. Azevedo 2021 [18] (Brazil)	181	Mixed, MV, stay > 3 d (1)	EN first	SPN if protein goal was not reached by day 7–10	Third day of randomization	Third day of randomization	Up to 14 d	Up to 14 d
23. Kagan 2022^ [17] (Israel)	41	MV for ≥ 48 h and expected MV for a minimum of 7 d (1)	EN only	Exclude patients that need TPN	After 48 h of MV	After 48 h of MV	Up to 28 d	Up to 28 d

AA: amino acid, APACHE II: Acute Physiology and Chronic Health Evaluation II, BMI: body mass index, d: day(s), DC: discharge, EN: enteral nutrition, GCS: Glasgow Coma Scale, h: hour, IC: indirect calorimetry, ICU: intensive care unit, IV: intravenous, LOS: length of stay, Mixed: medical and surgical populations, MV: mechanical ventilation, PN: parenteral nutrition, SPN: supplemental parenteral nutrition

*N: number of patients analysed (total number of patients in 23 included studies = 3303)

^ Bukhari 2020 has 3 groups: control (*n* = 22), high-protein polymeric (*n* = 19) and oligomeric group (*n* = 14), the control group was excluded from the analysis

^ Kagan 2022 has 3 groups: control is group 1 (*n* = 22), group 2 received usual protein dose and cycle ergometry (*n* = 21), group 3 received high protein dose and cycle ergometry (*n* = 19); group 2 was excluded from the analysis

^Zhou 2022 has 2 groups: control (*n* = 50), early mobilization group (*n* = 50) and early mobilization combined with early nutrition group (*n* = 50)

^#^’High’ nutrition risk is defined by one of the following risk factors: BMI ≤ 25 or ≥ 35 kg/m^2^, moderate to severe malnutrition as defined by local assessments, Clinical Frailty Scale ≥ 5, SARC-F score ≥ 4, projected duration of MV of more than 4 days from the point of screening

The study population included mixed medical and surgical population (11 studies [7, 18, 20–28]), patients with stroke or head injury (4 studies [29–32]), only medical patients (1 study [33]), only surgical patients (1 study [34]), patients with non-oliguric acute renal failure (1 study [12]), patients with burn (1 study [19]), and unclear population (4 studies [17, 35–37]). Outcomes of patients with AKI are available in 3 studies [7, 12, 20], of which 1 is reported in a separate publication [13].

Twenty studies primarily used enteral nutrition (EN), and three used exclusive parenteral nutrition (PN) [12, 20, 21] strategy to increase protein delivery. Of the 20 studies that used an EN strategy, supplemental PN was allowed in 10 studies. [7, 18, 20, 22–25, 27, 28, 34]

Nineteen studies started the intervention within 3 days of ICU admission [12, 17–28, 31–33, 35–37]. The remaining studies started the intervention within 96 h of mechanical ventilation [7], 5 days of acute stroke [30], 7–14 days after a head injury [29], and after 10 days in the ICU [34]. The duration of intervention ranged from 3 to 28 days.

### Protein and energy delivery

Of the 23 included studies, 9 and 10 studies did not report the protein and energy delivered in g/kg BW/d or kcal/kg BW/d, respectively. The pooled mean protein delivery for the higher vs lower protein group was 1.49 ± 0.48 vs 0.92 ± 0.30 g/kg BW/d (14 studies, *n* = 2439), respectively, resulting in a daily MD of 0.49 g/kg BW/d (95% confidence interval [CI] 0.37–0.61, *p* < 0.00001; *I*^2^ = 94%) more protein delivery in the higher protein group. In contrast, the pooled mean energy delivery for the higher vs lower protein group was 17.48 ± 6.85 vs 16.60 ± 6.63 kcal/kg BW/d (13 studies, *n* = 2258), with no difference in daily energy delivery between groups (MD 0.13 kcal/kg BW/d, 95% CI − 1.25 to 1.52, *p* = 0.85; *I*^2^ = 91%) (Additional file 1: Fig S2).

### Early physical rehabilitation delivery

Two studies combined high protein and early physical rehabilitation [17, 18], and one study combined high protein and neuromuscular electrical muscle stimulation (NMES) [32], which are collectively named as combined early physical rehabilitation intervention. The details of the intervention are summarized in Additional file 1: Table S7. The NMES intervention was delivered in two 30-min sessions per day for up to 14 days. [32] For cycle ergometry, one study started immediately after randomization and delivered the intervention in two 15-min sessions/day for up to 21 days. [18] Another study started cycle ergometry within 24 h of randomization and delivered the intervention for up to 28 days, either passive cycling for 20 min/day or two 10-min sessions/day if a patient was able to cycle actively. [17]

### Study quality assessments

The median CCN methodological quality score of included studies was 8 (out of 14 [higher score indicates higher quality]). A total of 10 studies had a methodological quality score of > 8 [7, 17, 21–24, 27, 29, 32, 36] (Additional file 1: Table S8). The ROB2 plots are presented in Additional file 1: Figure S3. In 21 studies that reported mortality outcomes, 4/21 (19%) studies were at low risk of bias, 14/21 (67%) had some concerns, and 3/21 (14.3%) were at high risk of bias. The biases mainly arose from the randomization process and selection of the reported results.

### Results of the clinical outcomes

All outcomes are summarized in Additional file 1: Table S9 and Table S10.

#### Mortality

**Fig. 1:**
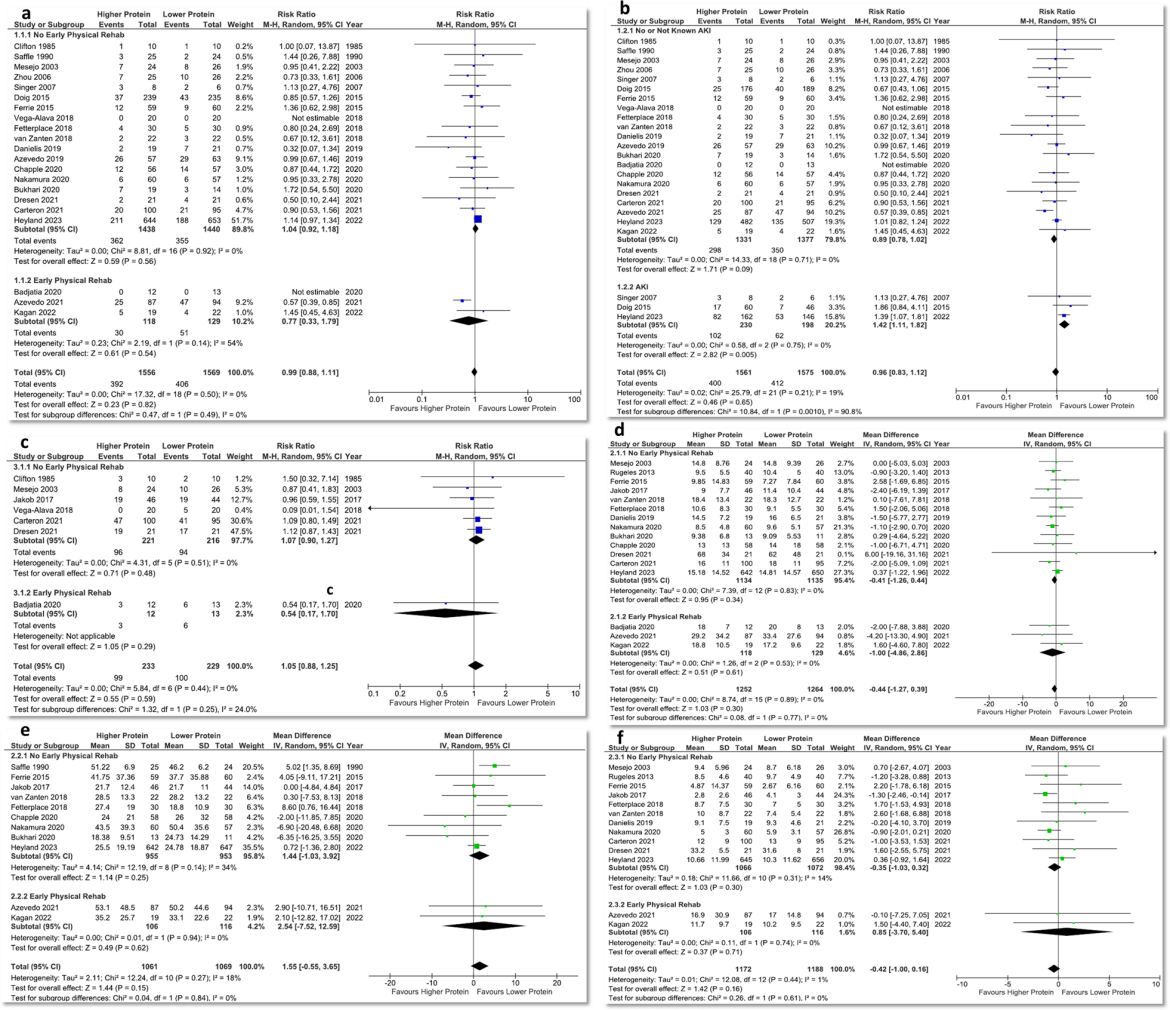
Meta-analysis of clinical outcomes. **a** Overall mortality (all patients), **b** Overall mortality (subgroup analysis of no/not known AKI vs AKI)*, **c** infectious complications (no change from previous meta-analysis), **d** ICU length of stay, **e** hospital length of stay, **f** duration of mechanical ventilation. AKI: acute kidney injury. *Note: **b**, **c**: AKI subgroup: mortality from Doig 2015 is 90-d mortality from their secondary publication [13]. Definitions: Singer 2007: *AKI*—50% decrease in GFR, a doubling of serum creatinine or an increase of creatinine to 3.5 mg/dL (309.4 umol/L); Doig 2015 (mortality of patients with kidney dysfunction or risk of progression of AKI from Doig 2015 is 90-d mortality from their secondary publication [13]): *Baseline kidney dysfunction*—creatinine at time of enrolment > 168 μmol/L (by Gordon Bernard’s “Brussels Table”), *Risk of progression of AKI at enrolment*—a rise in creatinine over the previous 24 h by at least 20% to over 120 μmol/L; Heyland 2023: *AKI*—patients who met the criteria of KDIGO: stage 1 is at least 26·52 μmol/L increase in serum creatinine from baseline within 48 h or 1·5–1·9 times baseline within 7 days, stage 2 is 2·0–2·9 times baseline within 7 days, or stage 3 is three times or more baseline within 7 days or increase to at least 353·6 μmol/L with an acute increase of more than 44·2 μmol/L. c: To ascertain the mortality count and total sample size for the no/not known AKI subgroup for Doig 2015 and Heyland 2023, the mortality count and total sample size of the AKI subgroup were subtracted from the overall mortality count and total sample size, respectively; mortality for Doig 2015 is 90-day mortality

A total of 21 studies reported mortality outcomes (*n* = 3125), and 3 of them included combined early physical rehabilitation intervention. No difference was found between higher and lower protein groups (RR 0.99, 95% CI 0.88–1.11, *p* = 0.82; *I*^2^ = 0%) in the overall analysis or between the subgroups with vs without early physical rehabilitation (test for subgroup differences *p* = 0.49) (Fig. 1a). No evidence of funnel plot asymmetry was detected (Additional file 1: Fig. S4a). Similarly, no differences were found between groups for ICU mortality, hospital mortality, 28-d mortality, and ≥ 60-d mortality (Additional file 1: Fig. S5). The combination of higher protein and early physical rehabilitation resulted in significantly lowered ≥ 60-d mortality (RR 0.61, 95% CI 0.43–0.87; 1 study [18], while no differences in ≥ 60-d mortality were found with higher protein intervention alone (RR 1.05, 95% CI 0.92–1.19; 8 studies); test for subgroup differences *p* = 0.005) (Additional file 1: Fig. S5d).

Subgroup analysis of no/not known AKI versus AKI found that higher protein delivery significantly increased mortality in AKI subgroup (RR 1.42, 95% CI 1.11–1.82, *p* = 0.005; *I*^2^ = 0%; 3 studies). The absolute pooled risk difference was 14% (Additional file 1: Fig. S5e), and number needed to harm was 7. There was a trend towards reduced mortality in no/not known AKI subgroup (RR 0.89, 95% CI 0.78, 1.02; *p* = 0.09; *I*^2^ = 0%; 21 studies). The test for subgroup differences was significant (*p* = 0.001) (Fig. 1b).

#### Infectious complications, ICU, and hospital length of stay and duration of mechanical ventilation

No significant differences were found between groups for infectious complications (RR 1.05, 95% CI 0.88–1.25, *p* = 0.59, *I*^2^ = 0%; 7 studies), ICU LOS (MD − 0.44, 95% CI − 1.27 to 0.39, *p* = 0.30; *I*^2^ = 0%; 16 studies), hospital LOS (MD 1.55, 95% CI − 0.55 to 3.65, *p* = 0.15; *I*^2^ = 18%; 11 studies), and duration of MV (MD − 0.42, 95% CI − 1.00 to 0.16, *p* = 0.16; *I*^2^ = 1%; 13 studies). All the tests for subgroup differences between studies with and without early physical rehabilitation were not different (Fig. 1c–f). No evidence of funnel plot asymmetry was detected except for the duration of mechanical ventilation (Additional file 1: Fig. S4b–4e).

### Results of muscle mass and strength, discharge to rehabilitation facilities, self-reported quality of life physical function outcomes, and incidence of diarrhoea

**Fig. 2:**
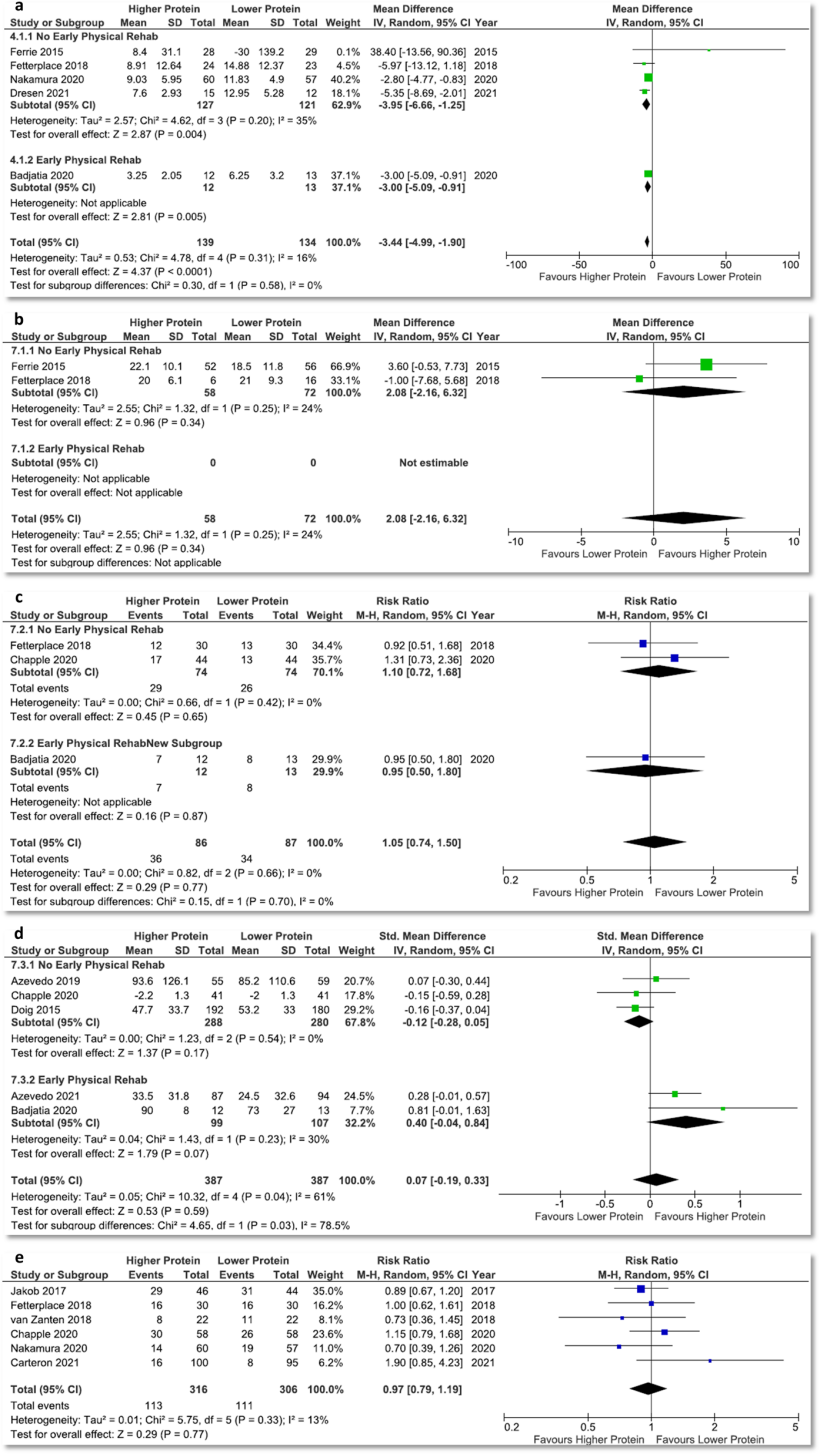
Meta-analysis of other outcomes. **a** Percentage of muscle change per week (no changes from previous meta-analysis), **b** handgrip strength, **c** discharge to rehabilitation facilities (no changes from previous meta-analysis), **d** self-reported quality of life physical function at day 90, **e** incidence of diarrhoea (no changes from previous meta-analysis). Note: **b** Fetterplace 2018: the best handgrip strength at awakening, ICU discharge, or day 15, Ferrie 2015: handgrip strength at day 7. Unable to analyse handgrip strength from Azevedo 2019 because unknown sample size for male and female. **d** The quality of life (QOL) outcomes reported by the studies were: Doig 2015: RAND-36 general health and physical function at day 90; Azevedo 2019: SF-36 physical component summary (PCS) score at 3 and 6 month; Badjatia 2010: fatigue, lower extremity mobility, and cognition outcomes based on the Neuro-QoL questionnaires administered on post-bleed day 90; Chapple 2020: EQ-5D-5L score for mobility, self-care, usual activities, pain/discomfort, anxiety/depression, and the result of the EQ-5D-5L visual analogue scale, all at day 90, Azevedo 2021: SF-36 physical component score at day 3 and 6 month (see Additional file 1: Table S9). The meta-analysis was performed for QOL results associated with physical function: RAND-36 physical function at day 90 (Doig 2015), SF-36 PCS score at 3 month (Azevedo 2019), Neuro-QoL lower extremity mobility on post-bleed day 90 (Badjatia 2010), EQ-5D-5L score for mobility at day 90 (Chapple 2020), and SF-36 physical component score at 3 month (Azevedo 2021). Higher EQ-5D-5L mobility score means worse performance; a negative is added to the mean score to reverse the direction of the results

No new studies were added to the meta-analysis on change in muscle mass, discharge location, and incidence of diarrhoea (Fig. 2a, 2c and 2e); therefore, findings are identical to our previous published meta-analysis [6]. Notably, higher protein delivery is associated with a muscle loss attenuation (MD − 3.44% per week, 95% CI − 4.99 to − 1.90, *p* < 0.0001, *I*^2^ = 16%; 5 studies; Fig. 2a).

No differences in muscle strength (Fig. 2b) and self-reported quality of life physical function (Fig. 2d) measurements were detected. However, in the subgroup of studies with combined early physical rehabilitation intervention, a trend towards improvement in physical function measures (SMD 0.40, 95% CI − 0.04 to 0.84, *p* = 0.07, *I*^2^ = 30%; 2 studies; Fig. 2d) was demonstrated, while no significant improvement was shown in studies without the combined intervention (SMD − 0.12, 95% CI − 0.28 to 0.05, *p* = 0.17; *I*^2^ = 0%; 3 studies). The test for subgroup differences was significant (*p* = 0.03).

### Results of biochemical outcomes

The biochemical outcomes between groups are summarized in Additional file 1: Table S10. Meta-analyses demonstrated that higher protein delivery significantly increased serum urea (MD 2.31 mmol/L, 95% CI 1.64–2.97, *p* < 0.00001, *I*^2^ = 0%; 7 studies), urinary urea nitrogen (MD 5.55 g, 95% CI 0.87–10.23, *p* = 0.02, *I*^2^ = 81%; 3 studies), and lymphocyte count (MD 257.43 cells per µL of blood, 95% CI 139.85–375.02, *p* < 0.0001, *I*^2^ = 0%; 4 studies). Higher protein delivery showed a trend towards a significant increase in prealbumin level (MD 1.96 mg/dL, 95% CI 0.00–3.91, *p* = 0.05; *I*^2^ = 23%; 4 studies) and nitrogen balance (MD 2.76 g, 95% CI − 0.38 to 5.90, *p* = 0.08; *I*^2^ = 78%; 5 studies). No significant differences between groups were found for serum creatinine, blood glucose, insulin administration, albumin, haemoglobin, total white blood cells, C-reactive protein, interleukin-6, phosphate, and triglyceride level (Additional file 1: Fig. S6a–S6p).

### Other subgroup analyses

No subgroup differences were detected between studies with low risk of bias and other risk of bias (Additional file 1: Fig. S7a–S7j) and studies that primarily used EN versus exclusive PN to increase protein delivery (data not shown). No subgroup differences were detected between single and multicentre studies (Additional file 1: Fig. S8a–8j).

### Trial sequential analysis

**Fig. 3:**
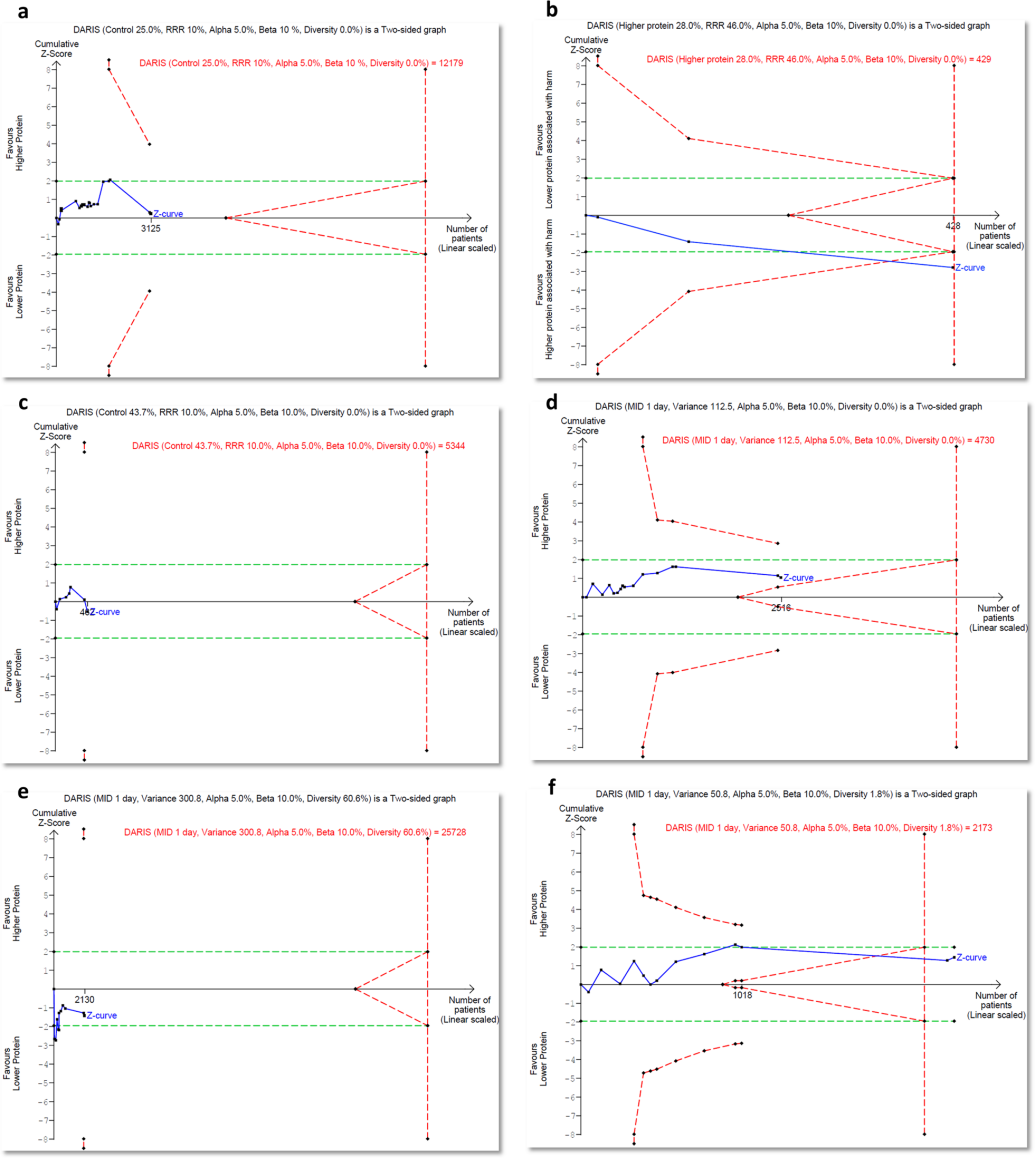
Trial Sequential Analysis of Clinical Outcomes. **a** Overall mortality in all patients (21 studies, *n* = 3125), **b** overall mortality in patients with acute kidney injury before protein intervention (3 studies, *n* = 428), **c** infectious complications (7 studies, *n* = 642), **d** intensive care unit length of stay (16 studies, *n* = 2516), **e** hospital length of stay (11 studies, *n* = 2130), **f** duration of mechanical ventilation (13 studies, *n* = 2360). TSA was analysed using DerSimonian and Laird random-effects model. The Z curve in blue measures the treatment effect (pooled relative risk). The parallel lines in green are the boundaries of conventional meta-analysis (alpha 5%), and the boundaries of benefit and harm are boundaries of conventional meta-analysis adjusted for between-trial heterogeneity and multiple statistical testing (TSA boundaries). A treatment effect outside the TSA boundaries of benefit/harm indicates reliable evidence for a treatment effect, and a treatment effect within the futility zone (the triangle between the parallel lines) indicates that there is reliable evidence of no treatment effect. DARIS: diversity adjusted required information size is the calculated optimum sample size for statistical inference, MID: minimally important difference, RRR: relative risk reduction, TSA: trial sequential analysis

Results of TSA are summarized in Table 2 and presented in Fig. 3 and Additional file 1: Figure S9, showing that the current systematic review did not achieve the required information sizes to detect the pre-specified effect sizes for overall mortality, infectious complications, ICU and hospital length of stay, change in muscle mass, handgrip strength, incidence of diarrhoea, and discharge to rehabilitation facilities, indicating that more trials are required for a definitive conclusion for these outcomes.

**Table 2:** Summary of results of trial sequential analyses

Effect size	Incidence, or variance	I^2^ (%)	D^2^ (%)	RIS	% of RIS attained	Z-curve passed the conventional boundaries?	Z-curve passed the TSA boundaries?	Z-curve passed the futility boundaries?
*Overall mortality (21 studies, n = 3125)*								
RRR: 10.0%	25.0%	0.0	0.0	12,179	25.7	No	No	No
*Overall mortality in patients with acute kidney injury before protein intervention (3 studies, n = 428)*								
RRR: 46.0%	28.0%	0.0	0.0	429	99.8	Yes	Yes	No
*Infectious complication (7 studies, n = 462)*								
RRR 10.0%	43.7%	0.0	0.0	5344	8.6	No	No	No
*Intensive care unit length of stay (16 studies, n = 2516)*								
MID 1 day	112.5	0.0	0.0	4730	53.2	No	No	Trending
*Hospital length of stay (11 studies, n = 2130)*								
MID 1 day	327.9	18.0	46.4	25,728	8.3	No	No	No
*Mechanical ventilation duration (13 studies, n = 2360)*								
MID 1 day	50.8	1.0	1.8	2173	108.6	No	No	Yes
*Incidence of diarrhoea (6 studies, n = 622)*								
RRR 10.0%	36.3%	13.0	19.2	8915	7.0	No	No	No
*Muscle wasting per week (5 studies, n = 273)*								
MID 1%	29.8	16.0	29.6	1780	15.3	Yes	No	No
*Handgrip strength (2 studies, n = 130)*								
MID 5 kg	104.5	24.0	31.4	256	50.8	No	No	Trending
*Discharge to rehab (3 studies, n = 173)*								
RRR 10.0%	39.1%	0.0	0.0	UTE	UTE	No	No	No

D^2^: diversity, I^2^: inconsistency, MID: minimally important difference, RIS: required information size, RD: risk difference, RRR: relative risk reduction

In patients with AKI, TSA confirmed the increase in mortality with high certainty. TSA revealed that further trials would be futile to detect a one-day difference in the duration of mechanical ventilation.

### GRADE certainty assessments

Higher protein delivery did not affect overall mortality in critically ill patients (low certainty of evidence). On the contrary, higher protein delivery increased mortality among patients with AKI (high certainty of evidence). The certainty of evidence of the effect of higher protein on other outcomes is low to very low (Table 3).

**Table 3:** GRADE certainty assessment and summary of findings table

Certainty assessment							Summary of findings				
Participants (studies) Follow-up	Risk of bias	Inconsistency	Indirectness	Imprecision	Publication bias	Overall certainty of evidence	Study event rates (%)		Relative effect (95% CI)	Anticipated absolute effects	
							With placebo	With mortality		Risk with placebo	Risk difference with mortality
*Overall mortality*											
3125 (21 RCTs)	serious^a^	not serious	not serious	serious^b^	none	⨁⨁◯◯ Low	406/1569 (25.9%)	392/1556 (25.2%)	RR 0.99 (0.88 to 1.11)	259 per 1,000	3 fewer per 1,000 (from 31 fewer to 28 more)
*Mortality (subgroup of patients with acute kidney injury)*											
428 (3 RCTs)	not serious^c^	not serious	not serious	not serious^d^	none	⨁⨁⨁⨁ High	62/198 (31.3%)	102/230 (44.3%)	RR 1.42 (1.11 to 1.82)	313 per 1,000	132 more per 1,000 (from 34 to 257 more)
*Infectious complications*											
462 (7 RCTs)	serious^e^	not serious	not serious	very serious^f^	none	⨁◯◯◯ Very low	100/229 (43.7%)	99/233 (42.5%)	RR 1.05 (0.88 to 1.25)	437 per 1,000	22 more per 1,000 (from 52 fewer to 109 more)
*ICU length of stay*											
2516 (16 RCTs)	serious^g^	not serious	not serious	serious^h^	none	⨁⨁◯◯ Low	1264	1252	–	The mean ICU length of stay was 0	MD 0.44 lower (1.27 lower to 0.39 higher)
*Hospital length of stay*											
2130 (11 RCTs)	serious^i^	serious^j^	not serious	very serious^k^	none	⨁◯◯◯ Very low	1069	1061	–	The mean hospital length of stay was 0	MD 1.55 higher (0.55 lower to 3.65 higher)
*Duration of mechanical ventilation*											
2360 (13 RCTs)	serious^l^	not serious	not serious	not serious	Publication bias strongly suspected	⨁⨁◯◯ Low	1188	1172	–	The mean duration of mechanical ventilation was 0	MD 0.42 lower (1 lower to 0.16 higher)
*Incidence of diarrhoea*											
622 (6 RCTs)	serious^e^	not serious	not serious	very serious^m^	none	⨁◯◯◯ Very low	111/306 (36.3%)	113/316 (35.8%)	RR 0.97 (0.79 to 1.19)	363 per 1,000	11 fewer per 1,000 (from 76 fewer to 69 more)
*Attenuation of thigh muscle loss (per week)*											
273 (5 RCTs)	serious^n^	not serious	not serious	serious^o^	none	⨁⨁◯◯ Low	pdate134	139	–	The mean attenuation of thigh muscle loss (per week) was 0	MD 3.44 lower (4.99 lower to 1.9 lower)
*Handgrip Strength*											
130 (2 RCTs)	very serious^p^	not serious	not serious	serious^q^	none	⨁◯◯◯ Very low	72	58	–	The mean handgrip strength was 0	MD 2.08 higher (2.16 lower to 6.32 higher)
*Discharge to rehabilitation facilities*											
173 (3 RCTs)	not serious	not serious	not serious	very serious^r^	none	⨁⨁◯◯ Low	34/87 (39.1%)	36/86 (41.9%)	RR 1.05 (0.74 to 1.50)	391 per 1,000	20 more per 1,000 (from 102 fewer to 195 more)

CI: confidence interval; MD: mean difference; RR: risk ratio

^a^4/21 (19%) studies were at low risk of bias, 14/21 (67%) had some concerns and 3/21 (14.3%) were at high risk of bias

^b^Trial sequential analysis revealed that only 25.7% of the required information sizes were attained to detect a relative risk reduction of 10%

^c^The largest study had low risk of bias, the moderate study had some concerns, the smallest study had high risk of bias. Overall, the results are consistent and therefore we rated this as not serious

^d^Trial sequential analysis confirmed the increase in mortality with high certainty

^e^All studies had some concerns

^f^Trial sequential analysis revealed that only 8.6% of the required information sizes were attained to detect a relative risk reduction of 10%

^g^Only 4/16 (25%) of the studies are low risk of bias

^h^Trial sequential analysis revealed that only 53.2% of the required information sizes were attained to detect a 1-day difference in ICU length of stay

^i^Only 3/11 (27.3%) of the studies are low risk of bias

^j^Diversity from TSA is 46.4%

^k^Trial sequential analysis revealed that only 8.3% of the required information sizes were attained to detect a one day a 1-day difference hospital length of stay

^l^Only 3/13 (23.1%) of the studies are low risk of bias

^m^Trial sequential analysis revealed that only 7% of the required information sizes were attained to detect a relative risk reduction of 10%

^n^Only 1/5 (20%) of the studies are low risk of bias

^o^Trial sequential analysis revealed that only 15.3% of the required information sizes were attained to detect a 1% attenuation of muscle loss per week

^p^Both included studies are at high risk of bias

^q^Trial sequential analysis revealed that only 50.8% of the required information sizes were attained to detect a 5-kg difference in handgrip strength

^r^Sample size is too small for trial sequential analysis to determine the required information size to detect a relative risk reduction of 10%

^s^Funnel plot asymmetry, Egger’s test *p* = 0.0308 (Additional file 1: Figure S9c)

## Discussion

This updated SRMA with overall 23 RCTs (3303 patients) of higher versus lower protein delivery, mostly commenced within 3 days of ICU admission, and with similar energy delivery between groups, highlighted that higher protein delivery was not associated with improvements in clinical outcomes (overall mortality, infectious complications, ICU, and hospital length of stays) as well as muscle strength, discharge location, and incidence of diarrhoea; however, TSA indicated that more trials are needed to further confirm these findings. Importantly, higher protein delivery was associated with increased mortality among patients with AKI, a result confirmed by TSA. A non-statistically significant trend towards reduced mortality was found in subgroup of patients with no/not known AKI, and further trials in non-AKI are warranted to confirm this finding.

This SRMA also found that higher protein delivery may attenuate muscle loss by about 3.4% per week; however, this finding was reported in a small number of studies, and TSA demonstrated a type-1 error, indicating that more studies are needed to improve the certainty of this finding. Furthermore, the combination of high protein delivery and early physical rehabilitation may improve self-reported quality of life physical function measures at day 90 after ICU admission (2 studies). Higher protein also significantly increased serum urea, urinary urea nitrogen, and lymphocyte count.

### Interpretation of the results in the context of other evidence

Our findings suggest that higher protein delivery may harm patients with AKI. Despite the heterogeneous definition of AKI in the three meta-analysed studies, the direction of the results is similar (*I*^2^ = 0%), particularly from the two included multicentre RCTs [7, 13]. In this context, using isotope technique, Chapple et al. recently revealed that critically ill patients exhibited a markedly blunted muscle protein synthesis or anabolic resistance compared to healthy controls [38]. Notably, the incorporation of amino acids into the myofibrillar protein was 60% lower compared to a healthy control group. The reduced capacity to utilize protein during the acute phase of critical illness observed by Chapple et al., together with our findings of significantly higher serum and urinary urea as a result of higher protein provision, leads to a hypothesis that surplus protein may not be used for anabolism but is converted to urea for excretion. Higher urea levels may increase the metabolic burden of critically ill patients, particularly those with AKI, which may be one of the contributing factors to increased mortality in AKI patients, as demonstrated in our meta-analysis. Although a statistically significant mean increase of 2.31 mmol/L of serum urea or a mean increase of 5.55 g of urinary urea nitrogen may not be clinically significant in general, its clinical significance in critically ill patients with AKI remains unknown. Hence, the current findings have significant clinical implications, especially when considering the fact that current guidelines recommend higher protein delivery for critically ill patients with AKI, which should be carefully revised [39–41]. In contrast, the finding of non-statistically trend towards lowered mortality of higher protein delivery in patients with no/not known AKI requires further investigations.

Recent observational studies with robust statistical adjustments have examined protein delivery to critically ill patients during their first 5–7 days in the ICU. They have found that providing higher levels of protein, as opposed to medium or standard levels, does not lead to improved clinical outcomes and may even be harmful. One study by Hartl et al. involving 16,489 patients showed that protein delivery of 0.8–1.2 g/kg BW/d after 5 days of ICU admission resulted in lower hospital mortality compared to exclusively low protein intake (< 0.8 g/kg BW/d for ≤ 11 days). However, there was no further improvement in mortality when compared to early high protein intake (> 1.2 g/kg from day 1) [42]. Similarly, Matejovic et al. studied 1,172 patients with ≥ 5 days ICU-LOS and found that moderate nutrition dose (10–20 kcal/kg for energy and 0.8–1.2 g/kg for protein) improved patient weaning and reduced 90-day mortality compared to exclusively low nutrition intake (< 10 kcal/kg BW +  < 0.8 g/kg BW/d). Yet, there was no additional benefit when comparing moderate to high nutrition dose (> 20 kcal/kg BW/day +  > 1.2 g/kg BW/d) [43]. Lastly, Lin et al. studied 2,191 patients with ≥ 7 ICU-LOS and found that both high (1.68 g/kg BW/d) and low (0.38 g/kg BW/d) protein intake, compared to medium protein intake (0.8 g/kg BW/d), were associated with increased 28-day mortality [44]. Overall, these findings align with the conclusion that higher protein intake (around 1.5 g/kg BW/d) during the first week of critical illness does not offer additional benefits in improving clinical outcomes for critically ill patients.

While no significant differences in clinical outcomes were observed, higher protein delivery may help attenuate muscle loss. Combined with early physical rehabilitation, it could potentially improve long-term self-reported quality of life physical function score. In this context, a recent systematic review among healthy and non-critically ill patients found that higher protein was associated with increased lean body mass; however, the rate of lean body mass gain plateaued beyond 1.3 g/kg BW/day without resistance training [45]. It is plausible that certain subgroups of critically ill patients, particularly those who receive early physical rehabilitation, may experience greater muscle loss attenuation, ultimately enhancing their physical function. Similar findings were evident in ICU patients with traumatic brain injury, where those with greater quadriceps muscle thickness reported better physical function. [46] Another study linked greater lean mass with improved gait speed and 6-min walk distance in survivors of acute respiratory distress syndrome [47]. However, these objective outcomes were not assessed in the studies included in our systematic review. Nevertheless, the observed muscle loss attenuation may be a type-1 error, as indicated by TSA, underscoring the need for more studies to validate this finding. Similarly, the improvement in self-reported physical function scores with combination therapy only trended towards significance and primarily originated from small studies. Ongoing trials with combinations of high protein and early physical rehabilitation (excluding patients with AKI and not on kidney replacement therapy), such as the NEXIS (NCT03021902; registered 16 Jan 2017) and EFFORT-X (NCT04261543; registered 7 Feb 2020), which assess physical function outcomes with objective measures such as the 6-min walk test and short physical performance battery test, will provide further insights into the impact of higher protein delivery on physical function outcomes in non-AKI patients.

### Strength and limitations

The strength of our work lies in the comprehensive search and analysis and the predefined analysis plan for meta-analysis and TSA, all of which increase the transparency of information. In addition, excluding RCTs with different energy delivery between groups or pharmaconutrition interventions enabled us to focus solely on examining the effects of protein dosage. Furthermore, the use of TSA enabled us to detect the risk of type-1 or type-2 errors in our findings. The DARIS estimated from TSA will also inform the sample size needed for adequately powered future trials. Additionally, including extensive biochemical outcomes helped us elucidate the effects of higher protein delivery on metabolic parameters in critically ill patients.

Our work has several limitations. First, the included studies are heterogeneous in terms of the study population, dosage, timing, and routes of protein delivery. However, the included trials generally enrolled severely ill patients and primarily started intervention within 3 days of ICU admission. The subgroup analysis based on primarily EN vs exclusive PN/IV amino acids is consistent with the findings of the main analysis. The protein separation of approximately 0.49 g/kg BW/d with similar energy delivery between groups also ensures that the effect of protein was studied. Second, the three studies included in our analysis use varying definitions of AKI, which could limit the applicability of our findings in clinical practice. However, all the definitions identified AKI through an acute rise in serum creatinine levels. We recommend using the KDIGO definition of AKI [48] to guide protein delivery, as recent evidence showed that higher protein delivery is associated with increased mortality across all AKI stages, especially in patients who did not receive kidney replacement therapy [49]. Third, the number of studies with combinations of high protein and early physical rehabilitation intervention was limited, and the result is mainly attributed to one single-centre study with a high risk of bias [18]. Lastly, the certainty of evidence for most outcomes was assessed as low to very low due to the risk of bias and imprecision. Hence, more high-quality studies are warranted, especially studies with combined interventions (high protein and early physical rehabilitation).

## Conclusion

The present updated SRMA demonstrated that a higher protein delivery in the acute phase of critical illness has no effects on relevant clinical outcomes but significantly increased urea levels. Importantly, higher protein delivery increased mortality rates among AKI patients with high certainty, while its effect among non-AKI patients requires further investigation. In contrast, higher protein delivery may attenuate the loss of muscle mass, and the combination of high protein delivery and early physical rehabilitation may further improve self-reported physical function; however, these effects were only reported in a small number of studies of moderate to low quality. Future trials that combine high protein with early physical rehabilitation (in non-AKI patients) and assess objective physical function outcomes are warranted. Meanwhile, protein delivery should be carefully monitored in critically ill patients with AKI.

### Supplementary Information

**Additional file 1.** Supplementary methods, tables and figures.

## Data Availability

All data generated and/or analysed during the current study are included within the published article and its additional files.

## Abbreviations

AKI: Acute kidney injury
BW: Body weight
CCN: Critical care nutrition
CI: Confidence interval
EN: Enteral nutrition
GRADE: Grading of Recommendations Assessment, Development, and Evaluation
ICU: Intensive care unit
IV: Intravenous
LOS: Length of stay
MD: Mean difference
MV: Mechanical ventilation
PN: Parenteral nutrition
RCT: Randomized controlled trial
RR: Risk ratio
SMD: Standardized mean difference
SRMA: Systematic review and meta-analysis
TSA: Trial sequential analysis
