# Supplementary material for: The effects of higher versus lower protein delivery in critically ill patients: an updated systematic review and meta-analysis of randomized controlled trials with trial sequential analysis
Source: Crit Care. 2024 Jan 6;28:15. doi: 10.1186/s13054-023-04783-1 (PMC10770947; doi:10.1186/s13054-023-04783-1)
Supplement: Supplementary file 1 — Additional file 1. Supplementary methods, tables and figures. [file 13054_2023_4783_MOESM1_ESM.docx]

**Addiltional file 1**

**The Effects of Higher versus Lower Protein Delivery in Critically Ill Patients: An Updated Systematic Review and Meta-Analysis of Randomized Controlled Trials with Trial Sequential Analysis**

Zheng-Yii Lee^1,2^*, Ellen Dresen^3^*, Charles Chin Han Lew^4^*, Julia Bels^5,6^, Aileen Hill^7^, M. Shahnaz Hasan^1^, Lu Ke^8^, Arthur van Zanten^9^, Marcel CG van de Poll^5,6^, Daren Heyland^10^, Christian Stoppe^2,3^

*Co-first author

^1^Department of Anaesthesiology, Faculty of Medicine, University of Malaya, 50603 Kuala Lumpur, Malaysia.

^2^Department of Cardiac Anesthesiology & Intensive Care Medicine, Charit**é**Berlin, Germany

^3^ University Hospital Würzburg, Department of Anaesthesiology, Intensive Care, Emergency and Pain Medicine, Würzburg, Germany

^4^Department of Dietetics and Nutrition, Ng Teng Fong General Hospital, Singapore, Singapore 1 Jurong East Street 21, Singapore 609606

^5^Department of Intensive Care Medicine, Maastricht University Medical Centre, Maastricht 6229HX, The Netherlands

^6^NUTRIM School for Nutrition and Translational Research in Metabolism, Maastricht University, Universiteitssingel 40, 6229 ER Maastricht, The Netherlands

^7^Department of Anesthesiology and Department of Intensive Care Medicine, University Hospital RWTH Aachen, Aachen, Germany

^8^Department of Critical Care Medicine, Jinling Hospital, Medical School of Nanjing University, No. 305 Zhongshan East Road, Nanjing, 210000, Jiangsu Province, China

^9^Department of Intensive Care Medicine, Gelderse Vallei Hospital, Ede & Wageningen University & Research, Wageningen, The Netherlands

^10^Clinical Evaluation Research Unit, Department of Critical Care Medicine, Queen’s University, Kingston, ON, K7L 3N6, Canada

**Correspondence to:**

**Zheng-Yii Lee**

Department of Anesthesiology, Faculty of Medicine, University of Malaya, Kuala Lumpur, Malaysia.

Department of Cardiac Anesthesiology & Intensive Care Medicine, Charité Berlin, Berlin, Germany.

Email: zheng_yii@hotmail.com

ORCID: 0000-0003-4505-7476

Contents

[Supplementary Methods 6](#_Toc149813678)

[Data handling 6](#_Toc149813679)

[Outcomes 6](#_Toc149813680)

[Trial Sequential Analysis 7](#_Toc149813681)

[PRISMA Checklist 8](#_Toc149813682)

[Table S1 Search Strategy 11](#_Toc149813683)

[a) Ovid MEDLINE(R) and Epub Ahead of Print, In-Process, In-Data-Review & Other Non-Indexed Citations and Daily <1946 to May 25, 2023> 11](#_Toc149813684)

[b) Embase Classic+Embase <1947 to 2023 May 25> 12](#_Toc149813685)

[c) EBM Reviews - Cochrane Central Register of Controlled Trials <April 2023> 14](#_Toc149813686)

[Table S2: Clinical Trial Registry of Ongoing Studies 15](#_Toc149813687)

[Table S3 Critical Care Nutrition Methodological Quality Scoring System 18](#_Toc149813688)

[Table S4: List of Excluded Studies, Reason for Exclusion and Study Source 19](#_Toc149813689)

[Table S5: Patients’ Baseline Characteristics of Included Studies 23](#_Toc149813690)

[Table S6: Nutritional Prescription, Prescription body weight and Energy and Protein Delivered 26](#_Toc149813691)

[Table S7: Details of the Early Physical Rehabilitation Intervention 30](#_Toc149813692)

[Table S8: Critical Care Nutrition Methodological Quality Scores of all the Included studies 31](#_Toc149813693)

[Table S9: Outcomes summary 33](#_Toc149813694)

[Table S10: Biochemical Outcomes Summary 39](#_Toc149813695)

[Figure S1: PRISMA 2020 Flow Diagram 46](#_Toc149813696)

[Figure S2 Protein (g/kg/day) and Energy (kcal/kg/day) delivered between higher vs lower 47](#_Toc149813697)

[Figure S3 ROB2 Traffic Light and Summary Plots 48](#_Toc149813698)

[a) Overall Mortality 48](#_Toc149813699)

[b) Infectious Complications 49](#_Toc149813700)

[c) Duration of mechanical ventilation 50](#_Toc149813701)

[d) ICU length of stay 51](#_Toc149813702)

[e) Hospital length of stay 52](#_Toc149813703)

[f) Muscle mass 53](#_Toc149813704)

[g) Muscle strength 53](#_Toc149813705)

[h) Discharge to rehab facilities 54](#_Toc149813706)

[i) Self-reported Quality of life physical measures at day 90 54](#_Toc149813707)

[j) Incidence of Diarrhea 55](#_Toc149813708)

[Figure S4 Funnel plots 56](#_Toc149813709)

[a) Overall mortality 56](#_Toc149813710)

[b) Infectious complications 57](#_Toc149813711)

[c) Duration of mechanical ventilation 58](#_Toc149813712)

[d) ICU length of stay 59](#_Toc149813713)

[e) Hospital length of stay 60](#_Toc149813714)

[f) Muscle mass 61](#_Toc149813715)

[g) Handgrip strength 62](#_Toc149813716)

[h) Discharge to Rehabilitation Facilities 62](#_Toc149813717)

[i) Self-reported Quality of life Physical Measure at day 90 63](#_Toc149813718)

[Figure S5 ICU, Hospital, 28- and ≥60-day Mortality and additional AKI subgroup analysis 64](#_Toc149813719)

[a) ICU Mortality 64](#_Toc149813720)

[b) Hospital Mortality 64](#_Toc149813721)

[c) 28-day Mortality 65](#_Toc149813722)

[d) ≥60-day Mortality 65](#_Toc149813723)

[e) Additional AKI subgroup analysis (risk difference) 66](#_Toc149813724)

[Figure S6: Biochemical Outcomes 67](#_Toc149813725)

[a) Serum urea (mmol/L) 67](#_Toc149813726)

[b) Urinary Urea Nitrogen (g) 67](#_Toc149813727)

[c) Nitrogen balance (g) – Ferrie D7 67](#_Toc149813728)

[d) Nitrogen balance (g) – Ferrie D3 67](#_Toc149813729)

[e) Serum Creatinine (umol/L) 67](#_Toc149813730)

[f) Blood glucose (mmol/L) 68](#_Toc149813731)

[g) Insulin (unit) 68](#_Toc149813732)

[h) Serum albumin (g/L) 68](#_Toc149813733)

[i) Prealbumin (mg/dL) 68](#_Toc149813734)

[j) Hemoglobin (g/L) 68](#_Toc149813735)

[k) White blood cells (10^9^/L) 69](#_Toc149813736)

[l) Lymphocyte count (cells per µL of blood) 69](#_Toc149813737)

[m) C-reactive protein (mg/dL) 69](#_Toc149813738)

[n) Interleukin 6 (ng/L) 69](#_Toc149813739)

[o) Phosphate level (mmol/L) 69](#_Toc149813740)

[p) Triglycerides (mmol/L) 69](#_Toc149813741)

[Figure S7 Subgroup analysis (low vs other ROB) 70](#_Toc149813742)

[a) Overall mortality 70](#_Toc149813743)

[b) Infectious complications (all studies had some concerns) 70](#_Toc149813744)

[c) ICU LOS 70](#_Toc149813745)

[d) Hospital LOS 71](#_Toc149813746)

[e) Length of mechanical ventilation 71](#_Toc149813747)

[f) Muscle wasting per week 72](#_Toc149813748)

[g) Handgrip strength (all studies were high risk of bias) 72](#_Toc149813749)

[h) Discharge to rehabilitation facilities 72](#_Toc149813750)

[i) Self-reported Quality of life physical function at day 90 72](#_Toc149813751)

[j) Incidence of Diarrhea 73](#_Toc149813752)

[Figure S8 Subgroup analysis (single vs multicenter studies) 74](#_Toc149813753)

[a) Overall mortality 74](#_Toc149813754)

[b) Infectious complications (no multicenter studies reported this outcome) 74](#_Toc149813755)

[c) Duration of mechanical ventilation 74](#_Toc149813756)

[d) ICU length of stay 75](#_Toc149813757)

[e) Hospital length of stay 75](#_Toc149813758)

[f) Muscle mass (no multicenter studies reported this outcome) 75](#_Toc149813759)

[g) Handgrip strength (no multicenter studies reported this outcome) 75](#_Toc149813760)

[h) Discharge to rehabilitation facilities 76](#_Toc149813761)

[i) Self-reported Quality of life Physical Measure at day 90 76](#_Toc149813762)

[j) Incidence of diarrhea 76](#_Toc149813763)

[Figure S9 Trial sequential analysis graph 77](#_Toc149813764)

[TSA of other outcomes 77](#_Toc149813765)

[a) Muscle wasting per week (5 studies, n=273) 77](#_Toc149813766)

[b) Handgrip strength (2 studies, n=130) 77](#_Toc149813767)

[c) Incidence of diarrhoea (6 studies, n=662) 78](#_Toc149813768)

[References 79](#_Toc149813769)

## Supplementary Methods

### Data handling

For studies that reported median (Q1–Q3) for continuous outcomes, we contacted the authors to obtain the mean and standard deviation (SD). If the means and SDs were unavailable, we excluded those outcomes from the meta-analysis. Median and range were not converted to mean and SD for meta-analysis.

For nutrition variables, in some cases, the precise estimates were unavailable as data were only presented in graphs and the authors could not provide further information. In this case, amounts of nutrition delivery were estimated from the graph but not included in the meta-analysis. Protein and energy delivery from individual studies was pooled into a single mean and SD (by group) using an online calculator.^1^ Authors were contacted (up to three times) to obtain additional data where needed.

In one included study,^2^ we only included 2 out of the 3 groups randomized to similar energy but different protein doses in our meta-analysis. This study also reported the nutritional delivery and LOS outcomes separately for traumatic brain injury (TBI) and non-TBI group, and these were pooled into a single mean and SD in our meta-analysis. In another study,^3^ patients were randomized to the use of indirect calorimetry or predictive equation and further randomized to higher versus lower protein group. These data were pooled into two groups based on the protein dosage by simple addition (categorical outcomes) or using the online calculator (continuous outcomes).

### Outcomes

Outcomes with at least 2 studies were pooled and reported. Specifically (i) nutritional outcomes: average protein (g/kg BW/day and g/day) and energy (kcal/kg BW/day) for the individual study duration; (ii) clinical outcomes: overall mortality (if a study reported multiple timepoints, we pooled one of the mortality outcomes in the following order: 28-day, hospital, ICU and other mortality), and ICU, hospital, 28-day, and ≥ 60-day mortality (we selected the longest duration), infectious complications, ICU and hospital LOS and duration of mechanical ventilation (MV); (iii) muscle outcomes: percentage change of muscle mass and handgrip strength; (iv) discharge to rehabilitation facilities, (v) Quality of life (QOL) physical measurements, and (vi) biochemical outcomes: serum urea (mmol/L), urinary urea nitrogen (g), nitrogen balance (g), creatinine (µmol/L), blood glucose (mmol/L), insulin (unit), albumin (g/L), prealbumin (mg/dL), haemoglobin (g/L), white blood count (10^9^/L), lymphocyte (cells per µL of blood), C-reactive protein (CRP) (mg/dL), interlukin-6 (IL-6) (ng/L), phosphate (mmol/L), and triglycerides (mmol/L). If a study reported the mean value of the biochemical outcomes at multiple timepoints, the timepoint with the longest follow-up was chosen, unless specified otherwise.

### Trial Sequential Analysis

The following parameters for trial sequential analysis (TSA) were pre-specified: alpha 5%, beta 10% (power 90%), and the DerSimonian–Laird random effect model.^4^ Between-trial heterogeneity was adjusted by the diversity-estimate (D^2^). A continuity correction factor of 0.5 was added in case of zero events. The effect sizes for categorical outcomes (mortality, infectious complications, and diarrhoea) were 10%, and this was based on a previous meta-analysis^5^ or a clinically meaningful and realistic magnitude. *Post-hoc*, in patients with AKI, the relative risk reduction for mortality between lower vs. higher protein was set at 46%.^6^ For continuous outcomes, the minimal important difference was based on a magnitude of meaningful change for the patient and/or clinician, which were 1 day for the duration of mechanical ventilation, ICU and hospital length of stay, 1% for muscle loss changes per week,^7^ and 5 kg for handgrip strength changes per week.^8^ For categorical outcomes, the proportion of events in the control population was based on the pooled observed event rate of the updated meta-analysis. For continuous outcomes, the variance was based on the pooled observed standard deviation of the current updated meta-analysis. This approach will maximize the generalizability of the results.

PRISMA Checklist

| **Section and Topic** | **Item #** | **Checklist item** | **Location where item is reported** |
| --- | --- | --- | --- |
| **TITLE** | | |  |
| Title | 1 | Identify the report as a systematic review. | Page 1 |
| **ABSTRACT** | | |  |
| Abstract | 2 | See the PRISMA 2020 for Abstracts checklist. | Page 3-4 |
| **INTRODUCTION** | | |  |
| Rationale | 3 | Describe the rationale for the review in the context of existing knowledge. | Page 5-6 |
| Objectives | 4 | Provide an explicit statement of the objective(s) or question(s) the review addresses. | Page 6 |
| **METHODS** | | |  |
| Eligibility criteria | 5 | Specify the inclusion and exclusion criteria for the review and how studies were grouped for the syntheses. | Page 7 |
| Information sources | 6 | Specify all databases, registers, websites, organisations, reference lists and other sources searched or consulted to identify studies. Specify the date when each source was last searched or consulted. | Page 7-8 and Table S1 |
| Search strategy | 7 | Present the full search strategies for all databases, registers and websites, including any filters and limits used. | Table S1 |
| Selection process | 8 | Specify the methods used to decide whether a study met the inclusion criteria of the review, including how many reviewers screened each record and each report retrieved, whether they worked independently, and if applicable, details of automation tools used in the process. | Page 8 |
| Data collection process | 9 | Specify the methods used to collect data from reports, including how many reviewers collected data from each report, whether they worked independently, any processes for obtaining or confirming data from study investigators, and if applicable, details of automation tools used in the process. | Page 8 and supplement page 6 |
| Data items | 10a | List and define all outcomes for which data were sought. Specify whether all results that were compatible with each outcome domain in each study were sought (e.g. for all measures, time points, analyses), and if not, the methods used to decide which results to collect. | Page 8 & Supplement Page 6 |
|  | 10b | List and define all other variables for which data were sought (e.g. participant and intervention characteristics, funding sources). Describe any assumptions made about any missing or unclear information. | Supplement Page 6 |
| Study risk of bias assessment | 11 | Specify the methods used to assess risk of bias in the included studies, including details of the tool(s) used, how many reviewers assessed each study and whether they worked independently, and if applicable, details of automation tools used in the process. | Page 8 |
| Effect measures | 12 | Specify for each outcome the effect measure(s) (e.g. risk ratio, mean difference) used in the synthesis or presentation of results. | Page 9 |
| Synthesis methods | 13a | Describe the processes used to decide which studies were eligible for each synthesis (e.g. tabulating the study intervention characteristics and comparing against the planned groups for each synthesis (item #5)). | Page 9-10 |
|  | 13b | Describe any methods required to prepare the data for presentation or synthesis, such as handling of missing summary statistics, or data conversions. | Supplement Page 6 |
|  | 13c | Describe any methods used to tabulate or visually display results of individual studies and syntheses. | Page 9-10 |
|  | 13d | Describe any methods used to synthesize results and provide a rationale for the choice(s). If meta-analysis was performed, describe the model(s), method(s) to identify the presence and extent of statistical heterogeneity, and software package(s) used. | Page 10 |
|  | 13e | Describe any methods used to explore possible causes of heterogeneity among study results (e.g. subgroup analysis, meta-regression). | Page 9-10 |
|  | 13f | Describe any sensitivity analyses conducted to assess robustness of the synthesized results. | N/A |
| Reporting bias assessment | 14 | Describe any methods used to assess risk of bias due to missing results in a synthesis (arising from reporting biases). | Page 8 |
| Certainty assessment | 15 | Describe any methods used to assess certainty (or confidence) in the body of evidence for an outcome. | Page 10-11 |
| **RESULTS** | | |  |
| Study selection | 16a | Describe the results of the search and selection process, from the number of records identified in the search to the number of studies included in the review, ideally using a flow diagram. | Page 12 |
|  | 16b | Cite studies that might appear to meet the inclusion criteria, but which were excluded, and explain why they were excluded. | Table S4 |
| Study characteristics | 17 | Cite each included study and present its characteristics. | Table 1 |
| Risk of bias in studies | 18 | Present assessments of risk of bias for each included study. | Table S8 and Figure S3 |
| Results of individual studies | 19 | For all outcomes, present, for each study: (a) summary statistics for each group (where appropriate) and (b) an effect estimate and its precision (e.g. confidence/credible interval), ideally using structured tables or plots. | Page 12-13; Figure 1 & 2, Figure S5-S8 |
| Results of syntheses | 20a | For each synthesis, briefly summarise the characteristics and risk of bias among contributing studies. | Page 13-14 & Figure S3 |
|  | 20b | Present results of all statistical syntheses conducted. If meta-analysis was done, present for each the summary estimate and its precision (e.g. confidence/credible interval) and measures of statistical heterogeneity. If comparing groups, describe the direction of the effect. | Page 14-16 |
|  | 20c | Present results of all investigations of possible causes of heterogeneity among study results. | Page 14 & 16 |
|  | 20d | Present results of all sensitivity analyses conducted to assess the robustness of the synthesized results. | N/A |
| Reporting biases | 21 | Present assessments of risk of bias due to missing results (arising from reporting biases) for each synthesis assessed. | Page 13, Table S8, Figure S3-S4 |
| Certainty of evidence | 22 | Present assessments of certainty (or confidence) in the body of evidence for each outcome assessed. | Page 17, Table 3 |
| **DISCUSSION** | | |  |
| Discussion | 23a | Provide a general interpretation of the results in the context of other evidence. | Page 17-20 |
|  | 23b | Discuss any limitations of the evidence included in the review. | Page 21 |
|  | 23c | Discuss any limitations of the review processes used. | Page 21 |
|  | 23d | Discuss implications of the results for practice, policy, and future research. | Page 18, 19, 23 |
| **OTHER INFORMATION** | | |  |
| Registration and protocol | 24a | Provide registration information for the review, including register name and registration number, or state that the review was not registered. | Page 2 |
|  | 24b | Indicate where the review protocol can be accessed, or state that a protocol was not prepared. | Page 2 |
|  | 24c | Describe and explain any amendments to information provided at registration or in the protocol. | N/A |
| Support | 25 | Describe sources of financial or non-financial support for the review, and the role of the funders or sponsors in the review. | Page 25 |
| Competing interests | 26 | Declare any competing interests of review authors. | Page 25 |
| Availability of data, code and other materials | 27 | Report which of the following are publicly available and where they can be found: template data collection forms; data extracted from included studies; data used for all analyses; analytic code; any other materials used in the review. | Page 25 |

## Table S1 Search Strategy

### a) Ovid MEDLINE(R) and Epub Ahead of Print, In-Process, In-Data-Review & Other Non-Indexed Citations and Daily <1946 to May 25, 2023>

| **#** | **Query** | **Results from 29 May 2023** |
| --- | --- | --- |
| 1 | randomized controlled trial.pt. | 593,339 |
| 2 | controlled clinical trial.pt. | 95,316 |
| 3 | randomized.ab. | 604,411 |
| 4 | placebo.ab. | 238,467 |
| 5 | drug therapy.fs. | 2,593,334 |
| 6 | randomly.ab. | 408,919 |
| 7 | trial.ab. | 649,479 |
| 8 | groups.ab. | 2,521,064 |
| 9 | or/1-8 | 5,664,867 |
| 10 | exp animals/ not humans.sh. | 5,124,207 |
| 11 | 9 not 10 | 4,943,083 |
| 12 | Critical care/ | 60,237 |
| 13 | critical care.mp. | 88,142 |
| 14 | intensive care.mp. | 226,466 |
| 15 | Critical illness/ | 38,409 |
| 16 | critical illness.mp. | 44,991 |
| 17 | critically ill.mp. | 59,247 |
| 18 | Intensive care units/ or burn units/ or coronary care units/ or respiratory care units/ | 77,044 |
| 19 | intensive care unit*.mp. | 191,008 |
| 20 | critical care unit*.mp. | 4,108 |
| 21 | exp Shock/ | 85,593 |
| 22 | sepsis/ or bacteremia/ or fungemia/ | 98,730 |
| 23 | systemic inflammatory response syndrome.mp. | 11,003 |
| 24 | sepsis.mp. | 148,847 |
| 25 | septic shock.mp. | 27,337 |
| 26 | multiple organ dysfunction syndrome.mp. | 2,692 |
| 27 | multiple organ failure.mp. | 17,773 |
| 28 | cytokine release syndrome.mp. | 4,094 |
| 29 | Respiratory Distress Syndrome/ | 24,507 |
| 30 | Severe Acute Respiratory Syndrome/ | 5,730 |
| 31 | respiratory distress syndrome.mp. | 55,382 |
| 32 | acute lung injury.mp. | 18,550 |
| 33 | Burns/ | 48,400 |
| 34 | (burn$ adj3 patient$).mp. | 17,530 |
| 35 | Multiple Trauma/ | 13,415 |
| 36 | Multi* Trauma.mp. | 15,839 |
| 37 | Abdominal Injuries/ | 15,385 |
| 38 | Abdom*n* injur*.mp. | 17,128 |
| 39 | Pancreatitis, Acute Necrotizing/ | 3,802 |
| 40 | Acute Necroti*ing Pancreatitis.mp. | 1,323 |
| 41 | brain injuries/ or brain hemorrhage, traumatic/ or brain injuries, diffuse/ or brain injuries, traumatic/ | 67,879 |
| 42 | Head Injuries, Closed/ | 3,205 |
| 43 | Brain Injur*.mp. | 111,121 |
| 44 | Head injur*.mp. | 29,701 |
| 45 | or/12-44 | 774,879 |
| 46 | nutritional support/ | 7,125 |
| 47 | enteral nutrition/ | 21,869 |
| 48 | parenteral nutrition/ | 16,314 |
| 49 | Parenteral Nutrition, Total/ | 10,385 |
| 50 | Intubation, Gastrointestinal/ | 10,231 |
| 51 | (nutrition$ adj3 support$).mp. | 19,772 |
| 52 | artificial nutrition.mp. | 1,379 |
| 53 | enteral nutrition.mp. | 26,083 |
| 54 | enteric feeding.mp. | 88 |
| 55 | parenteral nutrition.mp. | 34,251 |
| 56 | intravenous feeding.mp. | 467 |
| 57 | Gastrointestin* intubation.mp. | 53 |
| 58 | or/46-57 | 74,765 |
| 59 | Amino Acids/ | 146,294 |
| 60 | Peptides/ | 173,894 |
| 61 | Proteins/ or exp dietary proteins/ or protein hydrolysates/ | 323,510 |
| 62 | Protein*.mp. | 5,407,776 |
| 63 | amino acid*.mp. | 986,991 |
| 64 | peptide*.mp. | 908,248 |
| 65 | or/59-64 | 5,998,016 |
| 66 | 11 and 45 and 58 and 65 | 1,156 |
| 67 | limit 66 to dt=20210401-20230529 | 120 |
| 68 | limit 66 to rd=20210401-20230529 | 391 |
| 69 | 67 or 68 | 391 |

### b) Embase Classic+Embase <1947 to 2023 May 25>

| **#** | **Query** | **Results from 29 May 2023** |
| --- | --- | --- |
| 1 | Randomized controlled trial/ | 788,578 |
| 2 | Controlled clinical study/ | 469,623 |
| 3 | random$.ti,ab. | 1,981,362 |
| 4 | randomization/ | 99,460 |
| 5 | intermethod comparison/ | 297,400 |
| 6 | placebo.ti,ab. | 371,225 |
| 7 | (compare or compared or comparison).ti. | 635,147 |
| 8 | ((evaluated or evaluate or evaluating or assessed or assess) and (compare or compared or comparing or comparison)).ab. | 2,774,918 |
| 9 | (open adj label).ti,ab. | 109,052 |
| 10 | ((double or single or doubly or singly) adj (blind or blinded or blindly)).ti,ab. | 280,099 |
| 11 | double blind procedure/ | 213,168 |
| 12 | parallel group$1.ti,ab. | 32,267 |
| 13 | (crossover or cross over).ti,ab. | 125,950 |
| 14 | ((assign$ or match or matched or allocation) adj5 (alternate or group$1 or intervention$1 or patient$1 or subject$1 or participant$1)).ti,ab. | 417,487 |
| 15 | (assigned or allocated).ti,ab. | 491,973 |
| 16 | (controlled adj7 (study or design or trial)).ti,ab. | 454,826 |
| 17 | (volunteer or volunteers).ti,ab. | 288,594 |
| 18 | human experiment/ | 651,776 |
| 19 | trial.ti. | 411,431 |
| 20 | or/1-19 | 6,391,045 |
| 21 | (random$ adj sampl$ adj7 (cross section$ or questionnaire$1 or survey$ or database$1)).ti,ab. not (comparative study/ or controlled study/ or randomi?ed controlled.ti,ab. or randomly assigned.ti,ab.) | 9,566 |
| 22 | Cross-sectional study/ not (randomized controlled trial/ or controlled clinical study/ or controlled study/ or randomi?ed controlled.ti,ab. or control group$1.ti,ab.) | 347,814 |
| 23 | (((case adj control$) and random$) not randomi?ed controlled).ti,ab. | 21,607 |
| 24 | (Systematic review not (trial or study)).ti. | 260,811 |
| 25 | (nonrandom$ not random$).ti,ab. | 19,058 |
| 26 | Random field$.ti,ab. | 2,951 |
| 27 | (random cluster adj3 sampl$).ti,ab. | 1,542 |
| 28 | (review.ab. and review.pt.) not trial.ti. | 1,117,857 |
| 29 | we searched.ab. and (review.ti. or review.pt.) | 49,790 |
| 30 | update review.ab. | 138 |
| 31 | (databases adj4 searched).ab. | 62,434 |
| 32 | (rat or rats or mouse or mice or swine or porcine or murine or sheep or lambs or pigs or piglets or rabbit or rabbits or cat or cats or dog or dogs or cattle or bovine or monkey or monkeys or trout or marmoset$1).ti. and animal experiment/ | 1,227,348 |
| 33 | Animal experiment/ not (human experiment/ or human/) | 2,581,423 |
| 34 | or/21-33 | 4,337,526 |
| 35 | 20 not 34 | 5,649,736 |
| 36 | Intensive care/ | 147,875 |
| 37 | critical care.mp. | 74,074 |
| 38 | intensive care.mp. | 480,038 |
| 39 | critical illness/ | 35,712 |
| 40 | critical illness.mp. | 44,214 |
| 41 | critically ill.mp. | 105,602 |
| 42 | intensive care unit/ or burn unit/ or coronary care unit/ or medical intensive care unit/ or neurological intensive care unit/ or stroke unit/ or surgical intensive care unit/ | 255,573 |
| 43 | intensive care unit*.mp. | 335,343 |
| 44 | critical care unit*.mp. | 7,480 |
| 45 | exp shock/ | 180,002 |
| 46 | systemic inflammatory response syndrome/ | 15,949 |
| 47 | sepsis/ or bacteremia/ or fungemia/ or septicemia/ or urosepsis/ | 276,741 |
| 48 | multiple organ failure/ | 49,418 |
| 49 | systemic inflammatory response syndrome.mp. | 19,150 |
| 50 | sepsis.mp. | 276,854 |
| 51 | septic shock.mp. | 76,334 |
| 52 | multiple organ dysfunction syndrome.mp. | 3,814 |
| 53 | multiple organ failure.mp. | 53,611 |
| 54 | cytokine release syndrome.mp. | 9,103 |
| 55 | respiratory distress syndrome/ or acute lung injury/ or adult respiratory distress syndrome/ or transfusion related acute lung injury/ | 88,675 |
| 56 | Severe Acute Respiratory Syndrome/ | 11,746 |
| 57 | respiratory distress syndrome.mp. | 95,810 |
| 58 | acute lung injury.mp. | 30,553 |
| 59 | burn/ or burn shock/ | 72,597 |
| 60 | (burn$ adj3 patient$).mp. | 25,881 |
| 61 | Multiple Trauma/ | 17,648 |
| 62 | Multi* Trauma.mp. | 20,224 |
| 63 | exp abdominal injury/ | 211,311 |
| 64 | Abdom*n* injur*.mp. | 20,070 |
| 65 | pancreatitis/ or acute hemorrhagic pancreatitis/ or acute pancreatitis/ | 105,849 |
| 66 | Acute Necroti*ing Pancreatitis.mp. | 2,131 |
| 67 | brain injury/ or acquired brain injury/ or brain concussion/ or brain contusion/ or brain damage/ or brain stem injury/ or cerebellum injury/ or diffuse brain injury/ or traumatic brain injury/ | 221,679 |
| 68 | head injury/ | 59,888 |
| 69 | Brain Injur*.mp. | 196,896 |
| 70 | Head injur*.mp. | 72,137 |
| 71 | or/36-70 | 1,711,417 |
| 72 | nutritional support/ | 22,949 |
| 73 | exp artificial feeding/ | 101,884 |
| 74 | (nutrition$ adj3 support$).mp. | 35,296 |
| 75 | artificial nutrition.mp. | 2,308 |
| 76 | enteral nutrition.mp. | 19,274 |
| 77 | enteric feeding.mp. | 40,713 |
| 78 | parenteral nutrition.mp. | 58,929 |
| 79 | intravenous feeding.mp. | 1,842 |
| 80 | Gastrointestin* intubation.mp. | 205 |
| 81 | or/72-80 | 134,248 |
| 82 | Amino Acids/ | 207,382 |
| 83 | Peptides/ | 130,376 |
| 84 | exp protein diet/ | 8,894 |
| 85 | protein intake/ | 50,279 |
| 86 | Protein*.mp. | 7,288,831 |
| 87 | amino acid*.mp. | 952,306 |
| 88 | peptide*.mp. | 927,593 |
| 89 | or/82-88 | 7,931,393 |
| 90 | 35 and 71 and 81 and 89 | 1,888 |
| 91 | limit 90 to dd=20210401-20230529 | 91 |
| 92 | limit 90 to rd=20210401-20230529 | 259 |
| 93 | 91 or 92 | 350 |

### c) EBM Reviews - Cochrane Central Register of Controlled Trials <April 2023>

| **#** | **Query** | **Results from 29 May 2023** |
| --- | --- | --- |
| 1 | Critical care/ | 2,236 |
| 2 | critical care.mp. | 4,868 |
| 3 | intensive care.mp. | 29,206 |
| 4 | Critical illness/ | 3,172 |
| 5 | critical illness.mp. | 4,418 |
| 6 | critically ill.mp. | 8,332 |
| 7 | Intensive care units/ or burn units/ or coronary care units/ or respiratory care units/ | 3,439 |
| 8 | intensive care unit*.mp. | 23,494 |
| 9 | critical care unit*.mp. | 438 |
| 10 | exp Shock/ | 3,248 |
| 11 | sepsis/ or bacteremia/ or fungemia/ | 4,279 |
| 12 | systemic inflammatory response syndrome.mp. | 1,239 |
| 13 | sepsis.mp. | 12,908 |
| 14 | septic shock.mp. | 3,571 |
| 15 | multiple organ dysfunction syndrome.mp. | 269 |
| 16 | multiple organ failure.mp. | 1,605 |
| 17 | cytokine release syndrome.mp. | 352 |
| 18 | Respiratory Distress Syndrome/ | 1,881 |
| 19 | Severe Acute Respiratory Syndrome/ | 380 |
| 20 | respiratory distress syndrome.mp. | 6,438 |
| 21 | acute lung injury.mp. | 1,424 |
| 22 | Burns/ | 1,737 |
| 23 | (burn$ adj3 patient$).mp. | 2,352 |
| 24 | Multiple Trauma/ | 290 |
| 25 | Multi* Trauma.mp. | 647 |
| 26 | Abdominal Injuries/ | 163 |
| 27 | Abdom*n* injur*.mp. | 281 |
| 28 | Pancreatitis, Acute Necrotizing/ | 166 |
| 29 | Acute Necroti*ing Pancreatitis.mp. | 87 |
| 30 | brain injuries/ or brain hemorrhage, traumatic/ or brain injuries, diffuse/ or brain injuries, traumatic/ | 2,718 |
| 31 | Head Injuries, Closed/ | 84 |
| 32 | Brain Injur*.mp. | 7,599 |
| 33 | Head injur*.mp. | 1,813 |
| 34 | or/1-33 | 64,159 |
| 35 | nutritional support/ | 485 |
| 36 | enteral nutrition/ | 2,117 |
| 37 | parenteral nutrition/ | 1,223 |
| 38 | Parenteral Nutrition, Total/ | 800 |
| 39 | Intubation, Gastrointestinal/ | 739 |
| 40 | (nutrition$ adj3 support$).mp. | 3,136 |
| 41 | artificial nutrition.mp. | 105 |
| 42 | enteral nutrition.mp. | 4,924 |
| 43 | enteric feeding.mp. | 1,776 |
| 44 | parenteral nutrition.mp. | 4,386 |
| 45 | intravenous feeding.mp. | 126 |
| 46 | Gastrointestin* intubation.mp. | 9 |
| 47 | or/35-46 | 11,305 |
| 48 | Amino Acids/ | 1,983 |
| 49 | Peptides/ | 2,089 |
| 50 | Proteins/ or exp dietary proteins/ or protein hydrolysates/ | 5,695 |
| 51 | Protein*.mp. | 118,049 |
| 52 | amino acid*.mp. | 10,273 |
| 53 | peptide*.mp. | 27,793 |
| 54 | or/48-53 | 144,072 |
| 55 | 34 and 47 and 54 | 1,106 |
| 56 | limit 55 to yr="2021 -Current" | 113 |

## Table S2: Clinical Trial Registry of Ongoing Studies

| **No** | **Trial Registration Number** | **Country** | **Title** | **Acronym** | **N** | **Intervention** | **Control** | **Primary outcome** | **Last Update** | **Status** |
| --- | --- | --- | --- | --- | --- | --- | --- | --- | --- | --- |
| **1** | NCT02865408 | Canada | Amino Acid Nutrition in the Critically-ill | AA-ICU | 30 | 1) 2.5 g/kg/d IV amino acid and EN  2) 1.75 g/kg/d IV amino acid and EN | 1.0 g/kg/d EN | Whole body protein balance (0-48h) | April 12,2022 | Recruiting |
| **2** | NCT03170401 | USA | Supplemental Enteral Protein in Critical Illness |  | 500 | EN with additional protein  supplementation | No protein supplementation | Serum concentrations of transthyretin at 3 weeks after injury. | March 31, 2013 | Completed |
| **3** | NCT05918757 | Spain | Efficacy and Safety of Administration of High Levels of Protein to Critically Ill Patients | FISIO | 200 | Administration of 1.5 g of protein/kg/day via enteral/parenteral nutrition | Administration of 1.0 g of protein/kg/day via enteral/parenteral nutrition | Change of intensive care unit acquired weakness (ICUAW) | June 26, 2023 | Recruiting |
| **4** | NCT04475666 | Saudi Arabia | Replacing Protein Via Enteral Nutrition in Critically Ill Patients | REPLENISH | 2502 | Standard amount of protein (max 1.2 g/kg/d) + supplemental protein at 1.2 g/kg/d | Standard amount of protein (max 1.2 g/kg/d) only. | 90 day-all cause mortality | January 10, 2023 | Recruiting |
| **5** | NCT05647135 | Romania | ImpACt of Very High Protein Content Enteral nUtrition Formulas on Critically Ill MUltipLe trAuma paTiEnts | ACCUMULATE | 70 | Enteral formula with 10 g/100 ml protein | Enteral formula with 6.3 g/100 ml protein | Differences regarding achieving protein and calorie daily targets in grams per kilogram of body weight (g/kg BW) at day 5 of the intervention period and at day 10 when using enteral nutrition formulas with different protein content | December 12, 2022 | Not yet recruiting |
| **6** | NCT04633421 | Netherlands | PRotEin Provision in Critical IllneSs | PRECISe | 824 | EN feed with 8g protein/100 kcal (target 2.0 g/kg/d) | EN feed with 5g protein/100 kcal (target 1.2 g/kg/d) | Health Related Quality of Life (HRQL) | April 18, 2023 | Active, Not Recruiting |
| **7** | ACTRN12621001484831 | Australia | TARGET Protein: The effect of augmented administration of enteral protein to critically ill adults on out of hospital survival: A cluster randomised, cross-sectional double cross-over, registry-embedded, pragmatic clinical trial | TARGET Protein | 3000 | Enteral formula with 1.26 kcal/ml and 100g protein per 1000 ml delivered via naso-enteric tube. The maximum goal rate is 1 ml/kg ideal body weight (IBW / hour), delivered over 24 hours/day, for up to 90 days | Enteral formula with 1.25 kcal/ml and 63g protein per 1000 ml delivered via naso-enteric tube. The maximum goal rate is 1 ml/kg ideal body weight (IBW / hour), delivered over 24 hours/day, for up to 90 days | Days free of the index hospital and alive at day-90 | Sept 18, 2023 | Active, Not Recruiting |
| **Studies that combined higher protein and early mobility/exercise** | | | | | | | | | | |
| **8** | NCT02509520 | USA | Assessing The Effects of Exercise, Protein, and Electric Stimulation On Intensive Care Unit Patients Outcomes | ExPrEs | 60 | Mobility-based Physical Rehab + High protein | Mobility-based Physical Rehab only | Muscle mass strength and physical function | Feb 10, 2023 | Active, not recruiting |
| **9** | NCT03021902 | Canada/ USA | Nutrition and Exercise in Critical Illness | NEXIS | 142 | 2.0-2.5 g/kg/d IV amino acids + in-bed cycle ergometry | Usual care | 6 minute walk distance at hospital discharge | Sept 5, 2021 | Recruiting |
| **10** | NCT05197231 | Sweden | Investigating the Anabolic Response to Resistance Exercise During Critical Illness | ARTIST-1 | 24 | Resisted knee extension exercise and IV amino acids delivered by continuous infusion at a rate of 0.1 g/kg/h | IV amino acids delivered by continuous infusion at a rate of 0.1 g/kg/h | Between-group difference in change in lower limb protein balance | June 06, 2022 | Recruiting |
| **11** | NCT04099108 | South Africa | Effect of Combined IV Bolus Amino Acid Supplementation and Mobilisation on Muscle Mass in Patients Over the First Week of ICU Care: RCT | - | 80 | Combined cycle ergometry and bolus amino acid supplementation | Standard care only | Change in myofiber cross-sectional area over first week of ICU(muscle biopsy and ultrasound) | January 28, 2022 | Recruiting |
| **12** | NCT04261543 | Malaysia | The Effect of High Protein and Early Resistance Exercise Versus Usual Care in Critically Ill Patients (EFFORT-X Trial) | EFFORT-X | 120 | ≥2.2 g/kg boy weight through EN + Early cycle ergometry for 45 minutes/day | ≤1.2 g/kg body weight + usual care | Rectus femoris cross-sectional area and linear depth mass at Day10 of randomization (ultrasound) | May 16, 2023 | Recruiting |
| **13** | NCT05781971 | China | Effect of Nutritional Support and Early Rehabilitation on Sepsis | NUSPOTER | 948 | High protein: target protein amount of 2.0 g/kg/d (standard enteral nutrition is first choice, remaining target value will be met by intravenous amino acid infusion);  rehabilitation: early rehabilitation treatment such as respiratory training, bicycle training, and medium frequency electrical stimulation | 1. Active comparator - high protein alone: target protein amount of 2.0 g/kg/d (standard enteral nutrition is first choice, remaining target value will be met by intravenous amino acid infusion); 2. Placebo comparator - standard protein+early bedside rehabilitation: protein target of 1.2 g/kg/d supplied enterally or parenterally; early rehabilitation treatment such as respiratory training, bicycle training, and medium frequency electrical stimulation. 3. Sham comparator – standard protein: protein target of 1.2 g/kg/d supplied enterally or parenterally. | 28-day hospital mortality, 60-day quality of life | March 28, 2023 | Not yet recruiting |

## Table S3 Critical Care Nutrition Methodological Quality Scoring System

|  |  | | | | | |
| --- | --- | --- | --- | --- | --- | --- |
|  | **Score** | | | | | |
|  | **0** | | **1** | | **2** | |
| Randomization |  | | Not concealed or not sure |  | Concealed* randomization |  |
| Analysis | Other |  |  | | Intention to treat |  |
| Blinding | Not blinded |  | Single blinded  *Check who was blinded:*  Health Care Professionals  Outcomes Assessors |  | Double blinded |  |
| Patient selection | Selected patients or unable to tell |  | Consecutive eligible patients |  |  | |
| Comparability of groups at baseline | No or not sure |  | Yes |  |  | |
| Extent of follow-up | < 100% |  | 100% |  |  | |
| Treatment protocol | Poorly described |  | Reproducibly described |  |  | |
| Co-interventions** | Not described |  | Described but not equal or not sure |  | Well described and all equal |  |
| Outcomes | Not described |  | Partially described |  | Objectively defined |  |

**Total Score:** **(max 14)**

* Concealed randomization means the person enrolling the patients is unaware of the next treatment

assignment (e.g. phone in randomization, computer generated).

** Extent to which antibiotics, TPN, ventilation, oxygen, transfusions, etc were applied equally across groups

## Table S4: List of Excluded Studies, Reason for Exclusion and Study Source

|  | Abel RM, Beck CH Jr, Abbott WM, Ryan JA Jr, Barnett GO, Fischer JE. Improved survival from acute renal failure after treatment with intravenous essential L-amino acids and glucose. Results of a prospective, double-blind study. *N Engl J Med*. 1973;288(14):695-699. doi:10.1056/NEJM197304052881401 | Not high vs low protein | Search |
| --- | --- | --- | --- |
|  | Allingstrup MJ, Kondrup J, Wiis J, et al. Early goal-directed nutrition versus standard of care in adult intensive care patients: the single-centre, randomised, outcome assessor-blinded EAT-ICU trial. *Intensive Care Med.* 2017;43(11):1637-1647. doi:10.1007/s00134-017-4880-3 | Significant different in energy intake | Previous SR |
|  | Arabi YM, Al-Dorzi HM, Mehta S, et al. Association of protein intake with the outcomes of critically ill patients: a post hoc analysis of the PermiT trial. *Am J Clin Nutr.* 2018;108(5):988-996. doi:10.1093/ajcn/nqy189 | Post-hoc analysis of RCT | Search |
|  | ﻿Arefian NM, Teymourian H, Radpay B. Effect of partial parenteral versus enteral nutritional therapy on serum indices in multiple trauma patients. Tanaffos. 2007;6(4):37-41. http://ovidsp.ovid.com/ovidweb.cgi?T=JS&PAGE=reference&D=cctr&NEWS=N&AN=CN-00708370. | Significant different in energy intake; No clinically important outcomes | Search |
|  | Bauer P, Charpentier C, Bouchet C, Nace L, Raffy F, Gaconnet N. Parenteral with enteral nutrition in the critically ill. *Intensive Care Med*. 2000;26(7):893-900. doi:10.1007/s001340051278 | Significant different in energy intake | CCN website |
|  | **Badawy MM, Allam NM. Impact of Adding Protein Supplementation to Exercise Training on Lean Body Mass and Muscle Strength in Burn Patients. *J Burn Care Res*. 2021;42(5):968-974. doi:10.1093/jbcr/irab007** | **Not critically ill – intervention started after 6 weeks of burn** | **Search** |
|  | Berger MM, Pantet O, Jacquelin-Ravel N, et al. Supplemental parenteral nutrition improves immunity with unchanged carbohydrate and protein metabolism in critically ill patients: The SPN2 randomized tracer study. *Clin Nutr.* 2019;38(5):2408-2416. doi:10.1016/j.clnu.2018.10.023 | Significant different in energy intake | Search |
|  | Braunschweig CA, Sheean PM, Peterson SJ, et al. Intensive nutrition in acute lung injury: a clinical trial (INTACT). *JPEN J Parenter Enteral Nutr.* 2015;39(1):13-20. doi:10.1177/0148607114528541 | Significant different in energy intake | Previous SR |
|  | Braunschweig CL, Freels S, Sheean PM, et al. Role of timing and dose of energy received in patients with acute lung injury on mortality in the Intensive Nutrition in Acute Lung Injury Trial (INTACT): a post hoc analysis. *Am J Clin Nutr.* 2017;105(2):411-416. doi:10.3945/ajcn.116.140764 | Post-hoc analysis of RCT | Search |
|  | Brinson RR, Kolts BE. Diarrhea associated with severe hypoalbuminemia: a comparison of a peptide-based chemically defined diet and standard enteral alimentation. *Crit Care Med*. 1988;16(2):130-136. | Protein intake was not reported | Personal file |
|  | Casaer MP, Langouche L, Coudyzer W, et al. Impact of early parenteral nutrition on muscle and adipose tissue compartments during critical illness. *Crit Care Med*. 2013;41(10):2298-2309. doi:10.1097/CCM.0b013e31828cef02 | Significant different in energy intake | Previous SR |
|  | Casaer MP, Mesotten D, Hermans G, et al. Early versus late parenteral nutrition in critically ill adults. *N Engl J Med*. 2011;365(6):506-517. doi:10.1056/NEJMoa1102662 | Significant different in energy intake | Personal file |
|  | Clevenger FW, Gerding D, Steinle E, Rodriguez DJ, Osler TM. Effectiveness and tolerance to highly concentrated vs conventional TPN formulas. J Surg Res. 1993;55(2):228-232. doi:10.1006/jsre.1993.1134 | Significant different in energy intake | Search |
|  | Davies ML, Chapple LS, Chapman MJ, Moran JL, Peake SL. Protein delivery and clinical outcomes in the critically ill: a systematic review and meta-analysis. *Crit Care Resusc*. 2017;19(2):117-127. | Systematic review & meta-analysis – included studies reviewed | Search |
|  | Doig GS, Simpson F, Heighes PT, et al. Restricted versus continued standard caloric intake during the management of refeeding syndrome in critically ill adults: a randomised, parallel-group, multicentre, single-blind controlled trial. *Lancet Respir Med*. 2015;3(12):943-952. doi:10.1016/S2213-2600(15)00418-X | Significant different in energy intake | Previous SR |
|  | Doig GS, Simpson F, Sweetman EA, et al. Early parenteral nutrition in critically ill patients with short-term relative contraindications to early enteral nutrition: a randomized controlled trial. *JAMA*. 2013;309(20):2130-2138. doi:10.1001/jama.2013.5124 | Significant different in energy intake | Personal file |
|  | Eyer SD, Micon LT, Konstantinides FN, et al. Early enteral feeding does not attenuate metabolic response after blunt trauma. *J Trauma.* 1993;34(5):639-644. doi:10.1097/00005373-199305000-00005 | Significant different in energy intake | Previous SR |
|  | Fetterplace K, Gill BMT, Chapple LS, Presneill JJ, Macisaac C, Deane AM. Systematic Review With Meta-Analysis of Patient-Centered Outcomes, Comparing International Guideline – Recommended Enteral Protein Delivery With Usual Care. *JPEN J Parenter Enter Nutr*. 2020;44(4):610-620. doi:10.1002/jpen.1725 | Systematic review & meta-analysis – included studies reviewed | Search |
|  | Gillis C, Roque PS, Bläss J, et al. High dose amino acid administration achieves an anabolic response in type 2 diabetic patients that is independent of glycaemic control: A randomized clinical trial. *Clin Nutr*. 2018;37(4):1163-1171. doi:10.1016/j.clnu.2017.04.016 | Not critically ill | Search |
|  | Goeters C, Wenn A, Mertes N, et al. Parenteral L-alanyl-L-glutamine improves 6-month outcome in critically ill patients. *Crit Care Med.* 2002;30(9):2032-2037. doi:10.1097/00003246-200209000-00013 | Immunonutrition | Previous SR |
|  | Greig PD, Elwyn DH, Askanazi J, Kinney JM. Parenteral nutrition in septic patients: effect of increasing nitrogen intake. Am J Clin Nutr. 1987;46(6):1040-1047. doi:10.1093/ajcn/46.6.1040 | Not RCT | Previous SR |
|  | Grünert A, Diesch R, Kilian J, Dölp R. Untersuchungen zur parenteralen Applikation von Aminosäuren bei septischen patienten [Parenteral administration of amino acids to septic patients]. Anaesthesist. 1984;33(1):11-19. | Unable to find the full-text article | Search |
|  | **Hampton V, Hampton T, Dheansa B, Falder S, Emery P. Evaluation of high protein intake to improve clinical outcome and nutritional status for patients with burns: a systematic review. *Burns*. 2021;47(8):1714-1729. doi:10.1016/j.burns.2021.02.028** | **Systematic review & meta-analysis – included studies reviewed** | **Search** |
|  | Hausmann D, Mosebach KO, Caspari R, Rommelsheim K. Combined enteral-parenteral nutrition versus total parenteral nutrition in brain-injured patients. A comparative study. *Intensive Care Med*. 1985;11(2):80-84. doi:10.1007/BF00254779 | Significant different in energy intake | Search |
|  | Heidegger CP, Berger MM, Graf S, et al. Optimisation of energy provision with supplemental parenteral nutrition in critically ill patients: a randomised controlled clinical trial. *Lancet*. 2013;381(9864):385-393. doi:10.1016/S0140-6736(12)61351-8 | Significant different in energy intake | Search |
|  | Heimburger DC, Geels VJ, Bilbrey J, Redden DT, Keeney C. Effects of small-peptide and whole-protein enteral feedings on serum proteins and diarrhea in critically ill patients: a randomized trial. *JPEN J Parenter Enteral Nutr.* 1997;21(3):162-167. doi:10.1177/0148607197021003162 | No difference in energy and protein intake | Search |
|  | Heyland D, Muscedere J, Wischmeyer PE, et al. A randomized trial of glutamine and antioxidants in critically ill patients [published correction appears in N Engl J Med. 2013 May 9;368(19):1853. Dosage error in article text.]. *N Engl J Med.* 2013;368(16):1489-1497. doi:10.1056/NEJMoa1212722 | Immunonutrition | Previous SR |
|  | Hoffer LJ, Bistrian BR. Appropriate protein provision in critical illness: a systematic and narrative review. *Am J Clin Nutr*. 2012;96(3):591-600. doi:10.3945/ajcn.111.032078 | Systematic review & meta-analysis – included studies reviewed | Search |
|  | Hsieh LC, Chien SL, Huang MS, Tseng HF, Chang CK. Anti-inflammatory and anticatabolic effects of short-term beta-hydroxy-beta-methylbutyrate supplementation on chronic obstructive pulmonary disease patients in intensive care unit. Asia Pac J Clin Nutr. 2006;15(4):544-550. | Not high vs low protein | Previous SR |
|  | Hsu CW, Sun SF, Lin SL, et al. Duodenal versus gastric feeding in medical intensive care unit patients: a prospective, randomized, clinical study. *Crit Care Med.* 2009;37(6):1866-1872. doi:10.1097/CCM.0b013e31819ffcda | Significant different in energy intake | Previous SR |
|  | Huang HH, Chang SJ, Hsu CW, Chang TM, Kang SP, Liu MY. Severity of illness influences the efficacy of enteral feeding route on clinical outcomes in patients with critical illness. *J Acad Nutr Diet.* 2012;112(8):1138-1146. doi:10.1016/j.jand.2012.04.013 | Significant different in energy intake | Previous SR |
|  | Iapichino G, Radrizzani D, Scherini A, et al. Essential and non-essential amino acid requirement in injured patients receiving total parenteral nutrition. *Intensive Care Med*. 1988;14(4):399-405. doi:10.1007/BF00262896 | Not RCT | Previous SR |
|  | Ibrahim EH, Mehringer L, Prentice D, et al. Early versus late enteral feeding of mechanically ventilated patients: results of a clinical trial. JPEN J Parenter Enteral Nutr. 2002;26(3):174-181. doi:10.1177/0148607102026003174 | Significant different in energy intake | Previous SR |
|  | Ishibashi N, Plank LD, Sando K, Hill GL. Optimal protein requirements during the first 2 weeks after the onset of critical illness. Crit Care Med. 1998;26(9):1529-1535. doi:10.1097/00003246-199809000-00020 | Not RCT | Previous SR |
|  | Jensen GL, Miller RH, Talabiska DG, Fish J, Gianferante L. A double-blind, prospective, randomized study of glutamine-enriched compared with standard peptide-based feeding in critically ill patients. Am J Clin Nutr. 1996;64(4):615-621. doi:10.1093/ajcn/64.4.615 | Immunonutrition | Previous SR |
|  | Kagan I, Kremer S, Theilla M, Bendavid I, Singer P, Cohen J. OR63: Effect of Combined Protein Enriched Enteral Feeding and Early Cycle Ergometry in Mechanically Ventilated Critically Ill Patients: A Prospective, Randomized, Comparative, Single-Blind Controlled Study. *Clin Nutr.* 2017;36:S25-S26. doi:10.1016/S0261-5614(17)30724-0 | Abstract only | Search |
|  | Kearns PJ, Chin D, Mueller L, Wallace K, Jensen WA, Kirsch CM. The incidence of ventilator-associated pneumonia and success in nutrient delivery with gastric versus small intestinal feeding: a randomized clinical trial. *Crit Care Med*. 2000;28(6):1742-1746. doi:10.1097/00003246-200006000-00007 | Significant different in energy intake | Previous SR |
|  | Kerrie JP, Bagshaw SM, Brindley PG. Best evidence in critical care medicine. Early versus late parenteral nutrition in the adult ICU: feeding the patient or our conscience?. Can J Anaesth. 2012;59(5):494-498. doi:10.1007/s12630-012-9674-z | Commentary of EPANIC trial | Search |
|  | Kuhls DA, Rathmacher JA, Musngi MD, et al. Beta-hydroxy-beta-methylbutyrate supplementation in critically ill trauma patients. J Trauma. 2007;62(1):125-132. doi:10.1097/TA.0b013e31802dca93 | Not high vs low protein | Previous SR |
|  | Lambell KJ, King SJ, Forsyth AK, Tierney AC. Association of Energy and Protein Delivery on Skeletal Muscle Mass Changes in Critically Ill Adults: A Systematic Review. *JPEN* *J Parenter Enter Nutr*. 2018;42(7):1112-1122. doi:10.1002/jpen.1151 | Systematic review & meta-analysis – included studies reviewed | Search |
|  | Larsson J, Lennmarken C, Mårtensson J, Sandstedt S, Vinnars E. Nitrogen requirements in severely injured patients. *Br J Surg*. 1990;77(4):413-416. doi:10.1002/bjs.1800770418 | No clinically important outcome | Previous SR |
|  | Liebau F, Sundström M, van Loon LJ, Wernerman J, Rooyackers O. Short-term amino acid infusion improves protein balance in critically ill patients. *Critical Care.* 2015;19(1):106. doi:10.1186/s13054-015-0844-6. | Not RCT | CCN website |
|  | Long CL, Crosby F, Geiger JW, Kinney JM. Parenteral nutrition in the septic patient: nitrogen balance, limiting plasma amino acids, and calorie to nitrogen ratios. *Am J Clin Nutr.* 1976;29(4):380-391. doi:10.1093/ajcn/29.4.380 | Not RCT. No clinically important outcome | Previous SR |
|  | Ma N, Shen M, Wan Z, Pan S, Liu X, Yao Z. Zhonghua Wei Zhong Bing Ji Jiu Yi Xue. 2018;30(2):176-180. doi:10.3760/cma.j.issn.2095-4352.2018.02.016 | Significant different in energy intake; no different in protein | Search |
|  | Mansoor O, Breuillé D, Béchereau F, et al. Effect of an enteral diet supplemented with a specific blend of amino acid on plasma and muscle protein synthesis in ICU patients. *Clin Nutr.* 2007;26(1):30-40. doi:10.1016/j.clnu.2006.07.007 | Not high vs low protein | Previous SR |
|  | McKeever L, Peterson SJ, Lateef O, et al. Higher Caloric Exposure in Critically Ill Patients Transiently Accelerates Thyroid Hormone Activation. *J Clin Endocrinol Metab*. 2020;105(2):dgz077. doi:10.1210/clinem/dgz077 | Significant different in energy intake | Personal file |
|  | Meirelles CMJ, de Aguilar-Nascimento JE. Enteral or parenteral nutrition in traumatic brain injury: a prospective randomised trial. Nutr Hosp. 2011;26(5):1120-1124. doi:10.1590/S0212-16112011000500030 | Significant different in energy intake | Search |
|  | Meredith JW, Ditesheim JA, Zaloga GP. Visceral protein levels in trauma patients are greater with peptide diet than with intact protein diet. *J Trauma.* 1990;30(7):825-829. doi:10.1097/00005373-199007000-00011 | No difference in energy and protein intake | Personal file |
|  | Mesejo A, Montejo-González JC, Vaquerizo-Alonso C, et al. Diabetes-specific enteral nutrition formula in hyperglycemic, mechanically ventilated, critically ill patients: a prospective, open-label, blind-randomized, multicenter study. *Crit Care*. 2015;19:390. doi:10.1186/s13054-015-1108-1 | No difference in energy and protein intake | Search |
|  | Mowatt-Larssen CA, Brown RO, Wojtysiak SL, Kudsk KA. Comparison of tolerance and nutritional outcome between a peptide and a standard enteral formula in critically ill, hypoalbuminemic patients. *JPEN J Parenter Enteral Nutr.* 1992;16(1):20-24. doi:10.1177/014860719201600120 | No difference in energy and protein intake | Personal file |
|  | Nakamura K, Kihata A, Naraba H, et al. β-Hydroxy-β-methylbutyrate, Arginine, and Glutamine Complex on Muscle Volume Loss in Critically Ill Patients: A Randomized Control Trial. J*PEN J Parenter Enteral Nutr.* 2020;44(2):205-212. doi:10.1002/jpen.1607 | Immunonutrition | Personal file |
|  | **Nakanishi N, Matsushima S, Tatsuno J, et al. Impact of Energy and Protein Delivery to Critically Ill Patients: A Systematic Review and Meta-Analysis of Randomized Controlled Trials. *Nutrients*. 2022;14(22):4849. Published 2022 Nov 16. doi:10.3390/nu14224849** | **Systematic review & meta-analysis – included studies reviewed** | **Search** |
|  | National Heart, Lung, and Blood Institute Acute Respiratory Distress Syndrome (ARDS) Clinical Trials Network, Rice TW, Wheeler AP, et al. Initial trophic vs full enteral feeding in patients with acute lung injury: the EDEN randomized trial. *JAMA*. 2012;307(8):795-803. doi:10.1001/jama.2012.137 | Significant different in energy intake | Personal file |
|  | ﻿Ochoa J, Huhmann MB, Files DC, et al. Hypocaloric high-protein enteral nutrition improves glucose management in critically ill patients. *JPEN J Parenter Enter Nutr. 2017;41(2):289*. doi:http://dx.doi.org/10.1177/0148607116686023 (Abstract 30) | Abstract only; Significant different in energy intake | Search |
|  | Ott LG, Schmidt JJ, Young AB, et al. Comparison of administration of two standard intravenous amino acid formulas to severely brain-injured patients. Drug Intell Clin Pharm. 1988;22(10):763-768. doi:10.1177/106002808802201004 | No difference in energy and protein intake | Search |
|  | Ozgultekin A, Turan G, Durmus Y, et al. Comparison of the efficacy of parenteral glutamine and branched-chain amino acid solutions given as extra supplements in parallel to the enteral nutrition in head trauma. *E Spen Eur E J Clin Nutr Metab* 2008; 3: e211-6. | Immunonutrition | Previous SR |
|  | ﻿Pertikov SS, Solodov AA, Tveritnev PM, et al. Use A High-Protein Tube Feeding In Critically Ill Patients: The Results Of A Multicenter Study. *Clin Nutr.* 2019;38(Supplement 1):S300. doi:10.1016/S0261-5614%2819%2932614-7 | Abstract only | Search |
|  | Petros S, Horbach M, Seidel F, Weidhase L. Hypocaloric vs Normocaloric Nutrition in Critically Ill Patients: A Prospective Randomized Pilot Trial. *JPEN J Parenter Enteral Nutr.* 2016;40(2):242-249. doi:10.1177/0148607114528980 | Significant different in energy intake | Personal file |
|  | Pitkänen O, Takala J, Pöyhönen M, Kari A. Nitrogen and energy balance in septic and injured intensive care patients: response to parenteral nutrition. *Clin Nutr.* 1991;10(5):258-265. doi:10.1016/0261-5614(91)90004-v | No clinically important outcome | CCN websie |
|  | Qiu C, Chen C, Zhang W, et al. Fat-Modified Enteral Formula Improves Feeding Tolerance in Critically Ill Patients: A Multicenter, Single-Blind, Randomized Controlled Trial. *JPEN J Parenter Enteral Nutr.* 2017;41(5):785-795. doi:10.1177/0148607115601858 | Significant different in energy intake | Previous SR |
|  | ﻿Rice TW, Files DC, Morris P, et al. Facilitated glucose control in critically Ill patients utilizing a very high protein low carbohydrate formula. *Intensive Care Med Exp.* 2017;5(2):269. doi:10.1186/s40635-017-0151-4 | Abstract only; Similar protein between group | Search |
|  | Rice TW, Mogan S, Hays MA, Bernard GR, Jensen GL, Wheeler AP. Randomized trial of initial trophic versus full-energy enteral nutrition in mechanically ventilated patients with acute respiratory failure. *Crit Care Med.* 2011;39(5):967-974. doi:10.1097/CCM.0b013e31820a905a | Significant different in energy intake | Previous SR |
|  | Ridley EJ, Davies AR, Parke R, et al. Supplemental parenteral nutrition versus usual care in critically ill adults: a pilot randomized controlled study. *Crit Care*. 2018;22(1):12. doi:10.1186/s13054-018-1939-7 | Significant different in energy intake | Personal file |
|  | Saffle JR, Larson CM, Sullivan J. A randomized trial of indirect calorimetry-based feedings in thermal injury. *J Trauma*. 1990;30(7):776-783. doi:10.1097/00005373-199007000-00003 | No clinically important outcomes reported by protein group | Personal file |
|  | Scheinkestel CD, Kar L, Marshall K, Bailey M, Davies A, Nyulasi I, Tuxen DV. Prospective randomized trial to assess caloric and protein needs of critically Ill, anuric, ventilated patients requiring continuous renal replacement therapy. *Nutrition*. 2003 Nov-Dec;19(11-12):909-16. | Not high vs low protein | Search |
|  | Schmitz JE, Lotz P, Ahnefeld FW, Grünert A. Untersuchungen zur Eiweiss- und Energieversorgung von Intensivpatienten [Protein and energy metabolism in intensive care patients]. *Infusionsther Klin Ernahr*. 1981;8(4):158-162. | Unable to find the full-text article; No clinically important outcome | Search |
|  | ﻿Seres DS, Ippolito PR. Pilot study evaluating the efficacy, tolerance and safety of a peptide-based enteral formula versus a high protein enteral formula in multiple ICU settings (medical, surgical, cardiothoracic). Clin Nutr. 2017;36(3):706‐709. doi:10.1016/j.clnu.2016.04.016 | No clinically important outcome per group; Nutrition intake per group not reported | Search |
|  | Serra MC, Verceles AC, Parker EA, Feinberg T. Effects of protein supplementation on energy and protein intake: Preliminary findings from a randomized, controlled trial in the intensive care unit. *Glob Adv Heal Med.* 2018;7:118. doi:10.1177/2164956118773837 (Abstract 2251) | Abstract only: Significant different in energy intake; No clinically important outcomes | Search |
|  | Shaw JH, Wildbore M, Wolfe RR. Whole body protein kinetics in severely septic patients. The response to glucose infusion and total parenteral nutrition. *Ann Surg*. 1987;205(3):288-294. doi:10.1097/00000658-198703000-00012 | Not RCT | Previous SR |
|  | Singer P, Anbar R, Cohen J, et al. The tight calorie control study (TICACOS): a prospective, randomized, controlled pilot study of nutritional support in critically ill patients. *Intensive Care Med*. 2011;37(4):601-609. doi:10.1007/s00134-011-2146-z | Significant different in energy intake | Search |
|  | Singer P, De Waele E, Sanchez C, et al. TICACOS international: A multi-center, randomized, prospective controlled study comparing tight calorie control versus Liberal calorie administration study. *Clin Nutr*. 2021;40(2):380-387. doi:10.1016/j.clnu.2020.05.024 | Significant different in energy intake | Search |
|  | Taylor SJ, Fettes SB, Jewkes C, Nelson RJ. Prospective, randomized, controlled trial to determine the effect of early enhanced enteral nutrition on clinical outcome in mechanically ventilated patients suffering head injury. *Crit Care Med.* 1999;27(11):2525-2531. doi:10.1097/00003246-199911000-00033 | Significant different in energy intake | Search |
|  | Tiengou LE, Gloro R, Pouzoulet J, et al. Semi-elemental formula or polymeric formula: is there a better choice for enteral nutrition in acute pancreatitis? Randomized comparative study. *JPEN J Parenter Enteral Nutr.* 2006;30(1):1-5. doi:10.1177/014860710603000101 | No difference in energy and protein intake | Personal file |
|  | Twyman D, Young AB, Ott L, Norton JA, Bivins BA. High protein enteral feedings: a means of achieving positive nitrogen balance in head injured patients.. *JPEN J Parenter Enteral Nutr.* 1985 Nov-Dec;9(6):679-84. | No clinically important outcomes | Search |
|  | van der Heijden A, Verbeek MJ, Schreurs VV, Akkermans LM, Vos A. Efecto del aumento de la ingesta de proteínas sobre el balance nitrogenado en pacientes críticos ventilados mecánicamente y sometidos a nutrición parenteral total [Effect of increasing protein ingestion on the nitrogen balance of mechanically ventilated critically ill patients receiving total parenteral nutrition]. *Nutr Hosp.* 1993;8(5):279-287. | Abstract only; No clinically important outcomes | Search |
|  | Verbruggen SC, Coss-Bu J, Wu M, Schierbeek H, Joosten KF, Dhar A, et al. Current recommended parenteral protein intakes do not support protein synthesis in critically ill septic, insulin-resistant adolescents with tight glucose control. *Crit Care Med* 2011;39(11):2518-25. | Adolescent | CCN website |
|  | Wandrag L, Brett SJ, Frost G, Hickson M. Impact of supplementation with amino acids or their metabolites on muscle wasting in patients with critical illness or other muscle wasting illness: a systematic review. *J Hum Nutr Diet*. 2015;28(4):313-330. doi:10.1111/jhn.12238 | Systematic review & meta-analysis – included studies reviewed | Search |
|  | Wichansawakun S, Wongkongkathep P, & Tantiyavarong, P. Clin Nutr 2019.. MON-PO626: A Randomized Controlled Trial of the Effect of Protein Restriction to Delay Renal Replacement Therapy in Septic Patients with Acute Renal Failure in Thammasat University Hospital, Preliminary Analysis. doi:﻿10.1016/S0261-5614%2819%2932459-8 | Abstract only | Search |
|  | Wischmeyer PE, Hasselmann M, Kummerlen C, et al. A randomized trial of supplemental parenteral nutrition in underweight and overweight critically ill patients: the TOP-UP pilot trial. *Crit Care*. 2017;21(1):142. doi:10.1186/s13054-017-1736-8 | Significant different in energy intake | Search |
|  | Wolfe RR, Goodenough RD, Burke JF, Wolfe MH. Response of protein and urea kinetics in burn patients to different levels of protein intake. *Ann Surg.* 1983;197(2):163-171. doi:10.1097/00000658-198302000-00007 | No clinically important outcomes | Previous SR |
|  | **Zhou W, Yu L, Fan Y, et al. Effect of early mobilization combined with early nutrition on acquired weakness in critically ill patients (EMAS): A dual-center, randomized controlled trial. PLoS One. 2022;17(5):e0268599. Published 2022 May 26. doi:10.1371/journal.pone.0268599** | **Not high vs low protein (author also cannot provide the nutrition delivered)** | **Search** |

**Note: Bold words are the new excluded studies from the current updated work**

CCN: critical care nutrition, RCT: randomized controlled trial, SR: systematic review

## Table S5: Patients’ Baseline Characteristics of Included Studies

| **Author, year (country)** | **Age** | | **Sex (M/F)** | | **APACHE II** | | **SOFA** | | **MV,%** | | **Medical,%** | | **Sepsis.%** | | **Weight, kg or BMI, kg/m^2^** | | **Patients with AKI or baseline kidney dysfunction or risk of AKI progression^$^** | |
| --- | --- | --- | --- | --- | --- | --- | --- | --- | --- | --- | --- | --- | --- | --- | --- | --- | --- | --- |
|  | **Higher** | **Lower** | **Higher** | **Lower** | **Higher** | **Lower** | **Higher** | **Lower** | **Higher** | **Lower** | **Higher** | **Lower** | **Higher** | **Lower** | **Higher** | **Lower** | **Higher** | **Lower** |
| Clifton 1985^9^  (USA) | 34±12 | 38±12 | 9/1 | 10/0 | - | - | - | - | - | - | - | - | - | - | - | - | - | - |
| Saffle 1990#^3^  (USA) | 32.8±2.7 | 32.7±3.0 | - | - | - | - | - | - | - | - | - | - | - | - | - | - | - | - |
| Mesejo 2003^10^  (Spain) | 64.6±9.63 | 65.2 ±14.95 | 17/7 | 24/2 | 18 (14-21) | 19 (16-20) | - | - | - | - | - | - | - | - |  |  | - | - |
| Zhou 2006^11^  (China) | 65.7±15.6 | | 29/22 | | APACHE II>19: 31/51 (60.8) | | - | - | - | - | - | - | - | - | - | - | - | - |
| Singer 2007^12^  (Israel) | 74±6 | 55±20 | - | - | SAPS II: 13.6±3.4 | SAPS II: 12.8±3.3 | - | - | 100 | 100 | - | - | 12.5 | 16.7 | - | - | AKI: 8/8 (100%) | AKI: 6/6 (100%) |
| Rugeles 2013^13^ (Columbia) | 53.3 ±19.5 | 55.7 ±19.5 | 22/18 | 24/16 | 13.9±4.8 | 15.1 ±6.2 | 7.5±2.9 | 6.7±2.5 | - | - | 100 | 100 | - | - | 63±10.7 | 65.8±11.0 | 0 | 0 |
| Doig 2015^6^ (Australia) | 63.3 ±15.4 | 62.7 ±16.6 | 158/81 | 147/88 | 21.7 ±7.6 | 20.2 ±6.8 | - | - | 82.4 | 82.5 | 66.5 | 66.4 | 14.6 | 10.6 | BMI:  28.9 ± 7.0 | BMI:  29.5 ± 6.9 | Baseline kidney dysfunction or risk of AKI progression: 60/239 (25.1%) | Baseline kidney dysfunction or risk of AKI progression: 46/235 (19.6%) |
| Ferrie 2015^14^  (Australia) | 67.0 (55.5-74.3) | 64.5 (49.3-70.0) | 38/22 | 36/24 | 25.5 ±9.4 | 23.7 ±8.1 | 9.4 ±4.1 | 9.2 ±4.0 | 95 | 98 | - | - | - | - | 73.2 ±16.1 | 77.7 ±21.7 | CRRT: 9/59 (15.3%) | CRRT: 11/60 (18.3%) |
| Jakob 2017^15^  (Switzerland) | 65.3 (52.6-75.3) | 61.6 (48.6-71.3) | 33/13 | 28/16 | 28.5 (22.3-32.8) | 27.5 (22.0-33.3) | 8.0 (6.0-11.0) | 7.0 (5.0-10.0) | 94 | 98 | - | - | - | - | BMI: 28.8 (25.1-34.2) | BMI: 27.8 (23.5-31.5) | - | - |
| Fetterplace 2018^16^  (Australia) | 55±13 | 57±16 | 23/7 | 21/9 | 22±6.2 | 20 ±5.9 | - | - | 100 | 100 | 70 | 53 | 10 | 3.3 | BMI: 30 ±7.1 | BMI: 29 ±5.3 | - | - |
| Van Zanten 2018^17^ (Netherlands) | 63.9 ±13.3 | 60.8 ±15.2 | 9/13 | 13/9 | 25 (21-28) | 24 (18-27) | 10 (9-11) | 9 (7-11) | 100 | 100 | 36.4 | 40.9 | - | - | 84.9 ±18.3  BMI: 30.3 ±4.1 | 91.2 ±20.7  BMI: 30.7 ±8.4 | - | - |
| Vega-Alava 2018^18^ (Philippines) | 57 (47-70) | 59 (45-72) | 11/9 | 9/11 | SAPS II: 23.5 (19-34) | SAPS II: 25.7 (19-36) | - | - | 100 | 100 | - | - | - | - | BMI: 22.23 (19.6-25.7) | BMI: 23.21 (18.8-26.1) | - | - |
| Azevedo 2019^19^ (Brazil) | 65.0 ±18.8 | 67.4 ±18.9 | 34/23 | 32/31 | APACHE IV: 81.1 ±32.4 | APACHE IV: 77.2 ±30.7 | 9.8 ±14.6 | 6.8 ±4.0 | 100 | 100 | 80 | 73 | 21.0 | 23.8 | - | - | - | - |
| Danielis 2019^20^  (Italy) | 66 (57-72) | 63 (46-70) | 11/8 | 11/10 | 17 (13.5-22) | 17 (13-22) | - | - | 100 | 100 | - | - | - | - | BMI: 25 (23-27.5) | BMI: 24 (21-26) | 0 | 0 |
| Badjatia 2020^21^ (USA) | 60±8 | 58±14 | 5/7 | 5/8 | 20±5 | 18±10 | - | - | - | - | - | - | - | - | - | - | - | - |
| Bukhari 2020^^2^  (Indonesia) | TBI: 38.29 ±18.35  Non-TBI: 50.25 ±15.92 | TBI: 41.6 ±20.11  Non-TBI: 42.44 ±20.27 | - | - | - | - | - | - | - | - | - | - | - | - | IBW  TBI: 59.27 ±6.87  Non-TBI: 52.48 ±5.44 | IBW  TBI: 56.20 ±3.90  Non-TBI: 55.26 ±6.26 | - | - |
| Chapple 2020^22^ (Australia) | 60 (50-72) | 61 (46-68) | 39/19 | 44/14 | 22 (16-26) | 22 (16-27) | - | - | 100 | 100 | 81 | 71 | - | - | ABW: 85 (75-100)  IBW: 65 (57-71) | ABW: 85 (75-101)  IBW: 67 (57-72) | RRT: 4/58 (6.9%) | RRT: 2/58 (3.4%) |
| Nakamura 2020^23^ (Japan) | 68.3 ±14.3 | 67.9 ±14.9 | 35/25 | 38/19 | 18.6 ±8.1 | 18.2 ±6.0 | 6.8±3.1 | 7.1±3.5 | 85.0 | 91.2 | - | - | 45.0 | 52.6 | 53.1 ±9.6  BMI: 21.3 ±3.9 | 53.6 ±11.2  BMI: 21.5 ±4.5 | RRT: 9/60 (15.0%) | 8/57 (14.0%) |
| Azevedo 2021^24^  (Brazil) | 67.6±17.8 | 65.3±19.7 | 53/34 | 46/48 | - | - | 5 (3-9) | 6 (3.7-8) | 100 | 100 | 73 | 74 | - | - | - | - | - | - |
| Carteron 2021^25^ (France) | 57 (44-65) | 55 (40-65) | 67/33 | 53/42 | SAPS II: 48±12 | SAPS II: 49±13 | - | - | 100 | 100 | - | - | - | - | IBW: 65 (58-70)  BMI: 26 (23-29) | IBW:  64 (56-70)  BMI: 26 (23-29) | - | - |
| Dresen 2021^26^ (Germany) | 66±16 | 64±15 | 15/6 | 15/6 | SAPS II: 46 ±12 | SAPS II: 45 ±10 | 6±3 | 8±3 | 100 | 100 | 0 | 0 | 90 | 81 | IBW @ BMI 25: 75 ±8.2 | IBW @ BMI 25: 78 ±9.6 | - | - |
| Kagan 2022*^27^  (Israel) | 61±16 | 63±18 | 14/5 | 12/9 | 22±8 | 20±6 | 7±4 | 5±2 | 100 | 100 | - | - | 21.1 | 14.3 | BMI: 30±7 | BMI: 30±7 | - | - |
| Heyland 2023^28^  (Canada) | 57 (17 [18–95]) | 57 (17 [18–93]) | 395/250 | 388/267 | 609/645 (21 [16–27]) | 621/656 (21 [15–26]) | 9 (6–11) | 9 (6–11) | 100 | 100 | 85 | 82 | 13 | 14 | BMI 28 (10 [13–85]) | BMI 28·6 (9 [13–77]) | AKI: 163/645 (25.3%) | AKI: 149/656 (22.7%) |

Data are presented as Mean±SD or Median (Q1-Q3)

# Saffle 1990 randomized patients to indirect calorimetry vs predictive equation, then further randomized them to high vs low protein formula. The age is reported in 4 groups in Table 2. They are combined by higher versus lower protein group using an online calculator: <https://home.ubalt.edu/ntsbarsh/business-stat/otherapplets/Pooled.htm>.

^Bukhari 2020 has 3 groups: control (n=22), high-protein polymeric (n=19) and oligomeric group (n=14), the control group was excluded from the analysis.

ABW: actual body weight, AKI: acute kidney injury, APACHE II: acute physiology and chronic health evaluation II, BMI: body mass index, CRRT: continuous renal replacement therapy, F: female, IBW: ideal body weight, M: male, MV: mechanical ventilation, RRT: renal replacement theraepy, SAPS II: simplified acute physiology score II, SOFA: sequential organ failure assessment, TBI: traumatic brain injury

*Kagan 2022 has 3 groups: Group 1 (control, n=22), Group 2 (low protein + cycle, n=21), and Gorup 3 (high protein + cycle, n=19). Group 2 was excluded from the analysis.

^$^Definition of AKI, baseline kidney dysfunction or risk of progression of AKI:

- **Singer 2007**: Acute renal failure was defined as a 50% decrease in GFR, a doubling of serum creatinine or an increase of creatinine to 3.5 mg/dL (309.4 umol/L).
- **Doig 2015 (**mortality of paitents with kidney dysfunction or risk of progression of AKI from Doig 2015 is 90-d mortality from their secondary publication ^29^)
  - Baseline kidney dysfunction: Creatinine at time of enrolment > 168μmol/L (by Gordon Bernard’s *“*Brussels Table”)
  - Risk of progression of AKI at enrollment: was defined as a rise in creatinine over the previous 24 hours by at least 20% to over 120 μmol/L
- **Heyland 2023**: Acute kidney injury refers to patients who met the criteria of KDIGO:
  - stage 1 is at least 26·52 μmol/L increase in serum creatinine from baseline within 48 h or 1·5–1·9 times baseline within 7 days
  - stage 2 is 2·0–2·9 times baseline within 7 days
  - stage 3 is three times or more baseline within 7 days or increase to at least 353·6 μmol/L with an acute increase of more than 44·2 μmol/L.

## Table S6: Nutritional Prescription, Prescription body weight and Energy and Protein Delivered

| **Author, year (country)** | **Nutritional Prescription** | | **Prescription body weight** | | **Energy Delivered** | | **Protein Delivered** | |
| --- | --- | --- | --- | --- | --- | --- | --- | --- |
|  | **High** | **Low** | **High** | **Low** | **High** | **Low** | **High** | **Low** |
| Clifton 1985  (USA) | **E**: Repeated IC and provide 150% of resting metabolic expenditure  **P**: EN formula with 83g protein/litre; 22% calories from protein | **E**: Repeated IC and provide 150% of resting metabolic expenditure  **P**: EN formula with 70g protein/litre; 14% calories from protein | - | | 52±11 kcal/kg/d  141±15% of resting metabolic expenditure | 48±8 kcal/kg/d  139±9% of resting metabolic expenditure | 29.0±5.3 g N_2_/d (~181.3±33.1 g/d)  0.42±0.09 g N_2_/kg/d (~2.63±0.56 g/kg) | 17.6±3.6 g N_2_/d (~110±22.5 g/d)  0.24±0.04 g N_2_/kg/d  (~1.50±0.25 g/kg) |
| Saffle 1990#  (USA) | **E:** 1) Curreri formula; 2) indirect calorimetry*1.2  **P:** EN formula with 22.4% of calories from protein | **E:** 1) Curreri formula; 2) indirect calorimetry*1.2  **P:** EN formula with 16.7% of calories from protein | Patients weighed daily on a bedside scale (values not reported) | | CURR:  43.4±2.1 kcal/kg/d;  0.94±0.04% of Curreri;  1.43±0.14% of IC  IC:  47.2±3.3 kcal/kg/d;  0.93±0.06% of Curreri;  1.25±0.06% of IC  Pooled: 45.53±2.84 (25) | CURR:  43.7±1.7 kcal/kg/d;  0.93±0.04% of Curreri;  1.44±0.08% of IC  IC:  43.6±2.3 kcal/kg/d;  0.85±0.06% of Curreri;  1.17±0.05% of IC  Pooled: 43.65±2.02 (24) | CURR:  148.5±10.4 gm/d;  1.93±0.16 gm/kg/d  IC:  156.3±9.9 gm/d;  2.03±0.14 gm/kg/d  Pooled: 1.99±0.15 (25) | CURR:  118.1±8.1 gm/d;  1.44±0.08 gm/kg/d  IC:  114.0±1.1 gm/d;  1.45±0.14 gm/kg/d  Pooled: 1.45±0.11 (24) |
| Mesejo 2003  (Spain) | **E**: Harris-benedict equation*1.2  **P:** EN Formula with 22% of calories from protein | **E**: Harris-benedict equation*1.2  **P:** EN Formula with 20% of calories from protein | IBW (method of calculation not reported) | | 1664±203 kcal/d | 1599±226 kcal/d | 14±2.48 N_2_/d (88.8±15.5 g/d) | 12.8±1.8 N_2_/d (80.0±11.3 g/d) |
| Zhou 2006  (China) | **E:** 25 kcal/kg  **P:** EN formula with NPC:N = 100:1 (Fresubin 750 MC) | **E:** 25 kcal/kg  **P:** EN formula with NPC:N = 130:1 (Nutrison Fibre) | Ideal weight, kg = height in cm - 105 | | - | - | - | - |
| Singer 2007  (Sepsis) | **E:** 2000 kcal/day non-protein calories (dextrose and Intralipid)  **P:** 150 g amino acids (Aminoplasmal 10%, B Braun, Germany) | **E:** 2000 kcal/day non-protein calories (dextrose and Intralipid)  **P:** 75 g amino acids (normal-dose amino acids) | - | | - | - | - | - |
| Rugeles 2013 (Columbia) | **E**: 15 kcal/kg/d  **P**: >1.5 g/kg/d | **E**: 25 kcal/kg/d  **P**: Usual care | - | | 12±0.5 kcal/kg/d^£^ | 14±0.5 kcal/kg/d^£^ | 1.4±0.1 g/kg/d^£^ | 0.76±0.2 g/kg/d^£^ |
| Doig 2015 (Australia) | **E**: -  **P**: up to 2.0 g/kg/d | Decide by the attending clinicians | If BMI>25, use IBW at BM 23 | Decide by the attending clinicians | eFig1c  ~1200 kcal/d | eFig1c  ~956 kcal/d | eFig1a ~1.67 g/kg/d | eFig1a ~0.71 g/kg/d |
| Ferrie 2015  (Australia) | **E**: 25 kcal/kg/d  **P**: PN formula with 57g amino acids/litre; 21.5% calories from amino acids | **E**: 25 kcal/kg/d  **P**: PN formula with 40g amino acids/litre; 13.3% calories from amino acids | Current weight if BMI 20-30; Weight at BMI 20 if underweight; Weight at BMI 27.5 if obese | | First 3 study days:  1053±450 kcal/d  23.5±3.9 kcal/kg/d  First 7 study days:  1610±468 kcal/d  23.1±3.9 kcal/kg/d | First 3 study days:  1700±524 kcal/d  26.0±3.8 kcal/kg/d  First 7 study days:  1720±516 kcal/d  24.9±4.2 kcal/kg/d | First 3 study days:  76±25 g/d  1.17±0.21 g/kg/d  First 7 study days:  76±26 g/d  1.09± 0.22 g/kg/d | First 3 study days:  55±20 g/d  0.87±1.17g/kg/d  First 7 study days:  60±21 g/d  0.90± 0.21 g/kg/d |
| Jakob 2017  (Switzerland) | **E**: 25 kcal/kg/d and adjusted by IC during the first night after study start, 3 days afterwards, at end of nutrition with the study product, and 2 days afterwards if still intubated  **P**: EN formula with 94 g protein/litre; 25% calories from protein | **E**: 25 kcal/kg/d and adjusted by IC during the first night after study start, 3 days afterwards, at end of nutrition with the study product, and 2 days afterwards if still intubated  **P**: EN formula with 61 g protein/litre; 16% calories from protein | Weight taken from medical records or relatives or estimated by medical staff. (not clear ABW or IBW) | | 18 (12.5-20.9) kcal/kg  14.4±6.6 kcal/kg^£^  85 (71-95) % | 19.7 (17.3-23.1 kcal/kg)  17.2±6.4 kcal/kg^£^  90 (84-96)% | 1.13 (0.78-1.31) g/kg  0.9±0.4 g/kg^£^ | 0.80 (0.70-0.94) g/kg  0.7±0.2 g/kg^£^ |
| Fetterplace 2018  (Australia) | **E**: 25 kcal/kg/d  **P**: 1.5 g/kg/d  (Volume-based feeding protocol) | **E**: 25 kcal/kg/d  **P**: 1.0 g/kg/d | Age<65:  - use ABW if BMI 18.5-25  Age≥65:  - use ABW if BMI 22-27  If BMI≥32, use adjusted weight = ideal weight + 25% (actual weight – ideal weight) | | 1646±447 kcal/d  21±5.2 kcal/kg/d  Include non-nutrition E  1835±340 kcal/d  23±5.7 kcal/kg/d  84±21% | 1398±308 kcal/d  18±2.7 kcal/kg/d  Include non-nutrition E  1598±340 kcal/d  21±3.3 kcal/kg/d  73±11% | 94±27 g/d  1.2±0.3 g/kg/d  90±25% | 58±12 g/d  0.75±0.11g/kg/d  57±8% |
| van Zanten 2018 (Netherlands) | **E**: 25 kcal/kg IBW/d  **P**: EN formula with 8 g protein/100 kcal; 32% of calories from protein | **E**: 25 kcal/kg IBW/d  **P**: EN formula with 5 g protein/100 kcal; 20% of calories from protein | IBW at BMI 30 (if BMI>30). | | 1162±606 kcal/d (first 10d)  15.0±9.3 kcal/kg/d (first 10d)^£^  16.6 (8.9-23.3) kcal/kg IBW/d (28 days) | 1163±375 kcal/d (first 10d)  13.8±8.6 kcal/kg/d (first 10d)^£^  14.4 (10.9-18.8) kcal/kg IBW/d (28 days) | 1.37±0.82 g/kg IBW/d (day 5)  1.20±0.74 g/kg IBW/d (first 10d) ^£^ | 0.72±0.47 g/kg IBW/d (day 5)  0.70±0.43 g/kg IBW/d (first 10d) ^£^ |
| Vega-Alava 2018 (Philippines) | **E:** 25-30 kcal/kg IBW/d * stress factor  **P:** EN formula with hydrolyzed whey protein (10 g protein per serving and 16% of calories from protein) + 100% whey protein supplement (6 g protein per serving), 3 servings, every 8 hour | **E:** 25-30 kcal/kg IBW/d * stress factor  **P:** EN formula with hydrolyzed whey protein (10 g protein per serving and 16% of calories from protein) only | IBW computed based on height | | - | - | - | - |
| Azevedo 2019 (Brazil) | **E**: IC daily for the first 3 days, then IC every 2 days until day 10  **P**: 2.0-2.2 g/kg/d | **E**: 25 kcal/kg/d  **P**: 1.4-1.5 g/kg/d | - | | 1139 (890-1278) kcal/d  73.2% of IC | 1140 (889-1331) kcal/d  78% of 25 kcal/kg/d | 1.69 (1.33-1.80) g/kg/d  80% of 2.1 g/kg/d | 1.13 (0.97-1.34) g/kg/d  77.9% of 1.45 g/kg/d |
| Danielis 2019  (Italy) | **E:** *Age<60:* 8 x weight (kg) + 14 x height (cm) + 32 x minute ventilation (L/min) + 94 x temperature (degrees °C) – 4834; *Age >60:* (0.85 x Harris Benedict equation) + (175 x temperature degrees °C) + (33 x minute ventilation (L/min)) – 6433.  **P:** 1.8 g/kg/d  (*EN:* Protein 21%, Carbs/Dextrose 31%, Fats 46% Fiber 2%; *PN:* Protein 22%, Carbs/Dextrose 40%, Fats 38%) | **E:** 20-25 kcal/kg/d  **P:** follow energy and type of formula  (*EN:* Protein 16%, Carbs/Dextrose 35%, Fats 49%; *PN:* Protein 16%, Carbs/Dextrose 49%, Fats 35%) | Weight assessed by ICU bed scale | | 1490±292.8 kcal/d^£^ | 1460±403.3 kcal/d^£^ | 100±17.6 g/d^£^ | 52±18.1 g/d^£^ |
| Badjatia 2020 (USA) | **E**: Mifflin St-Jeor (non-intubated); Penn State (intubated)  **P**: 1.75 g/kg/d (≥9g leucine/d) | **E**: Mifflin St-Jeor (non-intubated); Penn State (intubated)  **P**: 1.2-1.4 g/kg/d | - | | 20.0±7.1 kcal/kg/d | 19.8±9.9 kcal/kg/d | 1.51±0.47 g/kg/d | 0.88±0.36 g/kg/d |
| Bukhari 2020^  (Indonesia) | **E**: 25-30 kcal/kg/d  **P**: 1.2-2.0 g/kg/d  **EN Formula:** High-protein polymeric (22.4% calories from protein) | **E**: 25-30 kcal/kg/d  **P**: 1.2-2.0 g/kg/d  **EN Formula:** Oligomeric (16.2% calories from protein) | IBW (method of calculation not reported) | | TBI: 2358.65±714.57 kcal/d  Non TBI: 1998.96 ±684.16 kcal/d  Pooled: 2131.48 ±695.04 kcal/d | TBI: 2336.94 ±891.93 kcal/d  Non TBI: 1887.12 ±598.72 kcal/d  Pooled: 2015.64 ±683.91 kcal/d | TBI: 134.03 ±42.58 g/d  Non-TBI: 102.88 ±37.47 g/d  Pooled: 114.36±39.35 g/d | TBI: 93.79±26.65 g/d  Non-TBI: 80.29±29.38 g/d  Pooled: 84.15±28.72 g/d |
| Chapple 2020 (Australia) | **Goal Volume:** 1mg/kg IBW/h (max 100 ml/h)  **E:** EN formula with 1260 kcal/liter  **P**: EN formula with 100 g protein/litre; 32% of calories from protein | **Goal Volume:** 1mg/kg IBW/h (max 100 ml/h)  **E:** EN formula with 1250 kcal/liter  **P**: EN formula with 63 g protein/litre; 20% of calories from protein | IBW - Men = (height (cm) – 152.4) * 0.9 + 50  - Women = (height (cm) – 152.4) * 0.9 + 45.5 | | Trial EN:  19.2±6.5 kcal/kg IBW/d  1233±487 kcal/d  Trial EN + PN:  1245±489 kcal/d | Trial EN:  19.6±5.4 kcal/kg IBW/d  1260±393 kcal/d  Trial EN + PN:  1271±392 kcal/d | Trial EN+PN, albumin, protein supplements:  1.52±0.52 g/kg IBW/d | Trial EN+PN, albumin, protein supplements:  0.99±0.27 g/kg IBW/d |
| Nakamura 2020 (Japan) | **E**: 20 kcal/kg/d (initial target), up to 30 kcal/kg/d if malnutrition (defined as BMI<18.5, recent weight loss>10%, or physician’s decision)  **P**: 1.8 g/kg/d | **E**: 20 kcal/kg/d (initial target), up to 30 kcal/kg/d if malnutrition (defined as BMI<18.5, recent weight loss>10%, or physician’s decision)  **P**: 0.9 g/kg/d | - | | 17.9±10.1 kcal/kg/d^£^ | 16.2±9.5 kcal/kg/d^£^ | 1.36±0.80 g/kg/d^£^ | 0.72±0.46 g/kg/d^£^ |
| Azevedo 2021  (Brazil) | **E:** 50-70% of MEE on days 3 and 4, increase to 80% of MEE on day 5-6  **P:** 0.8-1.0 g/kg/d on days 3 and 4, increase to 2.0-2.2 g/kg/d on day 5-6, initiation of PN if protein target not met on day 7 | **E:** 50-70% of MEE on days 3 and 4, increase to 80% of MEE on day 5-6  **P:** 0.8-1.0 g/kg/d on days 3 and 4, increase to 1.4-1.5 g/kg/d on day 5-6, initiation of PN if protein target not met on day 7 | - | | Calories (% of MEE): 81 (74.4-86.2);  Calories D3: 13.7 (11.3-17.0) kcal/kg/d;  Calories D7: 19.5 (16.0-22.0) kcal/kg/d | Calories (% of MEE):  81.7 (74.0-90.2);  Calories D3: 15.0 (12.0-18.0) kcal/kg/d  Calories D7: 19.0 (14.3-21.4) kcal/kg/d | Total protein:  1.48 (1.25-1.64) g/kg/d  Protein D3: 1.23 (0.85-1.60) g/kg/d  Protein D7: 1.90 (1.7-2.1) g/kg/d | Total protein:  1.19 (0.96-1.26) g/kg/d  Protein D3: 0.82 (0.66-1.19) g/kg/d  Protein D7: 1.34 (1.10-1.45) g/kg/d |
| Carteron 2021 (France) | **Goal Volume:** 42 ml/h for males and females with an estimated ideal body weight of ≤60kg; 63 ml/h for others.  **E**: EN formula with caloric density 1.5 kcal/ml  **P**: EN formula with 9.4 g of hydrolyzed protein per 100ml | **Goal Volume:** 42 ml/h for males and females with an estimated ideal body weight of ≤60kg; 63 ml/h for others.  **E**: EN formula with caloric density 1.5 kcal/ml  **P**: EN formula with 7.5 g of protein per 100ml | IBW by using the Lorentz’s Formula | | 20.2±6.3 kcal/kg/d | 21.0±6.5 kcal/kg/d | 1.3±0.4 g/kg/d | 1.1±0.3 g/kg/d |
| Dresen 2021 (Germany) | **E**: Repeated IC or Harris-Benedict. Multiply with an illness-specific coefficient  **P**: 1.8 g/kg/d | **E**: Repeated IC or Harris-Benedict. Multiply with an illness-specific coefficient  **P**: 1.2 g/kg/d | IBW at BMI 25 | | 1989.3±655.2 kcal/d  27±8.9 kcal/kg/d  97±34% | 1951±828 kcal/d  24.6±9.8 kcal/kg/d  87±30% | 112.4±35 g/d  1.5±0.5 g/kg/d  86±26% | 81.8±31.9 g/d  1.0±0.4 g/kg/d  86±32% |
| Kagan 2022*  (Israel) | **E:** Provision of at least 80% of energy requirements determined via indirect calorimetry  **P:** Protein-enriched EN formula with 25% of calories form protein | **E:** Provision of at least 80% of energy requirements determined via indirect calorimetry  P: EN formula providing 16.7% of calories from protein | - | | 1372.7±530.8 kcal/d | 1648.2±375.8 kcal/d | 83.7±31.9 g/d | 67.2±20.2 g/d |
| Heyland 2023  (International) | **E:** no control for total energy dose; clinicians were encouraged to avoid overfeeding energy and  use published guidelines  **P:** 2.2 g/kg/d or more | **E:** no control for total energy dose; clinicians were encouraged to avoid overfeeding energy and  use published guidelines  **P:** 1.2 g/kg/d or less | Pre-ICU actual dry weight, patients with a BMI above 30 kg/m², IBW based on a BMI of 25 kg/m² was used | | 14.7±6.9 kcal/kg/d | 13.2±6.4 kcal/kg/d | 1.6±0.5 g/kg/d | 0.9±0.3 g/kg/d |

Data are presented as Mean±SD or Median (Q1-Q3).

^£^Information from the author

# Saffle 1990 randomized patients to indirect calorimetry vs predictive equation, then further randomized them to high vs low protein formula. The nutritional intake is reported in 4 groups. They are combined by higher versus lower protein group using an online calculator: <https://home.ubalt.edu/ntsbarsh/business-stat/otherapplets/Pooled.htm>.

^Bukhari 2020 has 3 groups: control (n=22), high-protein polymeric (n=19) and oligomeric group (n=14), the control group was excluded from the analysis.

*Kagan 2022 has 3 groups: Group 1 (control, n=22), Group 2 (low protein + cycle, n=21), and Gorup 3 (high protein + cycle, n=19). Group 2 was excluded from the analysis.

ABW: actual body weight, BMI: body mass index, CURR: Curreri formula, E: energy, EN: enteral nutrition, IBW: ideal body weight, IC: indirect calorimetry, MEE: measured energy expenditure, N_2_: nitrogen intake, P: protein, TBI: traumatic brain injury

Nitrogen intake (gram) / 0.16 = protein intake (gram)

## Table S7: Details of the Early Physical Rehabilitation Intervention

| **Author, year** | **Intervention** | **Device** | **Start time & duration** | **Frequency** | **Duration of Intervention** | **Control group** |
| --- | --- | --- | --- | --- | --- | --- |
| Badjatia 2020 | Neuromuscular electrical stimulation (NMES) | L300 Plus® system (Bioness,Inc,Valencia, CA) | Unclear | Two 30-minute sessions /day | Up to 14 days | No NMES |
| Azevedo 2021 | Cycle ergometry | Moto Med Letto II (ReckTechnik, Germany). | Immediately after randomization | Two 15-minute sessions /day | Up to 21 days | Usual ICU physiotherapy care |
| Kagan 2022 | Cycle ergometry | MOTOmed viva2, Medimotion, Carmarthenshire, Wales, United Kingdom, SA39 9AZ | First 24h after randomization | **Sedated**: passive cycling for 20 minutes  **If able to cycle actively**: two bouts of 10 minutes of more interval when needed | Up to 28 days | Usual physiotherapy care adjusted to the individual needs and a standardized mobilization session of the upper and lower extremities, 5 days per week. Minimum 20 minutes/ session |

## Table S8: Critical Care Nutrition Methodological Quality Scores of all the Included studies

| **Author, year (country)** | **Concealed**  **Randomization** | **Intention-to-treat**  **Analysis** | **Blinding** | **Patient**  **Selection** | **Comparability**  **of groups at**  **baseline** | **Extent of**  **Follow-up** | **Description**  **of treatment**  **protocol** | **Description**  **of treatment**  **co-interventions** | **Objectivity**  **of the definition**  **of outcomes** | **Total score**  **(max 14)** |
| --- | --- | --- | --- | --- | --- | --- | --- | --- | --- | --- |
| 1. Clifton 1985  (USA) | 1 | 2 | 0 | 0 | 1 | 1 | 1 | 1 | 2 | **9** |
| 2. Saffle 1990  (USA) | 1 | 2 | 0 | 1 | 0 | 1 | 0 | 1 | 1 | **7** |
| 3. Mesejo 2003  (Spain) | 2 | 2 | 0 | 0 | 0 | 1 | 1 | 0 | 1 | **7** |
| 4. Zhou 2006  (China) | 1 | 2 | 0 | 0 | 1 | 1 | 0 | 0 | 2 | **7** |
| 5. Singer 2007  (Israel) | 1 | 2 | 0 | 0 | 0 | 1 | 0 | 0 | 1 | **5** |
| 6. Rugeles 2013  (Columbia) | 2 | 0 | 2 | 0 | 1 | 0 | 0 | 0 | 2 | **7** |
| 7. Doig 2015  (Australia) | 2 | 0 | 0 | 1 | 0 | 0 | 1 | 1 | 2 | **7** |
| 8. Ferrie 2015  (Australia) | 2 | 2 | 2 | 1 | 1 | 1 | 1 | 0 | 2 | **12** |
| 9. Jakob 2017  (Switzerland) | 1 | 2 | 2 | 1 | 1 | 1 | 1 | 0 | 2 | **11** |
| 10. Fetterplace 2018  (Australia) | 2 | 2 | 1 | 1 | 1 | 0 | 1 | 0 | 2 | **10** |
| 11. Van Zanten 2018  (Netherlands) | 2 | 2 | 2 | 1 | 1 | 1 | 1 | 0 | 2 | **12** |
| 12. Vega-Alava 2018  (Philippines) | 2 | 2 | 0 | 1 | 1 | 1 | 1 | 1 | 1 | **10** |
| 13. Azevedo 2019  (Brazil) | 1 | 0 | 0 | 0 | 0 | 1 | 1 | 0 | 2 | **5** |
| 14. Danielis 2019  (Italy) | 2 | 2 | 0 | 0 | 0 | 1 | 1 | 0 | 1 | **7** |
| 15. Bukhari 2020  (Indonesia) | 1 | 0 | 0 | 1 | 0 | 0 | 1 | 0 | 2 | **5** |
| 16. Chapple 2020  (Australia) | 2 | 0 | 2 | 1 | 1 | 1 | 1 | 0 | 2 | **10** |
| 17. Nakamura 2020  (Japan) | 2 | 0 | 1 | 1 | 0 | 1 | 1 | 0 | 1 | **7** |
| 18. Carteron 2021  (France) | 2 | 0 | 0 | 1 | 1 | 1 | 1 | 1 | 1 | **8** |
| 19. Dresen 2021  (Germany) | 2 | 0 | 1 | 1 | 0 | 0 | 1 | 1 | 2 | **8** |
| 20. Heyland 2023 (International) | 2 | 0 | 1 | 1 | 1 | 1 | 1 | 2 | 2 | **11** |
| **Studies with combined high protein and early physical rehabilitation** | | | | | | | | | | |
| 21. Badjatia 2020  (USA) | 1 | 2 | 1 | 1 | 1 | 1 | 1 | 0 | 1 | **9** |
| 22. Azevedo 2021  (Brazil) | 1 | 0 | 0 | 0 | 1 | 1 | 1 | 0 | 2 | **6** |
| 23. Kagan 2022 (Israel) | 2 | 2 | 0 | 0 | 1 | 1 | 1 | 2 | 2 | **11** |

## Table S9: Outcomes summary

| **Study** | **Mortality n(%)** | | **Infections n(%)** | | **Duration of Ventilation and Length of Stays (n)** | | **Muscle Mass**  **and Strength (n) or n (%)** | | **Functional, Quality of Life outcomes and Discharge location (n)** | |
| --- | --- | --- | --- | --- | --- | --- | --- | --- | --- | --- |
|  | **Higher Protein** | **Lower Protein** | **Higher Protein** | **Lower Protein** | **Higher Protein** | **Lower Protein** | **Higher Protein** | **Lower Protein** | **Higher Protein** | **Lower Protein** |
| **1) Clifton 1985** | **3-mo**  1/10 (10) | **3-mo**  1/10 (10) | **Not specified**  3/10 (30) | **Not specified**  2/10 (20) | **NR** | **NR** | **NR** | **NR** | **NR** | **NR** |
| **2) Saffle 1990^1^** | **Not specified**  CURR: 1/11 (9.1)  IC: 2/14 (16.6) | **Not specified**  CURR: 1/12 (8.3)  IC: 1/12 (7.1) | **NR** | **NR** | **Hospital**  CURR: 57.1±8.2 (11)  IC: 46.6±5.7 (14)  Pooled: 51.22±6.9 (25) | **Hospital**  CURR: 40.8±4.8 (12)  IC: 51.6±7.3 (12)  Pooled: 46.2±6.2 (24) | **NR** | **NR** | **NR** | **NR** |
| **3) Mesejo 2003** | **ICU**  7/24 (29.2) | **ICU**  8/26 (30.8) | **Hospital-acquired infection**  8/24 (33.3) | **Hospital-acquired infection**  10/26 (38.5) | **MV**  9.4±5.96 (24)  **ICU**  14.8±8.76 (24) | **MV**  8.7±6.18 (26)  **ICU**  14.8±9.39 (26) | **NR** | **NR** | **NR** | **NR** |
| **4) Zhou 2006** | **28-d**  7/25 (28)  **90-d**  11/25 (44) | **28-d**  10/26 (38.5)  **90-d**  11/26 (42.3) | **NR** | **NR** | **NR** | **NR** | **NR** | **NR** | **NR** | **NR** |
| **5) Singer 2007^$^** | **ICU**  3/8 (37.5) | **ICU**  2/6 (33.3) | **NR** | **NR** | **NR** | **NR** | **NR** | **NR** | **NR** | **NR** |
| **6) Rugeles 2013** | **NR** | **NR** | **NR** | **NR** | **MV**  8.5±4.6 (n=40)  **ICU**  9.5±5.5 (n=40) | **MV**  9.7±4.9 (n=40)  **ICU**  10.4±5.0 (n=40) | **NR** | **NR** | **NR** | **NR** |
| **7) Doig 2015** | **ICU**  28/239 (11.7)  **Hospital**  37/239 (15.5)  **90-d**  42/236 (17.8)  ^$^Subgroup of patients with baseline kidney dysfunction or risk of progression of AKI: 17/60 (28.3) | **ICU**  30/235 (12.8)  **Hospital**  43/235 (18.3)  **90-d**  47/235 (20.0)  ^$^Subgroup of patients with baseline kidney dysfunction or risk of progression of AKI: 7/46 (15.2) | **NR** | **NR** | **MV**  7.33 (7.00-7.68) (239)  **ICU**  11.6 (10.8 to 12.5) (239)  **Hospital**  26.0 (24.2 to 28.0) (239) | **MV**  7.26 (6.94-7.61) (235)  **ICU**  10.7 (10.0 to 11.5) (235)  **Hospital**  24.8 (23.0 to 26.6) (235) | **NR** | **NR** | **D90 RAND-36 General Health**  50.5±27.2 (192)  **D90 ECOG Performance Status**  1.31±1.0 (192)  **D90 RAND-36 Physical Function**  47.7±33.7 (192) | **D90 RAND-36 General Health**  52.8±25.9 (180)  **D90 ECOG Performance Status**  1.18±1.0 (180)  **D90 RAND-36 Physical Function**  53.2±33.0 (180) |
| **8) Ferrie 2015** | **ICU**  8/59 (14)  **Hospital**  12/59 (20)  **6-mo**  15/59 (25) | **ICU**  6/60 (10)  **Hospital**  9/60 (15)  **6-mo**  9/60 (15**)** | **NR** | **NR** | **MV**  2 (1-3)  4.87±14.37^£^(59)  **ICU**  5 (3-8)  9.85±14.83^£^(59)  **Hospital**  25.0 (16.8-41.3)  41.75±37.36^£^(59) | **MV**  2 (1-5)  2.67±6.16^£^(59)  **ICU**  6 (3.8-10.0)  9.85±14.83^£^(60)  **Hospital**  27.5 (18.8-55.8)  37.70±35.88^£^(60) | **D7 forearm thickness, cm**  3.2±0.4 (35)^£^  **D7 biceps thickness, cm**  2.5±0.6 (35) ^£^  **D7 thigh area, cm^2^**  6.8±2.1(n=29) ^£^  **D7 ∑3 muscle, cm**  8.4±1.0 (39) ^£^  **D7 change in thigh area, %**  -8.4±31.1 (28)^£^  **D7 HG Str** 22.1±10.1 (52) | **D7 forearm thickness, cm**  2.8±0.4 (35) ^£^  **D7 biceps thickness, cm**  2.4±0.4 (35) ^£^  **D7 thigh area, cm^2^**  5.8±1.9 (28) ^£^  **D7 ∑3 muscle, cm**  7.9±1.1 (33) ^£^  **D7 change in thigh area, %**  30±139.2 (29)^£^  **D7 HG Str**  18.5±11.8 (56) | **NR** | **NR** |
| **9) Jakob 2017** | **NR** | **NR** | **Secondary infection**  19/46 (41.3) | **Secondary infection**  19/44 (43.2) | **MV**  6.2 (4.8-7.7)  2.8±2.6^£(^46)  **ICU**  7.0 (5.3-8.7)  9.0±7.7^£^(46)  **Hospital**  31.0 (27.0-35.0)  21.7±12.4^£^(46) | **MV**  7.0 (4.7-9.3)  4.1±3.0^£^(44)  **ICU**  10.0 (6.6-13.4)  11.4±10.4^£^(44)  **Hospital**  36.0 (29.9-42.1)  21.7±11.0^£^(44) | **NR** | **NR** | **NR** | **NR** |
| **10) Fetterplace 2018** | **28-day**  4/30 (13.3)  **60-day**  4/30 (13.3) | **28-day**  5/30 (16.7)  **60-day**  5/30 (16.7) | **NR** | **NR** | **MV**  8.7±7.5 (30)  **ICU**  10.6±8.3 (30)  **Hospital**  27.4±19.0 (30) | **MV**  7.0±5.0 (30)  **ICU**  9.1±5.5 (30)  **Hospital**  18.8±10 9 (30) | **QMLT loss at D15/ICUDC**  12.73±18.05 (24)^£^  **Best HGS kg at awakening or ICU DC or D15**  20±6.1 (6)  **MRC score** **at awakening or ICU DC or D15**  55±5.9 (7) | **QMLT loss at D15/ICUDC**  21.25±17.67 (23)^£^  **Best HGS kg at awakening or ICU DC or D15**  21±9.3 (16)  **MRC score** **at awakening or ICU DC or D15**  52±9.6 (14) | **Scored Physical Function in ICU Test**  6.8±3.8 (8)  **Discharge to rehab facility**  12/30 (40%) | **Scored Physical Function in ICU Test**  7.9±3.4 (14)  **Discharge to rehab facility**  13/30 (43%) |
| **11) van Zanten 2018** | **28-day**  2/22 (9.1)  **42-day**  3/22 (13.6)  **ICU**  1/22 (4.5)  **Hospital**  2/22 (9.1) | **28-day**  3/22 (13.6)  **42-day**  3/22 (13.6)  **ICU**  2/22 (9.1)  **Hospital**  3/22 (13.6) | **NR** | **NR** | **MV**  10.0±8.7 (22)  **ICU**  18.4±13.4 (22)  **Hospital**  28.5±13.3 (22) | **MV**  7.4±5.4 (22)  **ICU**  18.3±12.7 (22)  **Hospital**  28.2±13.2 (22) | **NR** | **NR** | **NR** | **NR** |
| **12) Vega-Alava 2018** | **Not specified**  0/20 | **Not specified**  0/20 | **VAP**  0/20 (0) | **VAP**  5/20 (25) | **MV**  5.4 (no SD) (20) | **MV**  7.45 (no SD) (20) | **NR** | **NR** | **NR** | **NR** |
| **13) Azevedo 2019** | **ICU**  22/57 (38.5)  **Hospital**  26/57 (45.6) | **ICU**  28/63 (44.4)  **Hosp**  29/63 (46.0) | **NR** | **NR** | **MV**  9 (5-14) (57)  **ICU**  21 (13-33) (57) | **MV**  9 (5-14) (63)  **ICU**  18 (10-35) (63) | **HGS at ICU discharge, kg**  Male: 18 (15-25)  (15)  Female: 8 (2-17) (9) | **HGS at ICU discharge, kg**  Male: 23.5 (13.7-32.0) (14)  Female: 14 (7-22.5) (13) | **SF-36 PCS 3-mo**  93.6±126.1 (55)  **SF-36 PCS 6-mo**  92.0±133.4 (52) | **SF-36 PCS 3-mo**  85.2±110.6 (59)  **SF-36 PCS 6-mo**  90.0±120.6 (58) |
| **14) Danielis 2019** | **ICU**  2/19 (11) | **ICU**  7/21 (33) | **NR** | **NR** | **MV**  9.1±7.5^£^(19)  **ICU**  14.5±7.2^£^(19) | **MV**  9.3±4.6^£^(21)  **ICU**  16.0±6.5^£^(21) | **NR** | **NR** | **NR** | **NR** |
| **15) Badjatia 2020** | **PBD 90**  0/12 | **PBD 90**  0/13 | **Hosp-acquired infection**  3/12 (25) | **Hosp-acquired infection**  6/13 (46) | **ICU**  18±7 (12) | **ICU**  20±8 (13) | **CT mid-thigh cross-sectional area atrophy, %**  6.5±4.1 (12) | **CT mid-thigh cross-sectional area atrophy, %**  12.5±6.4 (13) | **Modified Rankin Scale**  D14: 4 (2-4)  D90: 1 (0-2)  **SPPB**  D14: 2 (0-7.8)  D90: 12 (10-12)  **D90 Short-form NeuroQOL**  i) Fatigue: 29±15  ii) Lower extremity mobility: 90±8 (12)  iii) Cognition: 35±5 | **Modified Rankin Scale**  D14: 4 (3-5)  D90: 2 (1-3)  **SPPB**  D14: 1 (0-5)  D90: 9 (4-12)  **D90 Short-form NeuroQOL**  i) Fatigue: 41±28  ii) Lower extremity mobility:  73±27 (13)  iii) Cognition: 31±12 |
| **16) Bukhari 2020^2^** | **Hospital**  7/19 (36.8) | **Hospital**  3/14 (21.4) | **NR** | **NR** | **ICU**  9.38±6.80 (13)  **Hospital**  18.38±9.51 (13) | **ICU**  9.09±5.53 (11)  **Hospital**  24.73±14.29 (11) | **NR** | **NR** | **NR** | **NR** |
| **17) Chapple 2020** | **ICU**  12/58 (21)  **28-d**  12/56 (21)  **90-d**  14/55 (26) | **ICU**  10/58 (17)  **28-d**  14/57 (25)  **90-d**  15/56 (27) | **NR** | **NR** | **28-d MV free day**  18±9 (58)  **ICU**  13±13 (58)  **Hospital**  24±21 (58) | **28-d MV free day**  18±9 (58)  **ICU**  14±18 (58)  **Hospital**  26±32 (58) | **NR** | **NR** | **D90 EQ-5D-5L** (41)  i) Mobility: 2.2±1.3  ii) Self-care: 2±1.2  iii) Usual activities: 2.5±1.3  iv) Pain or discomfort: 1.8±0.9  v) Anxiety/ Depression: 1.8±0.9  vi) VAS: 60±32  **D90 Discharge to Rehab Facility**  5/41 (12.2%) | **D90 EQ-5D-5L** (41)  i) Mobility: 2±1.3  ii) Self-care: 1.7±1.3  iii) Usual activities: 2.1±1.3  iv) Pain or discomfort: 1.9±0.9  v) Anxiety/ Depression: 1.8±0.9  vi) VAS: 53±32  **D90 Discharge to Rehab Facility**  6/41 (14.6%) |
| **18) Nakamura 2020** | **28-d Survival**  90.0% (n=60) | **28-d Survival**  89.5% (n=57) | **NR** | **NR** | **MV**  5.0±3.0^£^(60)  5 (2-6.5)  **ICU**  8.5±4.8^£^(60)  7 (5-12)  **Hospital**  43.5±39.3^£^(60)  26.5 (18-58) | **MV**  5.9±3.1^£^(57)  5.5 (3-9)  **ICU**  9.6±5.1^£^(57)  9 (6-13)  **Hospital**  50.4±35.6^£^(57)  45.5 (18.25-75.75) | **Femoral muscle volume loss, %**  12.9±8.5 (60) | **Femoral muscle volume loss, %**  16.9±7.0 (57) | **FSS-ICU ICU discharge**  15.5 (3-30.75)  **Barthel Index Hospital discharge**  62.5 (0-91.25)  **EQ-5D Hospital discharge**  8 (5-14.5) | **FSS-ICU ICU discharge**  18 (2.5-35)  **Barthel Index Hospital discharge**  12.5 (1-93.75)  **EQ-5D Hospital discharge**  8 (5-14) |
| **19) Azevedo 2021** | **ICU**  23/87 (26.4)  **Hospital**  25/87 (31.2  **6-months**  29/7 (33.3) | **ICU**  41/94 (43.6)  **Hospital**  47/94 (53.4)  **6-months**  51/94 (54.2) | **NR** | **NR** | **MV**  10 (5-19) (87)  **ICU**  18 (12-36) (87)  **Hospital**  38 (18-70) (87) | **MV**  12 (7-21) (94)  **ICU**  23 (16-36**)**  **Hospital**  40 (21-60) (94) | **ICU acquired weakness** 29.1% (16/87)  **Handgrip strength at ICU discharge or day 21**  **Male:** 17.5±11.9 (N-not response)  **Female:**10.8±7.0 (N-not response) | **ICU acquired weakness** 46.4% (26/94)  **Handgrip strength at ICU discharge or day 21**  **Male:** 14.5±12.2 (N-not response)  **Female:**8.1±7.6 (N-not response) | **PCS score  3-months**  24.4 (0.00-49.12)  33.5±31.8^£^  (87)  **PCS score  6-months**  33.63 (0.00-71.61)  40.0±36.4^£^  (87) | **PCS score  3-months**  0.00 (0.00-37.00)  24.5±32.5^£^  (94)  **PCS score  6-months** 0.00 (0.00-55.1)  27.0±35.9^£^  (94) |
| **20) Carteron 2021** | **28-d**  20/100 (20)  **60-d**  23/100 (23) | **28-d**  21/95 (22)  **60-d**  23/95 (24) | **Pneumonia**  47/100 (47) | **Pneumonia**  41/95 (43) | **MV**  10 (6-16)  12±9^£^(100)  **ICU**  14 (8-21)  16±11^£^(100) | **MV**  11 (6-17)  13±9^£^(95)  **ICU**  15 (10-23)  18±11^£^(95) | **NR** | **NR** | **NR** | **NR** |
| **21) Dresen 2021** | **ICU**  8/21 (38)  **28-d**  2/21 (9.5) | **ICU**  7/21 (33)  **28-d**  4/21 (19.0) | **Pneumonia in ICU**  19/21 (90)  **Wound infection in ICU**  11/21 (52) | **Pneumonia in ICU**  17/21 (81)  **Wound infection in ICU**  11/21 (52) | **MV in ICU**  1372±642h  ~57.2±26.8d (21)  **MV in the study period**  797±133h  ~33.2±5.5d (21)  **ICU**  68±34d (21) | **MV in ICU**  1350±1170h  ~56.3±48.8d (21)  **MV in the study period**  758±191h  ~31.6±8.0d (21)  **ICU**  62±48d (21) | **QMLT mid right, mm**  -0.19±0.13  **QMLT, mid left, mm**  -0.18±0.10  **QMLT 2/3 right, mm**  -0.13±0.08  **QMLT, 2/3 left, mm**  -0.08±0.06  **Mean of all 4 measurements, mm (daily changes)**  -0.15±0.08  **Mean decrease of all 4 measurements in 28 days**  -3.4±1.8 mm  (30.4±11.7%) (15) | **QMLT mid right, mm**  -0.36±0.12  **QMLT, mid left, mm**  -0.24±0.10  **QMLT 2/3 right, mm**  -0.28±0.08  **QMLT, 2/3 left, mm**  -0.21±0.06  **Mean of all 4 measurements, mm (daily changes)**  -0.28±0.08  **Mean decrease of all 4 measurements in 28 days**  -5.7±2.5 mm  (51.8±21.1%) (12) | **NR** | **NR** |
| **22) Kagan 2022^3^** | **ITT:**  **ICU**  3/19 (14.3)  **Hospital**  5/19 (23.8)  **PP:**  **ICU**  3/14 (21.4)  **Hospital**  5/14 (35.7) | **1) ITT:**  **ICU**  1/22 (4.5)  **Hospital**  4/22 (18.2)  **PP:**  **ICU**  1/13 (7.7)  **Hospital**  4/13 (30.8)  **2) ITT:**  **ICU**  3/21 (14.3)  **Hospital**  5/21 (23.8)  **PP:**  **ICU**  3/14 (21.4)  **Hospital**  4/14 (28.6) | **NR** | **NR** | **ITT:**  **MV**  11.7±9.7 (19)  **ICU**  18.8±10.5 (19)  **Hospital**  35.2±25.7 (19)  **PP:**  **MV**  14.9±9.4 (14)  **ICU**  20.8±10.1 (14)  **Hospital**  36.2±27.4 (14) | **1) ITT**  **MV**  10.2 9.5 (22)  **ICU**  17.2±9.6 (22)  **Hospital**  33.1±22.6 (22)  **PP**  **MV**  14.2±9.6 (13)  **ICU**  19.9±10.1 (13)  **Hospital**  40.8±23.4 (13)  **2) ITT**  **MV**  12.0±7.8 (19)  **ICU**  16.3±7.7 (19)  **Hospital**  26.5±17,0 (19)  **PP**  **MV**  15.8±7.1 (14)  **ICU**  19.3±7.2 (14)  **Hospital**  30.7±17.9 (14) | **NR** | **NR** | **NR** | **NR** |
| **23) Heyland 2023** | **60-day**  222/642 (34.6)  ^$^Subgroup of AKI patients:82/162 (50.6) | **60 day**  208/648 (32.1)  ^$^Subgroup of AKI patients:53/146 (36.3) | **NR** | **NR** | **All patients:**  **MV**  6.1 (3.0-13.8 (645)  **ICU**  10.0 (5.6-18.2) (642)  **Hospital**  19.3 (9.7-39.0) (642)  **60-day survivors:**  **MV**  5.7 (2.8-12.6) (420)  **ICU**  10.2 (5.8-20.3) (420)  **Hospital**  25.2 (13.8-50.7) (420) | **All patients:**  **MV**  6.1 (2.8-12.8) (656)  **ICU**  9.4 (5.1-19.2) (650)  **Hospital**  18.9 (9.4-37.9) (647)  **60-day survivors:**  **MV**  5.8 (2.2-11.8) (440)  **ICU**  9.9 (5.0-20.6) (440)  **Hospital**  23 (12.9-47.1) (439) | **NR** | **NR** | **NR** | **NR** |

CT: computed tomography, ECOG: Eastern Collaborative Oncology Group, FSS-ICU: functional status score in the ICU, HGS: handgrip strength, ICU: intensive care unit, MRC: medical research council, NR: not reported, PCS: physical component score, QMLT: quadriceps muscle layer thickness, RAND: public domain version of the Short Form 36, SF-36: short-form 36 quality of life questionnaire, SPPB: short physical performance battery, VAP: ventilator-associated pneumonia, VAS: Visual Analogue Scale

£: Information obtained from the author

^1^Saffle 1990 randomized patients to indirect calorimetry vs predictive equation, then further randomized them to high vs low protein formula. The outcomes are reported in 4 groups. They are combined by higher versus lower protein group using an online calculator: <https://home.ubalt.edu/ntsbarsh/business-stat/otherapplets/Pooled.htm>.

^2^Bukhari 2020 has 3 groups: control (n=22), high-protein polymeric (n=19) and oligomeric group (n=14), the control group was excluded from the analysis. The results of TBI and Non-TBI group were combined.

^3^Kagan 2022 has 3 groups: Group 1 (control, n=22), Group 2 (low protein + cycle, n=21), and Gorup 3 (high protein + cycle, n=19). Group 2 was excluded from the analysis.

^$^Definition of AKI, baseline kidney dysfunction or risk of progression of AKI:

- Singer 2007: Acute renal failure was defined as a 50% decrease in GFR, a doubling of serum creatinine or an increase of creatinine to 3.5 mg/dL (309.4 umol/L).
- Doig 2015 (mortality of paitents with kidney dysfunction or risk of progression of AKI from Doig 2015 is 90-d mortality from their secondary publication ^29^)
  - Baseline kidney dysfunction: Creatinine at time of enrolment > 168μmol/L (by Gordon Bernard’s *“*Brussels Table”)
  - Risk of progression of AKI at enrollment: was defined as a rise in creatinine over the previous 24 hours by at least 20% to over 120 μmol/L
- Heyland 2023: Acute kidney injury refers to patients who met the criteria of KDIGO:
  - stage 1 is at least 26·52 μmol/L increase in serum creatinine from baseline within 48 h or 1·5–1·9 times baseline within 7 days
  - stage 2 is 2·0–2·9 times baseline within 7 days
  - stage 3 is three times or more baseline within 7 days or increase to at least 353·6 μmol/L with an acute increase of more than 44·2 μmol/L.

Note: continuous data are presented as mean±SD (n) or median (quartile 1-qurtile 3) (n), unless otherwise stated.

## Table S10: Biochemical Outcomes Summary

| **Study** | **Higher protein** | **Lower protein** |
| --- | --- | --- |
| Clifton 1985 | Nitrogen balance, g/d  -5.3±5.0(10)  -0.08±0.07 g/kg/d (10)  Urinary urea nitrogen, g/d  26.7±4.9 g/d (10)  Serum albumin, g/dL  3.1±0.5 (10)  31±5 g/L  Total lymphocyte count, mm^3^  1509±498 (10) | Nitrogen balance, g/d  -9.2±6.7 (10)  -0.13±0.09 g/kg/d (10)  Urinary urea nitrogen, g/d  18.9±5.2 g/d (10)  Serum albumin g/dL  3.1±0.5 (10)  31±5 g/L  Total lymphocyte count, mm^3^  1608±783 (10) |
| Saffle 1990 | Nitrogen balance, g/d  5.27±3.94 (25)  Cumulative Nitrogen balance, g  42.3±21.46 (25)  Urinary nitrogen, g/d  18.30±1.75 (25)  Albumin, g/dL  2.03±0.13 (25)  20.3±1.3 g/L  Total lymphocyte count, mm^3^  1809.7±268.2 (25) | Nitrogen balance, g/d  0.25±2.55 (24)  Cumulative Nitrogen balance, g  0.75±10.42 (24)  Urinary nitrogen, g/d  16.15±1.54 (24)  Albumin, g/dL  1.85±0.12 (24)  18.5±1.2 g/L  Total lymphocyte count, mm^3^  1540±228.6 (24) |
| Mesejo 2003 | Plasma glucose level, mg/dL  222.8±47.12 (24)  12.4±2.6 mmol/L  Capillary glucose level, mg/dL  216.4±56.75 (24)  12.0±3.2 mmol/L  Insulin/d, IU  30.2 (21.4-57.1) (24) | Plasma glucose level, mg/dL  176.8±44.01 (26)  9.8±2.4 mmol/L  Capillary glucose level, mg/dL  163.1±45.62 (26)  9.1±2.5 mmol/L  Insulin/d, IU  8.73 (2.3-27.5) (26) |
| Zhou 2006 | D7 Nitrogen balance, g  -2.89±6.15 (25)  D7 Blood glucose level, mmol/L  8.89±3.67 (25)  D7 Albumin g/L  32.4±4.8 (25)  D7 Prealbumin, mg/L  185.76±62.16 (25)  18.58±6.22 mg/dL  D7 Triglycerides, mmol/L  3.07±2.5 (25)  D7 Hemoglobin, g/L  119.8 ±35.82 (25) | D7 Nitrogen balance, g  -3.57±4.62 (26)  D7 Blood glucose level, mmol/L  7.79±2.39 (26)  D7 Albumin, g/L  31.7±4.7 (26)  D7 Prealbumin, mg/L  160.15±58.85 (26)  16.02±5.89 mg/dL  D7 Triglycerides, mmol/L  2.89±0.79 (26)  D7 Hemoglobin, g/L  131.15±21.33 (26) |
| Singer 2007 | D4 Blood urea nitrogen, mg/dL  64.3±6.4 (8)  22.96±2.28 mmol/L  D4 Creatinine, mg/dL  1.9±0.6 (8)  167.96±53.04 umol/L  D4 Nitrogen balance, g  8.0±3.8 (8) | D4 Blood urea nitrogen, mg/dL  59.0±5.1 (6)  21.06±1.82 mmol/L  D4 Creatinine, mg/dL  2.5±0.7 (6)  221.0±61.88 umol/L  D4 Nitrogen balance, g  -8.1±19 (6) |
| Rugeles 2013 | Insulin requirement, IU  10.4±17.8 (40) | Insulin requirement, IU  14.0±22.1 (40) |
| Doig 2015 | D7 Urea, mmol/L  16.2 (estimated from eFigure2) | D7 Urea, mmol/L  10.8 (estimated from eFigure2) |
| Ferrie 2015 | D3 Nitrogen balance, g/d  -0.5±3.1 (60)  D7 Nitrogen balance, g/d  -4.9±7.9 (60) | D3 Nitrogen balance, g/d  -5.6±1.8 (59) (p<0.0001)  D7 Nitrogen balance, g/d  -4.7±2.4 (59) (p=0.92) |
| Jakob 2017 | Creatinine at the end of EN  87.0 (56.0-113.0) (39)  Serum albumin at treatment end or at ICU discharge, g/L  21.0 (19.0-26.0) (17)  Hemoglobin at the end of EN, g/L  97.0 (88.8-110.8) (29)  **All data below are provided by the Author**  Urea per patient per day after day 0, mmol/L  16.08±8.57 (45)  Creatinine per patient per day after day 0, umol/L  115.3±80.7 (45)  Hemoglobin per patient per day after day 0, g/L  97.29±17.29 (45)  Albumin per patient per day after day 0, g/L  20.31±5.32 (36)  Glucose per patient per day after day 0, mmol/L  8.65±1.76 (45)  Insulin per patient per day after day 0, unit  39.26±55.99 (45) | Creatinine at the end of EN  100.0 (50.8-203.1) (38)  Serum albumin at treatment end or at ICU discharge, g/L  23.0 (18.0-26.0) (19)  Hemoglobin at the end of EN, g/L  99.0 (89.0-105.0) (31)  **All data below are provided by the Author**  Urea per patient per day after day 0, mmol/L  14.60±10.31 (44)  Creatinine per patient per day after day 0. umol/L  144.2±139.8 (44)  Hemoglobin per patient per day after day 0, g/L  96.06±13.93 (44)  Albumin per patient per day after day 0, g/L  20.45±5.06 (39)  Glucose per patient per day after day 0, mmol/L  8.67±2.31 (44)  Insulin per patient per day after day 0, unit  44.80±54.20 (44) |
| Fetterplace 2018 | - | - |
| Van Zanten 2018 | D5 Blood urea nitrogen, mmol/L  15.7 (7.6-22.6) (22)  15.15±8.02 (22)*  Total urinary nitrogen, g/day  24 (18-27) (22)  25.9±10.3 (22)* | D5 Blood urea nitrogen, mmol/L  11.5 (6.8-14.4) (22)  11.39±5.75 (22)*  Total urinary nitrogen, g/day  18 (14-21) (22)  17.2±7.4 (22)* |
| Vega-Alava 2018 | Albumin prior discharge  ~30.4 (estimated from Figure 3) | Albumin prior discharge  ~28.2 (estimated from Figure 3) |
| Azevedo 2019 | - | - |
| Danielis 2019 | Nitrogen balance at study exit, g   - 1. (-3.4 to 0.85) (19)   Creatinine at study exit, mg/dL  0.8 (0.52 to 0.99) (19) | Nitrogen balance at study exit, g  -8.7 (-11.1 to -5.2) (21)  Creatinine at study exit, mg/dL  0.6 (0.52 to 0.77) (21) |
| Bukhari 2020 | D3 Serum albumin, g/dL  2.99±0.62 (19)  29.9±6.2 g/L  D3 Total lymphocyte count, cells/uL  1058.2±611.3 (19)  D3 White blood cell, cells/uL  11434.2±6577.9 (19)  11.43±6.58 10^3^/uL (or 10^9^/L)  D3 IL-6, ng/mL  105.8±145.5 (19)  10.58±14.55 ng/L | D3 Serum albumin, g/dL  3.04±0.51 (14)  30.4±5.1 g/L  D3 Total lymphocyte count, cells/uL  896.3±600.7 (14)  D3 White blood cell, cells/uL  10696.8±3940.8 (14)  10.70±3.94 10^3^/uL (or 10^9^/L)  D3 IL-6, ng/mL  157.4±254.9 (14)  15.74±25.49 ng/L |
| Chapple 2020 | Mean daily serum urea (highest), mmol/L  14±7 (58)  Creatinine (highest), umol/L  188±185 (58)  Daily blood glucose closest to 0800, mmol/L  8.6±2 (58)  Insulin dose, mean IU/participant/day  56±42 (58)  Phosphate (lowest), mmol/L  1±0.4 (58)  Potassium (lowest), mmol/L  3.9±0.4 (58) | Mean daily serum urea (highest), mmol/L  11±5 (58)  Creatinine (highest), umol/L  209±236 (58)  Daily blood glucose closest to 0800, mmol/L  8.8±1.7 (58)  Insulin dose, mean IU/participant/day  61±42 (58)  Phosphate (lowest), mmol/L  1±0.25 (58)  Potassium (lowest), mmol/L  3.8±0.3 (58) |
| Nakamura 2020 | D10 Blood urea nitrogen, mg/dL  28.5 (16.5-44.3) (60)  33.9±22.3 (60)*  12.11±7.96 mmol/L  D10 Creatinine, mg/dL  0.8 (0.6-1.0) (60)  1.03±0.97 (60)*  91.07±85.77 umol/L  Insulin dose, U/10days  0 (0-96) (60)  61.5±97.6 (60)*  Phosphate, mg/dL  3.3±0.6 (60)  1.07±0.19 mmol/L  D10 CRP, mg/dL  5.25±6.56 (60)  D10 Albumin, g/dL  2.7±0.6 (60)  27±6 g/L  Prealbumin, mg/dL  16.5±7.2 (60)  Lymphocyte counts, uL)  1324.6±857.4 (60) | D10 Blood urea nitrogen, mg/dL  24.9 (18.1-36.4) (57)  33.1±25.5 (57)*  11.82±9.11 mmol/L  D10 Creatinine, mg/dL  0.8 (0.6-1.0) (57)  1.14±1.29 (57)*  100.8±114.06 umol/L  Insulin dose, U/10days  0 (0-68.5) (57)  43.6±77.0 (57)*  Phosphate, mg/dL  3.6±1.6 (57)  1.16±0.52 mmol/L  D10 CRP, mg/dL  5.52±6.33 (57)  D10 Albumin, g/dL  2.6±0.6 (57)  26±6 g/L  Prealbumin, mg/dL  15.6±7.2 (57)  Lymphocyte counts, uL  984.9±728.6 (57) |
| Carteron 2021 | Albumin, g/L*  Day 0: 27±5 (98)  Day 5: 22±5 (83)  Day 10: 23±4 (55)  Prealbumin, g/L*  Day 0: 0.15±0.06 (62)  Day 5: 0.15±0.11 (53)  Day 10: 0.22±0.08 (41) | Albumin, g/L*  Day 0: 27±4 (90)  Day 5: 24±4 (76)  Day 10: 23±4 (60)  Prealbumin, g/L*  Day 0: 0.18±0.10 (62)  Day 5: 0.14±0.06 (50)  Day 10: 0.18±0.06 (36) |
| Dresen 2021 | Urea, mg/dL*  95±61 (21)  33.92±21.78 mmol/L  Creatinine, mg/dL*  1.0±0.9 (21)  88.42±79.58 (21) umol/L  Highest blood glucose, mg/dL*  163±42 (19)  9.1±2.3 mmol/L  Lowest blood glucose, mg/dL*  121±30 (19)  6.7±1.7 mmol/L  CRP, mg/dL*  84±77 (13)  IL-6, ng/L*  51±23 (13)  Albumin, g/L*  26±4 (16)  Leukocytes, 10^9^/l*  14.0±6.1 (21)  Hemoglobin, g/dL*  8.1±1.1 (21)  81±11 g/L | Urea, mg/dL*  71±41 (20)  25.35±14.64 mmol/L  Creatinine, mg/dL*  1.3±1.2 (20)  114.95±106.1 (20) umol/L  Highest blood glucose, mg/dL*  158±49 (19)  8.8±2.7 mmol/L  Lowest blood glucose, mg/dL*  124±35 (19)  6.9±1.9 mmol/L  CRP, mg/dL*  104±110 (7)  IL-6, ng/L*  294±651 (8)  Albumin, g/L*  29±5 (12)  Leukocytes, 10^9^/l*  14.3±10.1 (20)  Hemoglobin, g/dL*  8.7±0.9 (20)  87±9 g/L |
| Heyland 2023 | Highest urea, mmol/L  14.0±8.5 (634)  Creatinine, umol/L  125.8±121.2 (641)  Highest glucose, mmol/L  10.3±3.0 (598)  Lowest glucose, mmol/L  7.2±1.5 (598)  Insulin, total units/d  44.9±48.2 (364)  Highest triglycerides, mmol/L  2.2±1.4 (160)  Lowest phosphate, mmol/L*  2.16±1.35 (613) | Highest urea, mmol/L  11.9±7.2 (647)  Creatinine, umol/L  117.5±115.5 (654)  Highest glucose, mmol/L  10.4±2.9 (597)  Lowest glucose, mmol/L  7.1±1.3 (597)  Insulin, total units/d  40.4±41.1 (365)  Highest triglycerides, mmol/L  2.5±2.0 (175)  Lowest phosphate, mmol/L*  2.10±1.23 (626) |
| Badjatia 2020 | Blood urea nitrogen at post bleeding day 14, mg/dL  23±7 (12)  8.2±2.5 mmol/L  Creatinine at post bleeding day 14, mg/dL  0.6±0.2 (12)  53.04±17.68 umol/L  Glucose at post bleeding day 14, mg/dL  128±31 (12)  7.1±1.7 mmol/L  CRP at post bleeding day 14, mg/dL  4.1±5.7 (12)  D14 Prealbumin, mg/dL  24±7 (12)  D14 White blood cell post bleeding, 10^3^/uL (or 10^9^/L)  12.3±5.0 (12) | Blood urea nitrogen at post bleeding day 14, mg/dL  14±5 (13)  5.0±1.8 mmol/L  Creatinine at post bleeding day 14, mg/dL  0.6±0.2 (13)  53.04±17.68 umol/L  Glucose at post bleeding day 14, mg/dL  125±43 (13)  6.9±2.4 mmol/L  CRP at post bleeding day 14, mg/dL  4.2±6.2 (13)  D14 Prealbumin, mg/dL  26±9 (13)  D14 White blood cell post bleeding, 10^3^/uL (or 10^9^/L)  12.0±3.6 (13) |
| Azevedo 2021 | - | - |
| Kagan 2022 | - | - |

*Data provided by Author

## Figure S1: PRISMA 2020 Flow Diagram


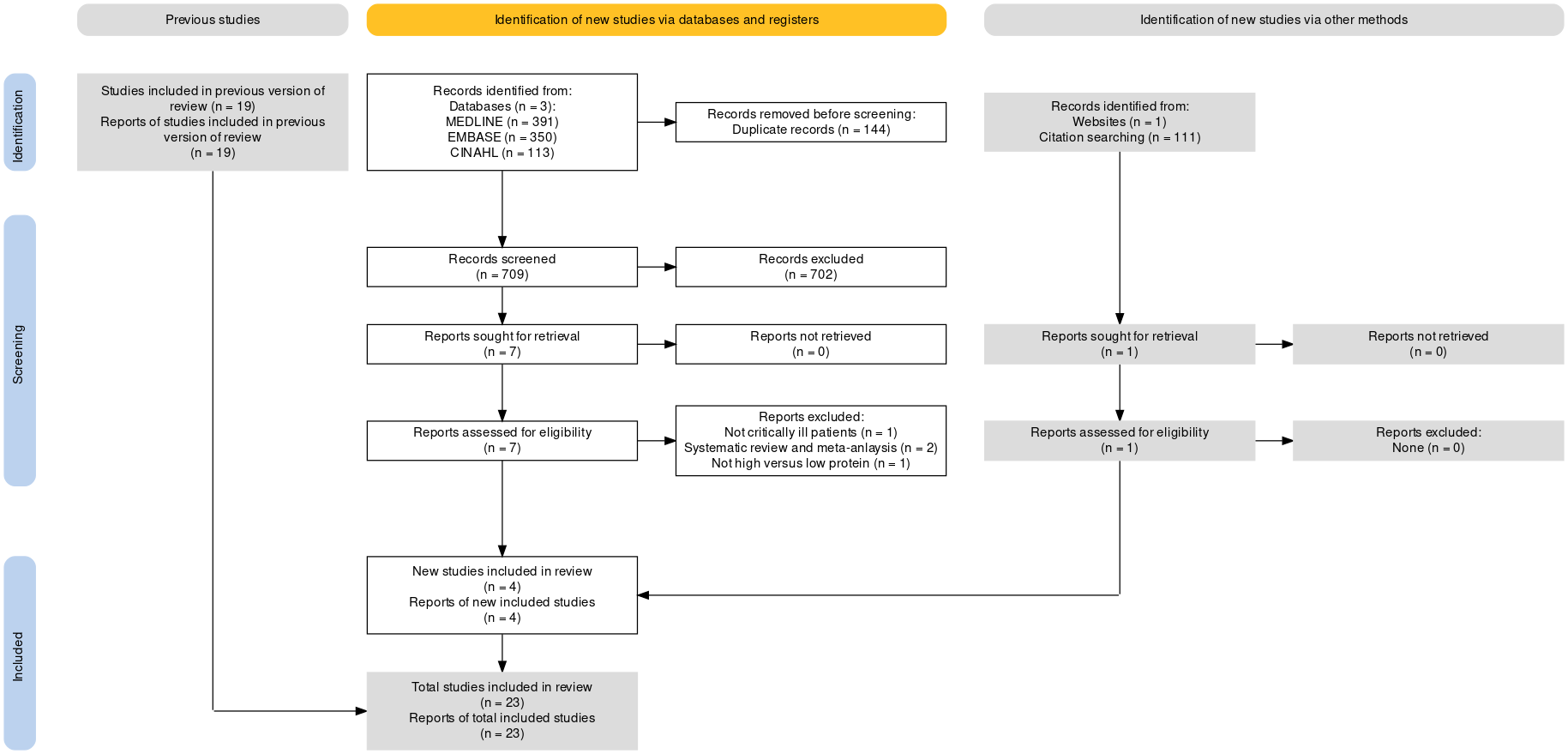


**Note:**

Previous version of the review ^5^

Website is [www.criticalcarenutrition.com](http://www.criticalcarenutrition.com)

Citation screening from Table S4 study 23 and 52

See Table S4 for full list of excluded studies with the reason of exclusion

## Figure S2 Protein (g/kg/day) and Energy (kcal/kg/day) delivered between higher vs lower protein group

| **a) Protein delivered (g/kg/day)**  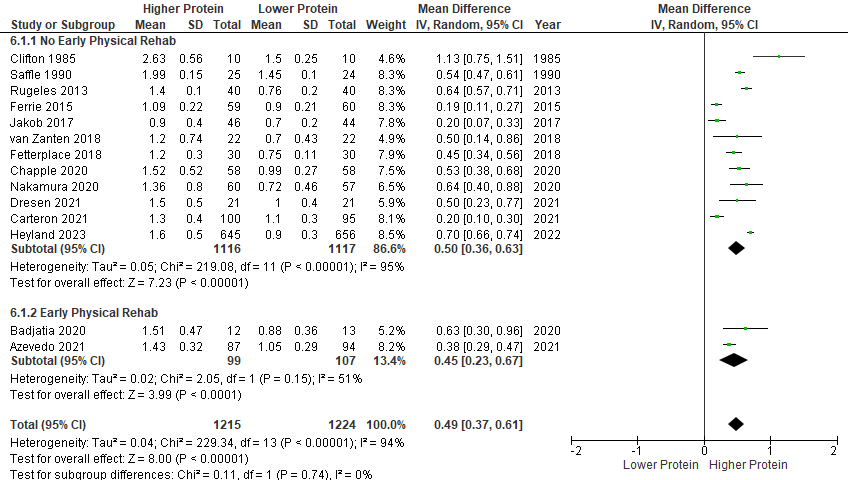  Pooled protein delivered between higher vs lower protein group: 1.49±0.48 vs 0.92±0.30 g/kg/d |
| --- |
| **b) Energy delivered (kcal/kg/day)**  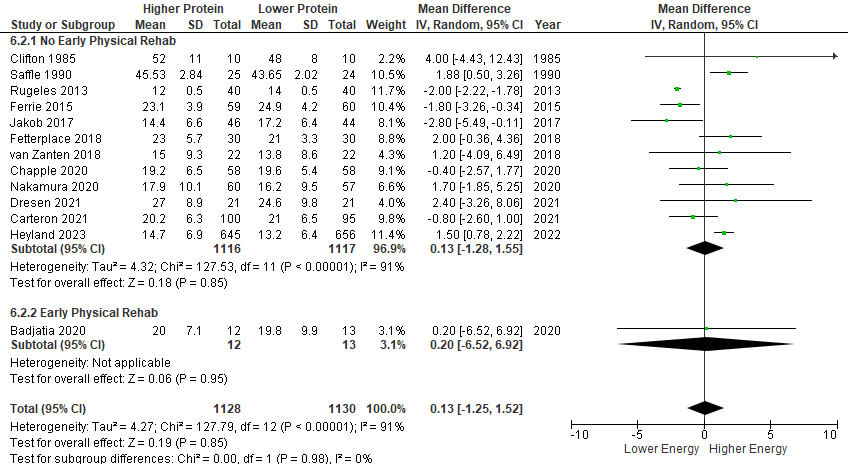  Pooled energy received between higher vs lower protein group: 17.48±6.85 vs 16.60±6.63 kcal/kg/d |

## Figure S3 ROB2 Traffic Light and Summary Plots

### a) Overall Mortality

| **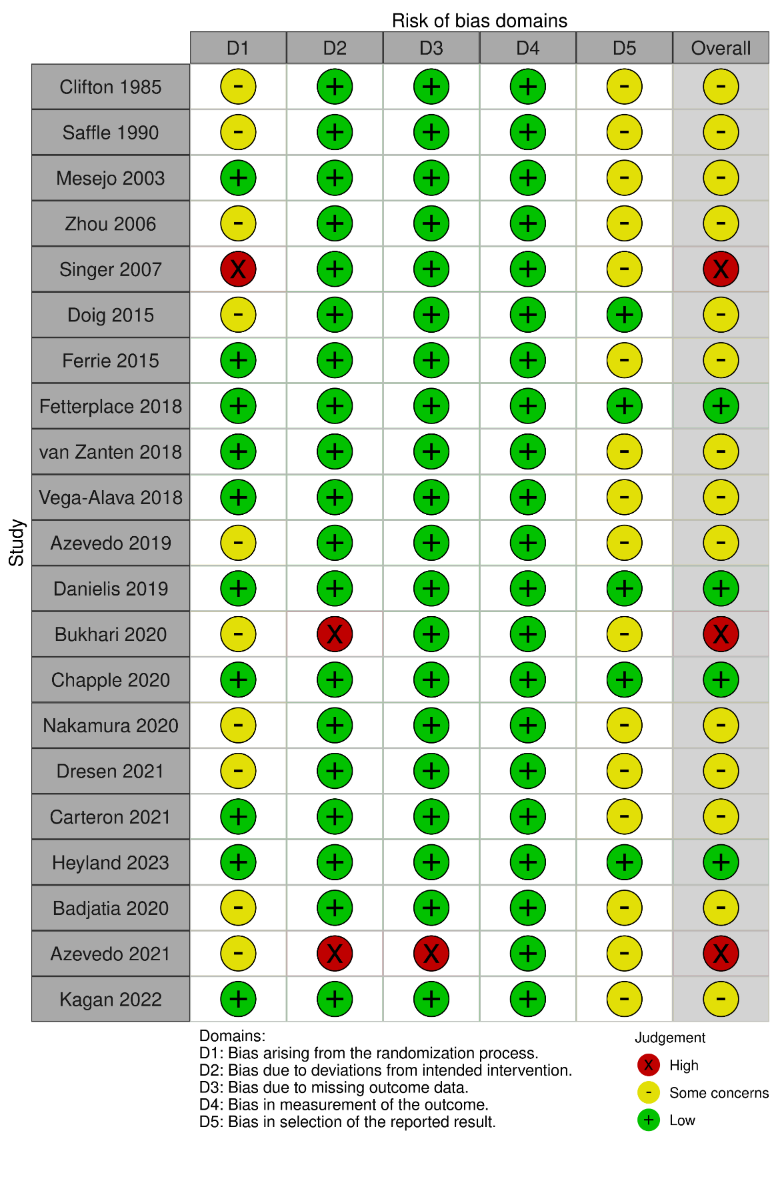** | **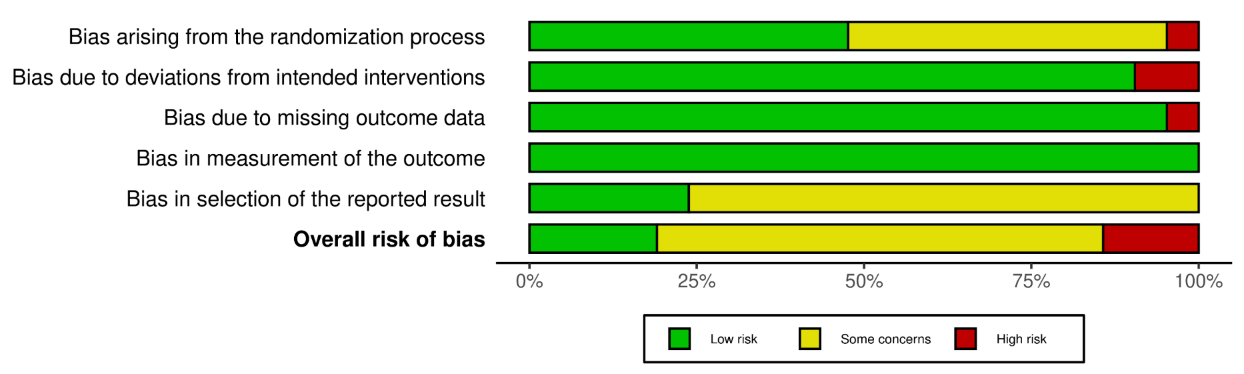** |
| --- | --- |

### b) Infectious Complications

| **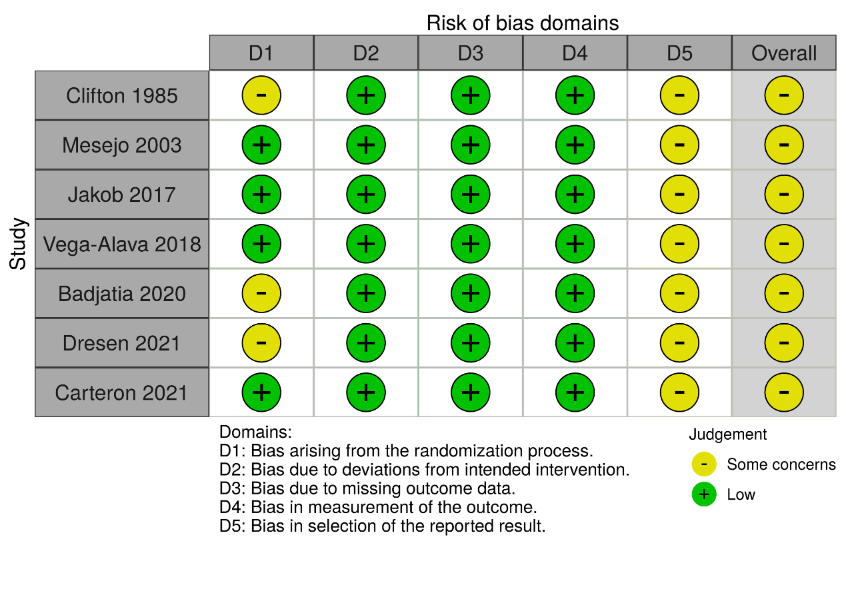** | **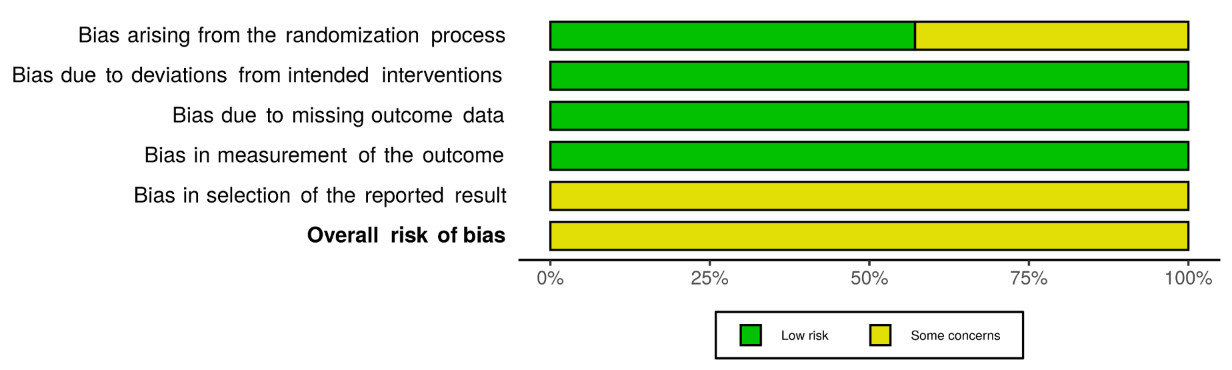** |
| --- | --- |

### c) Duration of mechanical ventilation

| **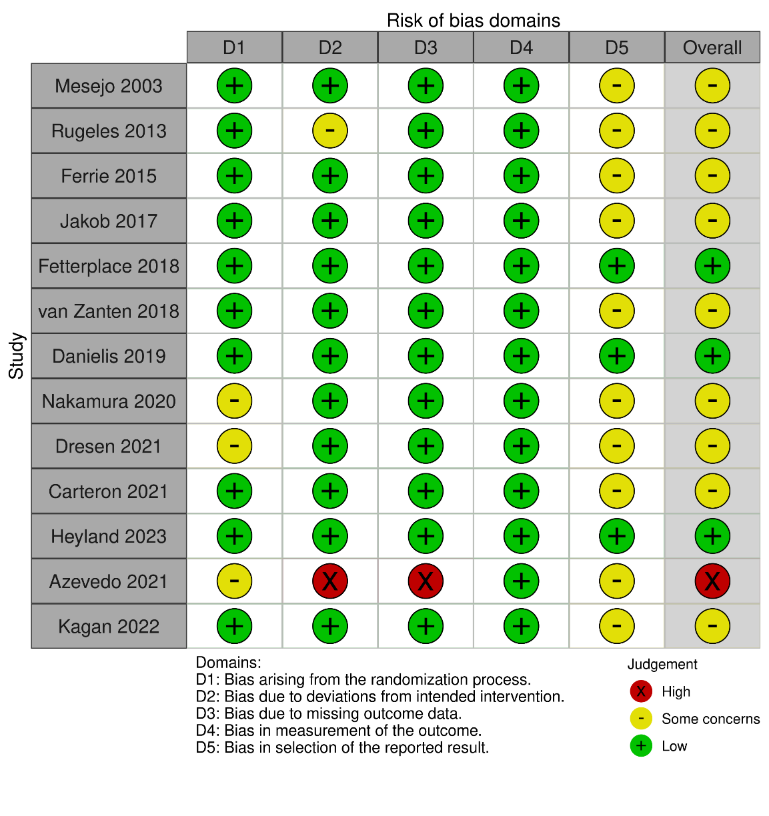** | **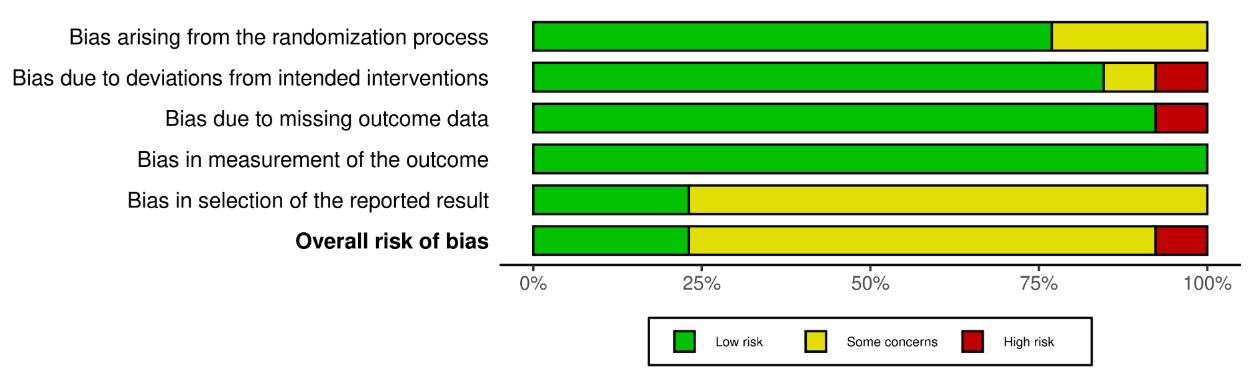** |
| --- | --- |

### d) ICU length of stay

| **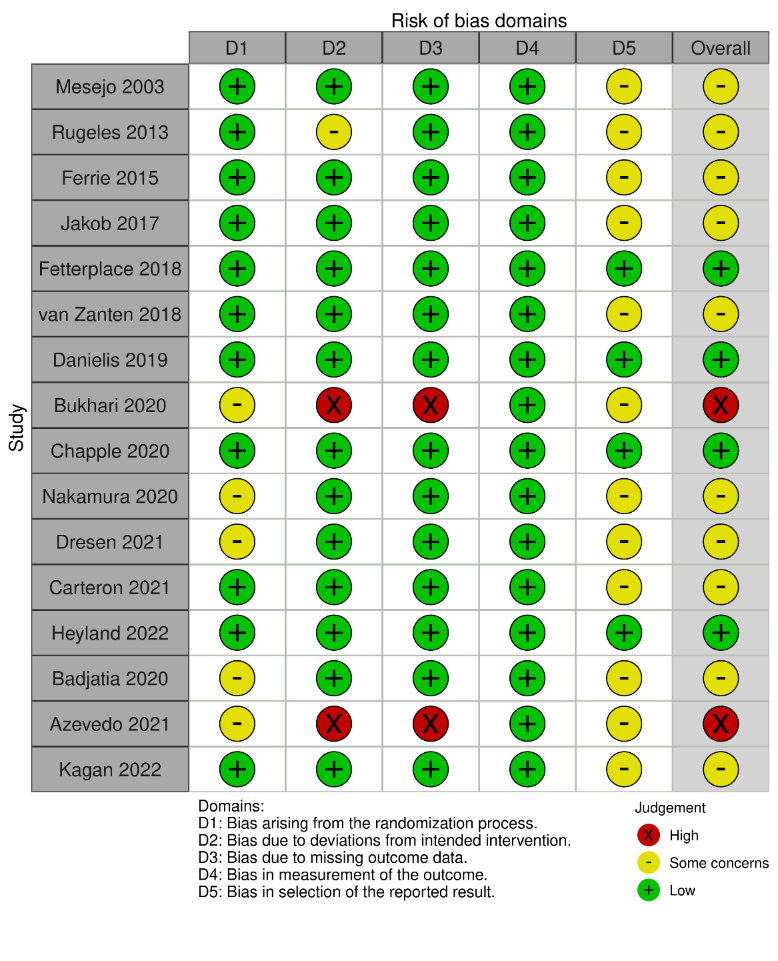** | **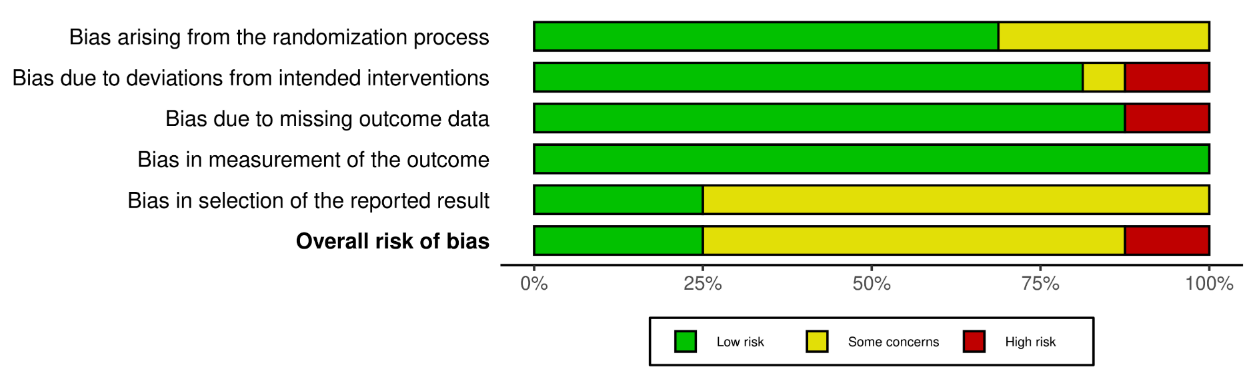** |
| --- | --- |

### e) Hospital length of stay

| **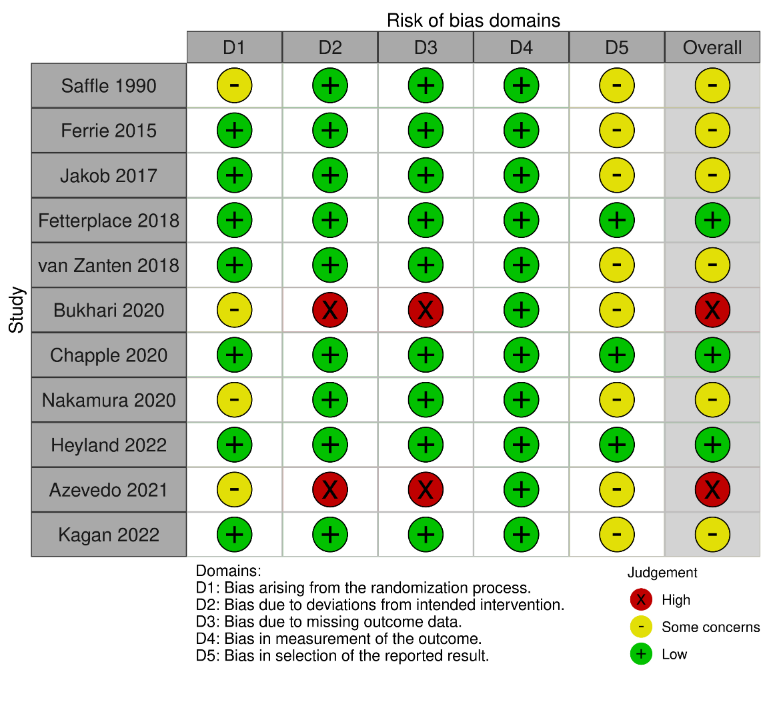** | **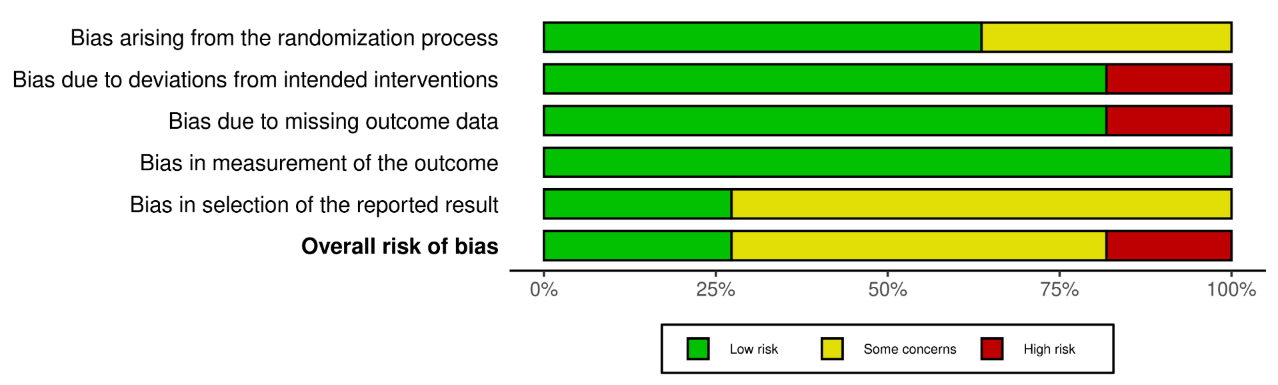** |
| --- | --- |

### f) Muscle mass

| **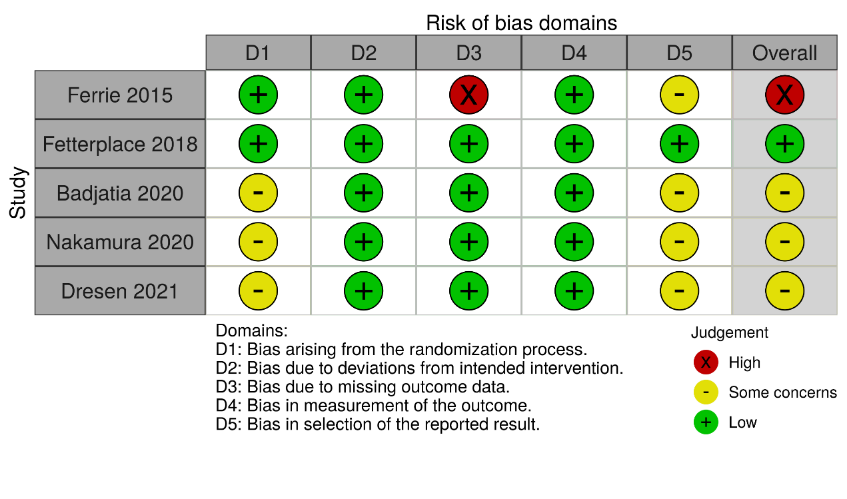** | **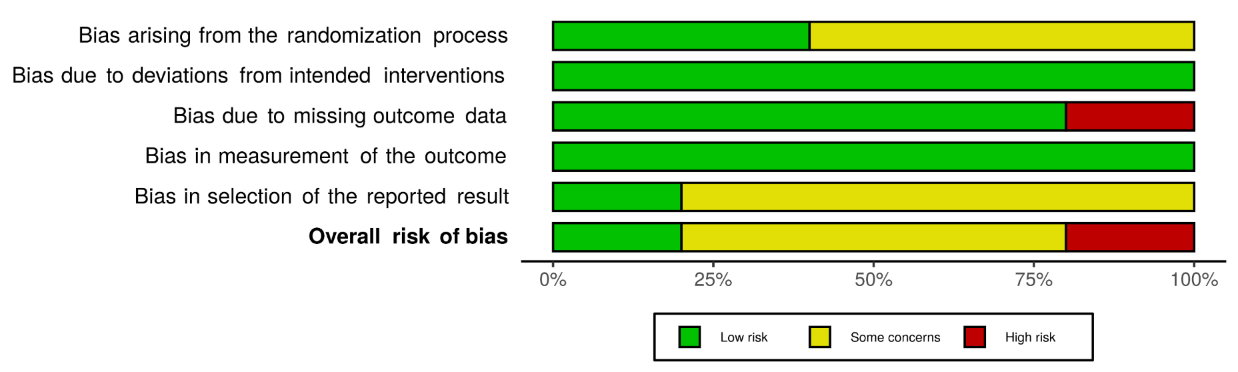** |
| --- | --- |

### g) Muscle strength

| **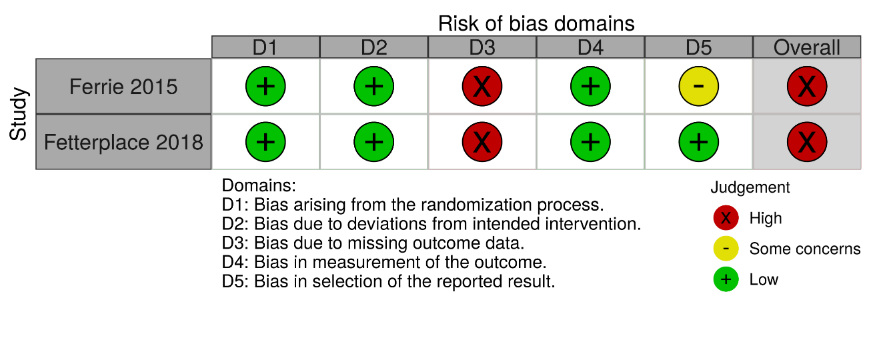** | **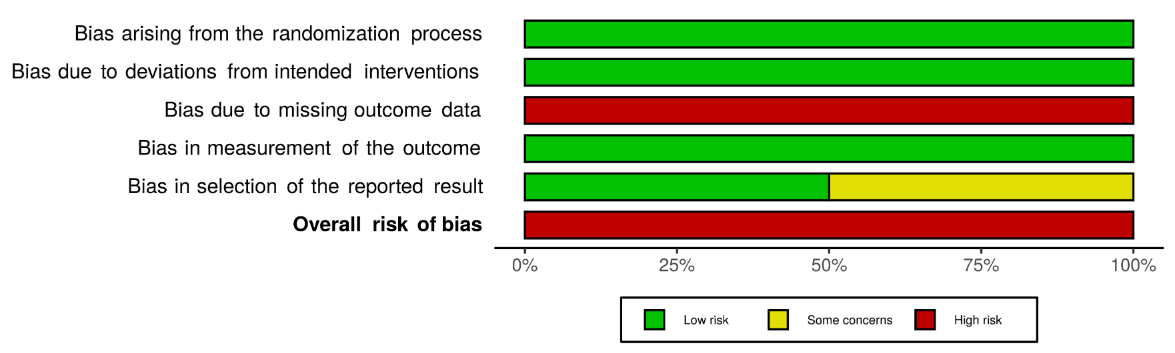** |
| --- | --- |

### h) Discharge to rehab facilities

| **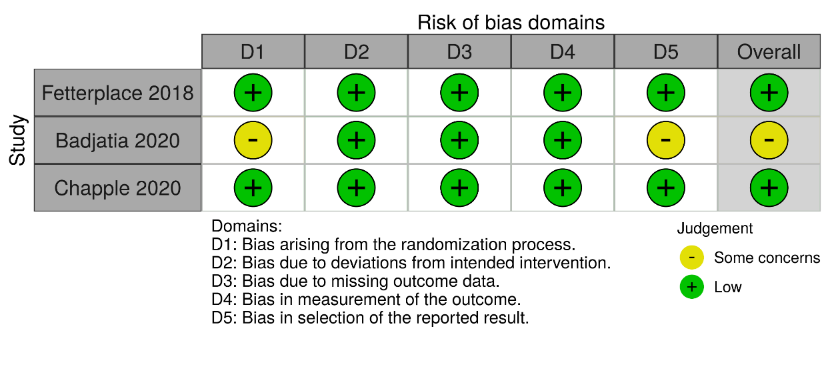** | **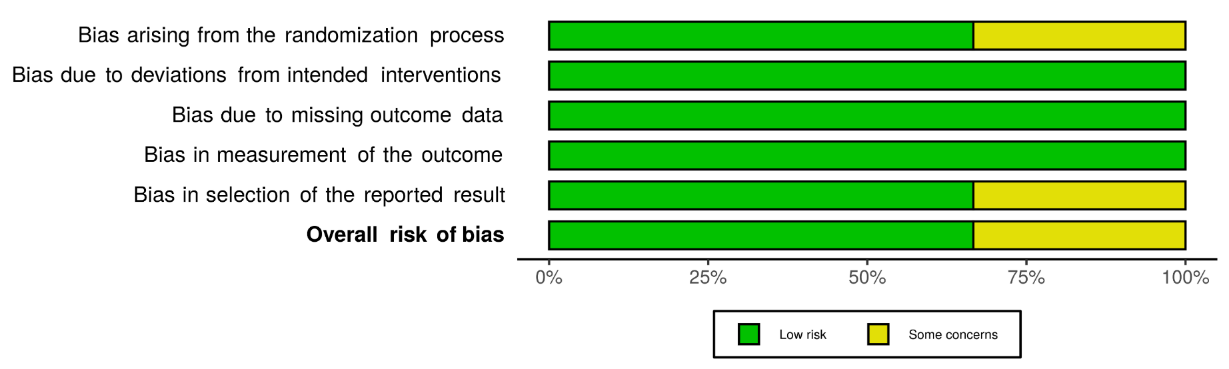** |
| --- | --- |

### i) Self-reported Quality of life physical measures at day 90

| 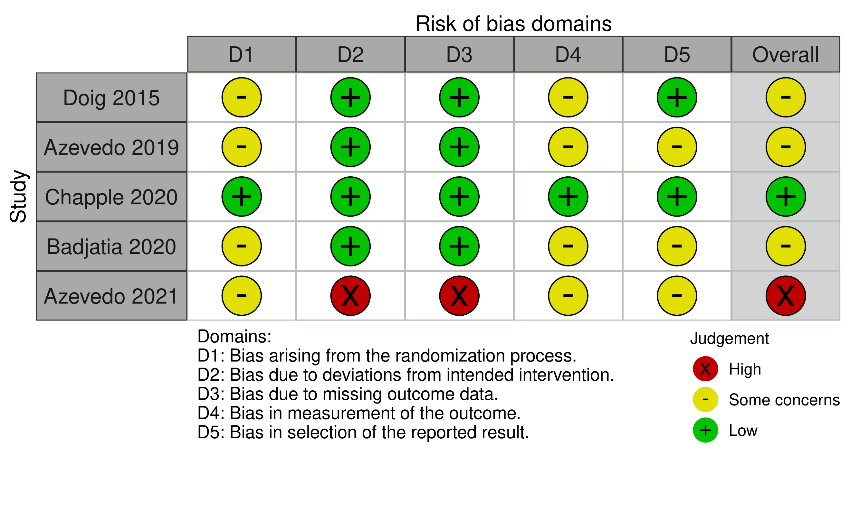 | 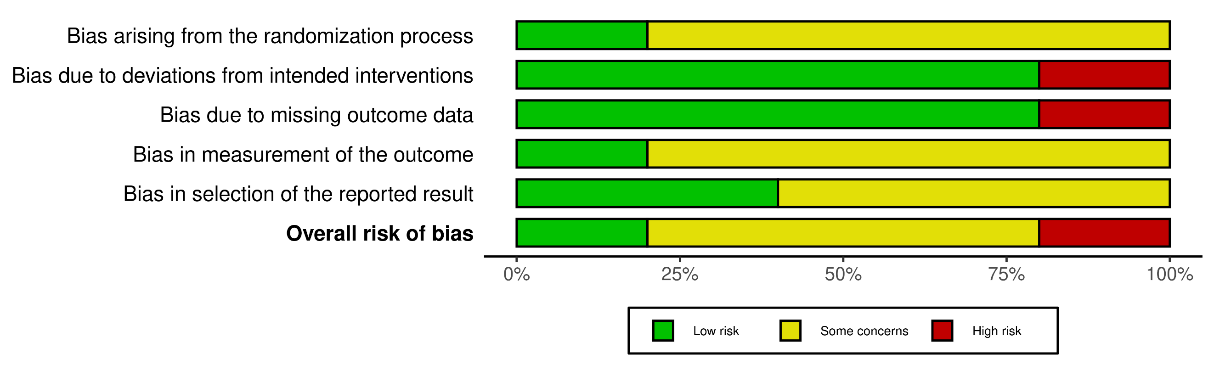 |
| --- | --- |

### j) Incidence of Diarrhea

| **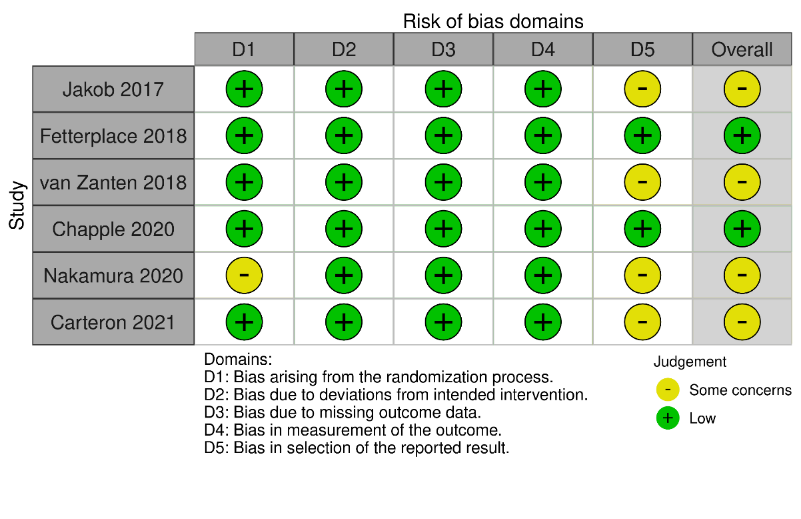** | **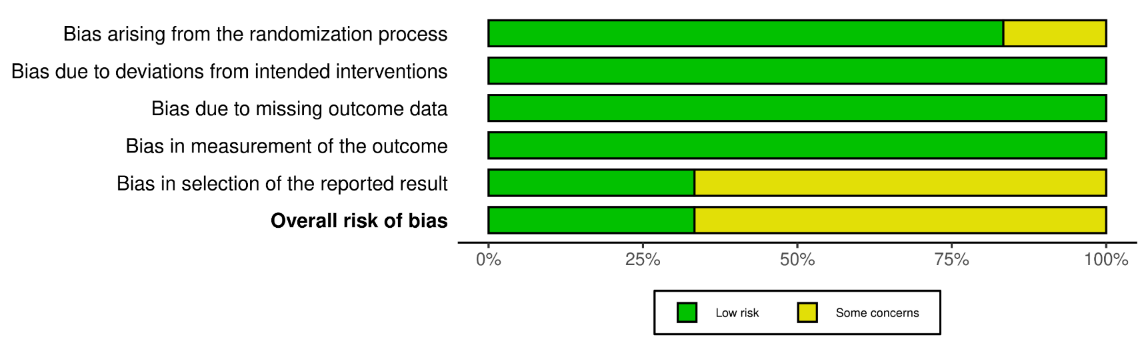** |
| --- | --- |

Figure S4 Funnel plots

a) Overall mortality

**
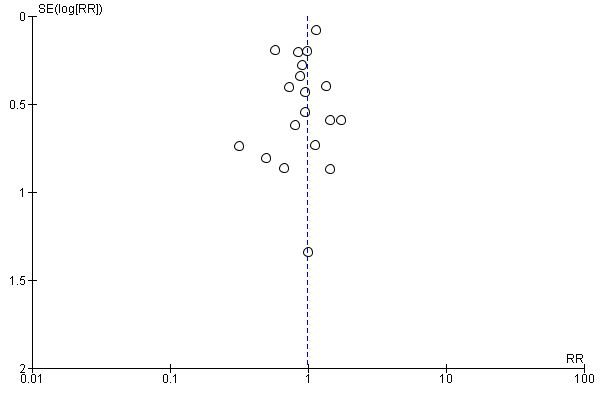
**

Regression-based Egger test for small-study effects

Random-effects model

Method: DerSimonian-Laird

H0: beta1 = 0; no small-study effects

beta1 = -0.35

SE of beta1 = 0.318

z = -1.10

Prob > |z| = 0.2720

b) Infectious complications

**
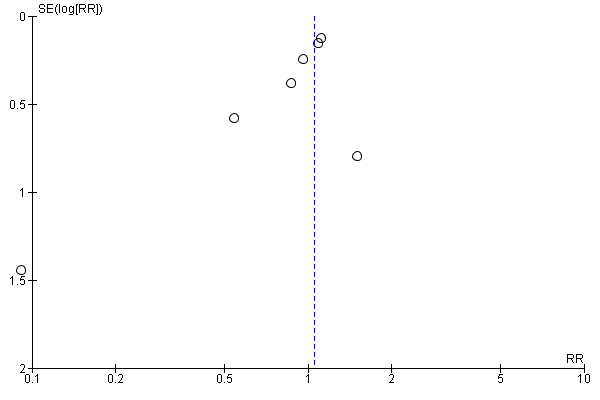
**

c) Duration of mechanical ventilation

**
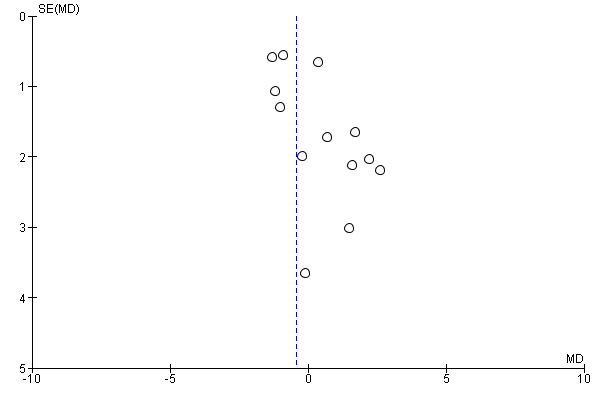
**

Regression-based Egger test for small-study effects

Random-effects model

Method: DerSimonian-Laird

H0: beta1 = 0; no small-study effects

beta1 = 1.13

SE of beta1 = 0.523

z = 2.16

**Prob > |z| = 0.0308**

d) ICU length of stay


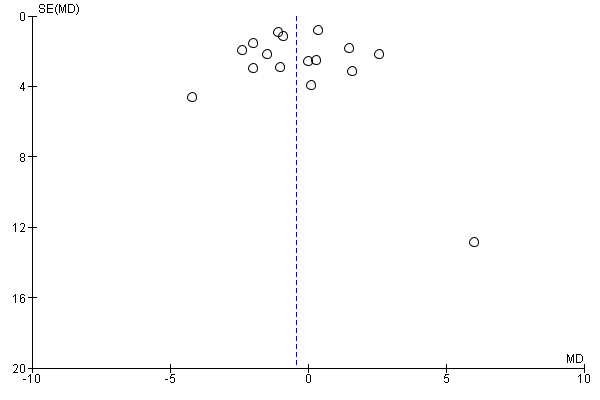


Regression-based Egger test for small-study effects

Random-effects model

Method: DerSimonian-Laird

H0: beta1 = 0; no small-study effects

beta1 = 0.01

SE of beta1 = 0.488

z = 0.03

Prob > |z| = 0.9760

e) Hospital length of stay


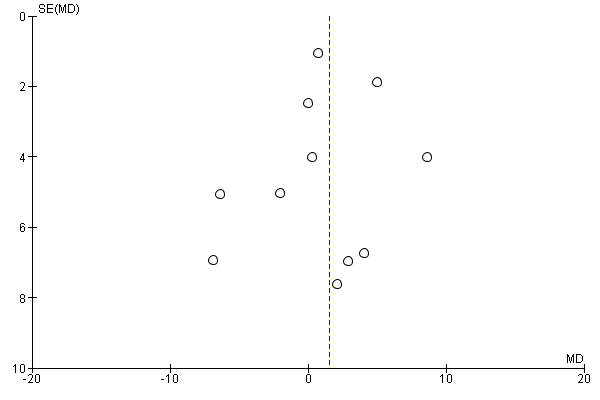


Regression-based Egger test for small-study effects

Random-effects model

Method: DerSimonian-Laird

H0: beta1 = 0; no small-study effects

beta1 = -0.37

SE of beta1 = 0.622

z = -0.59

Prob > |z| = 0.5572

f) Muscle mass

i) Muscle mass (mean difference)
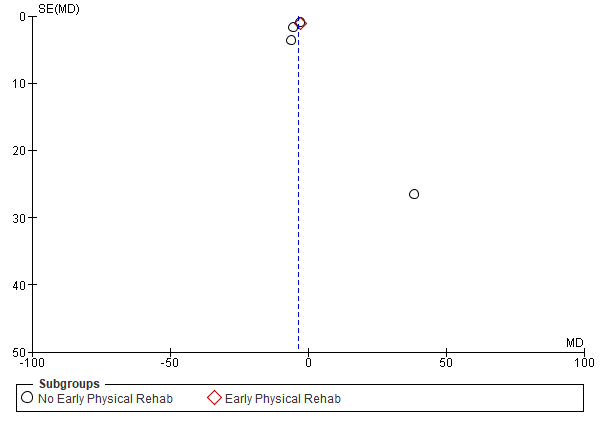


ii) Muscle mass (standardized mean difference)


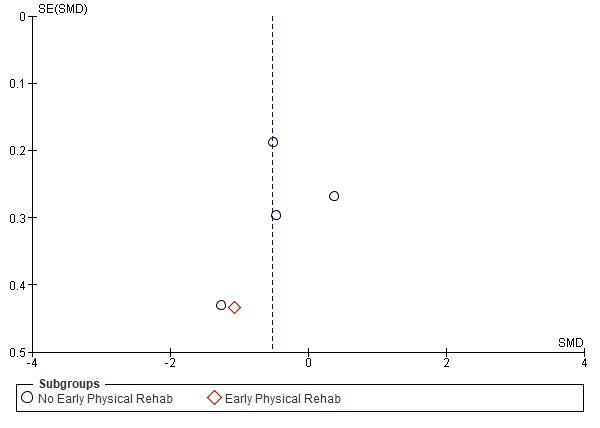


g) Handgrip strength


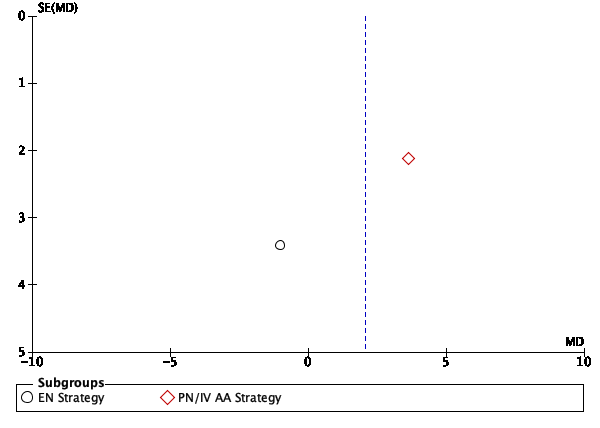


h) Discharge to Rehabilitation Facilities

**
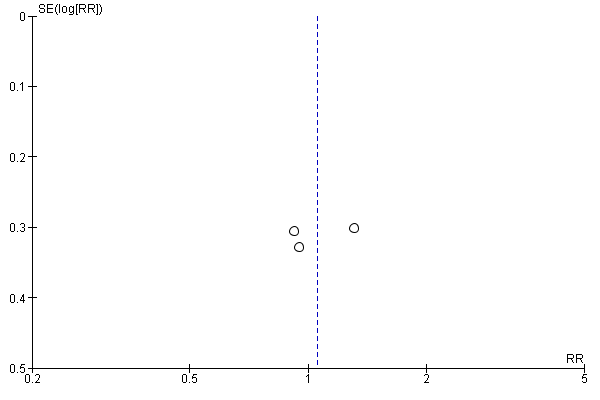
**

i) Self-reported Quality of life Physical Measure at day 90

**
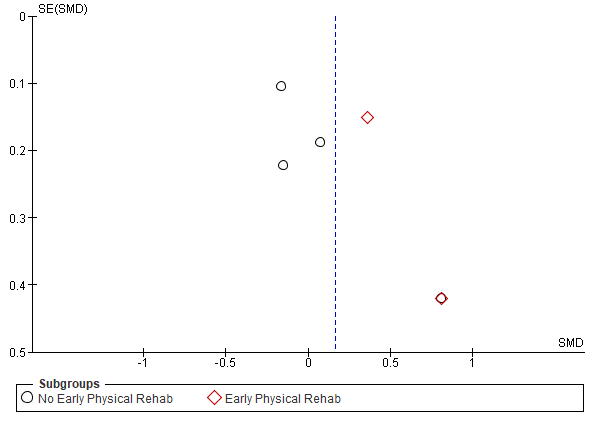
**

j) Incidence of diarrhea**
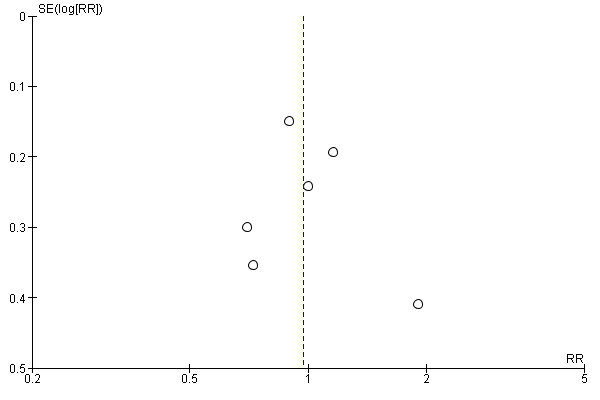
**

## Figure S5 ICU, Hospital, 28- and ≥60-day Mortality and additional AKI subgroup analysis

| a) ICU Mortality **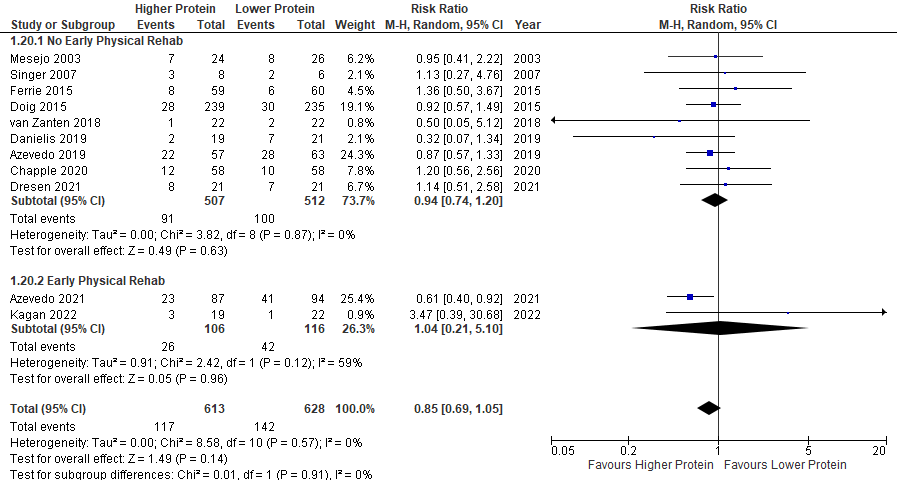** |
| --- |
| b) Hospital Mortality **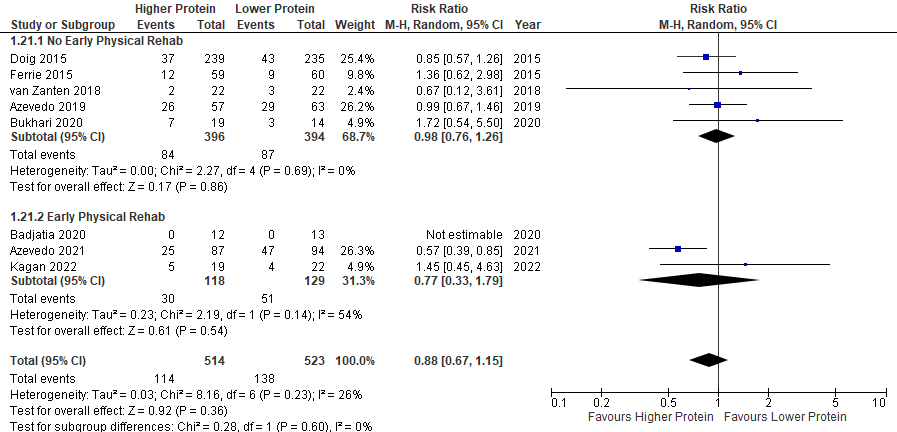** |

| c) 28-day Mortality **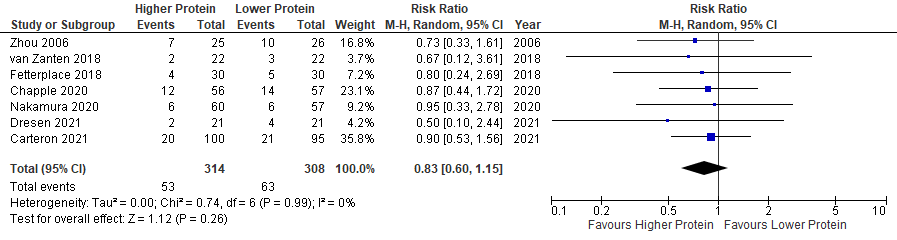** |
| --- |
| d) ≥60-day Mortality **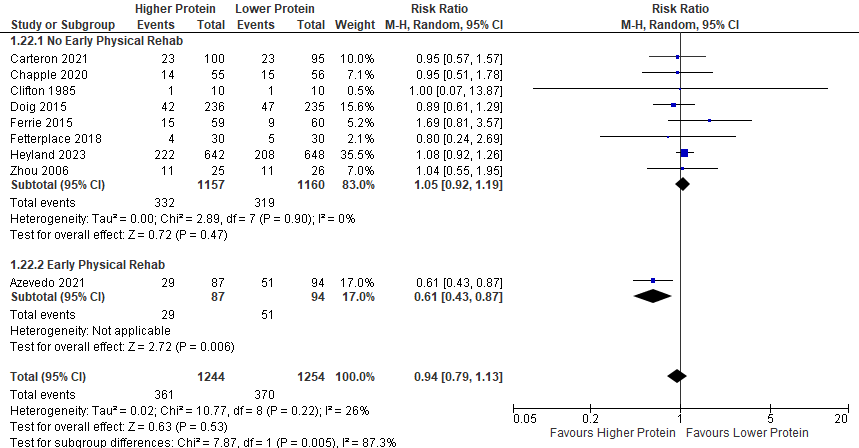** |
| e) Additional AKI subgroup analysis (risk difference) 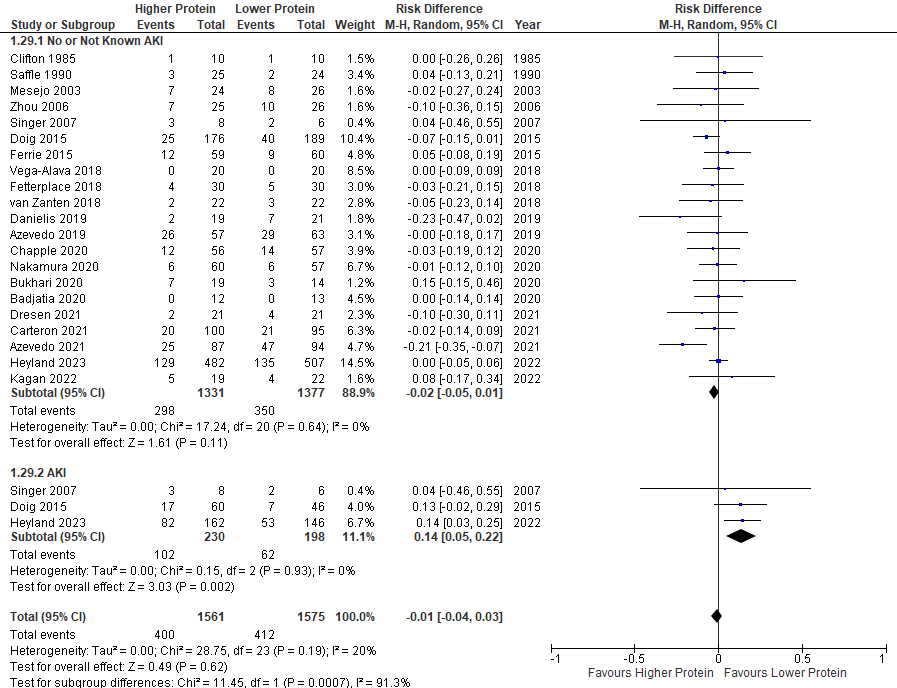  **Number needed to harm = 1/0.14 = 7.15** |

## Figure S6: Biochemical Outcomes

### a) Serum urea (mmol/L)


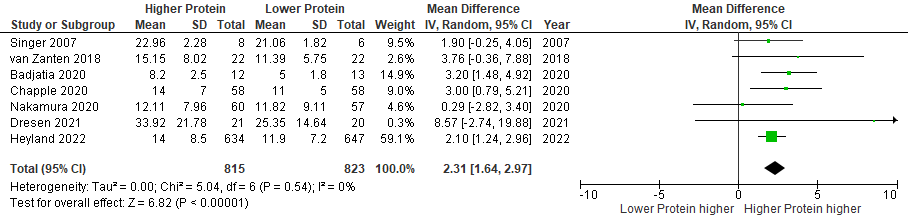


### b) Urinary Urea Nitrogen (g)


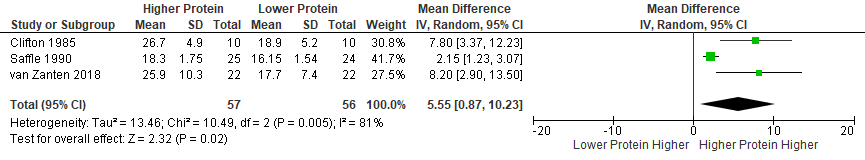


### c) Nitrogen balance (g) – Ferrie D7


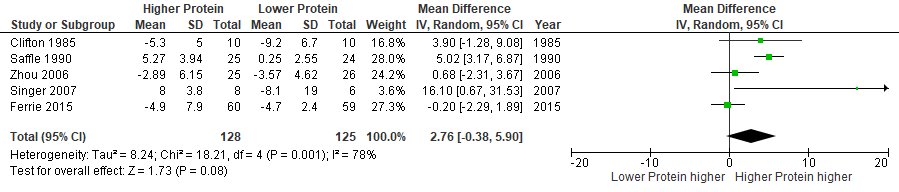


### d) Nitrogen balance (g) – Ferrie D3


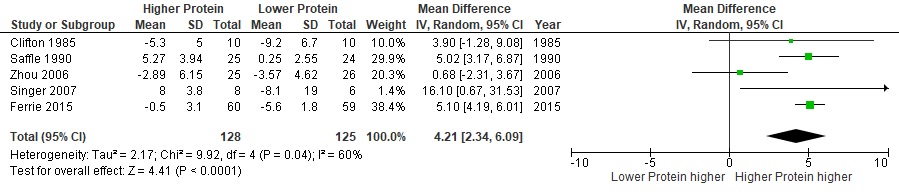


### e) Serum Creatinine (umol/L)


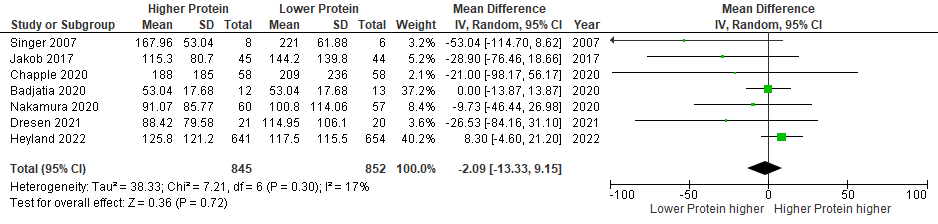


### f) Blood glucose (mmol/L)


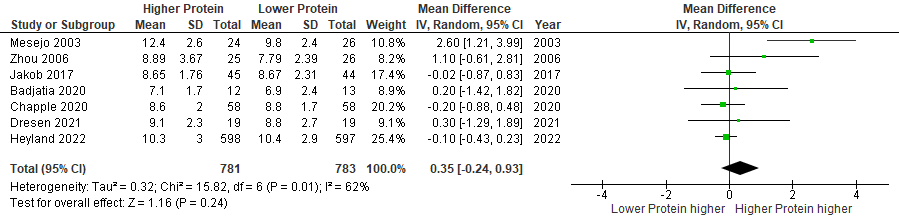


### g) Insulin (unit)


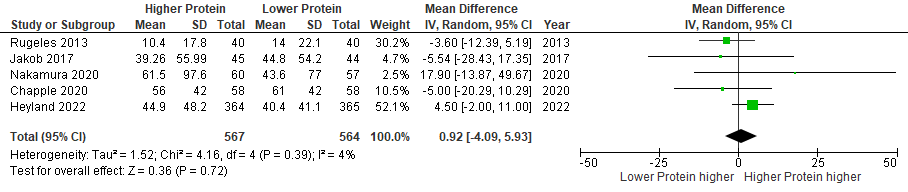


### h) Serum albumin (g/L)


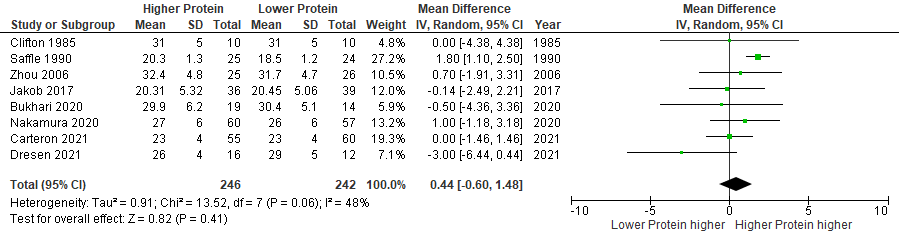


### i) Prealbumin (mg/dL)


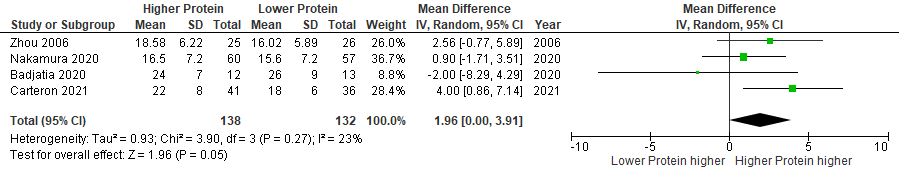


### j) Hemoglobin (g/L)

**
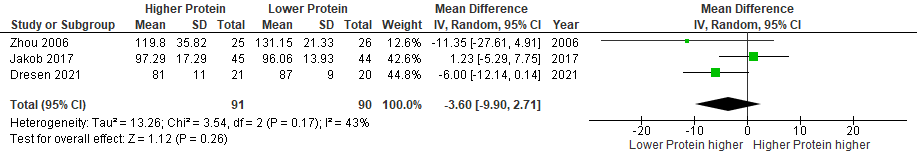
**

### k) White blood cells (10^9^/L)


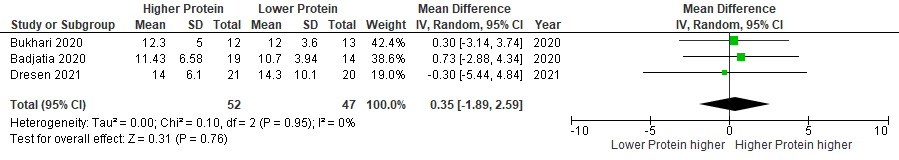


### l) Lymphocyte count (cells per µL of blood)


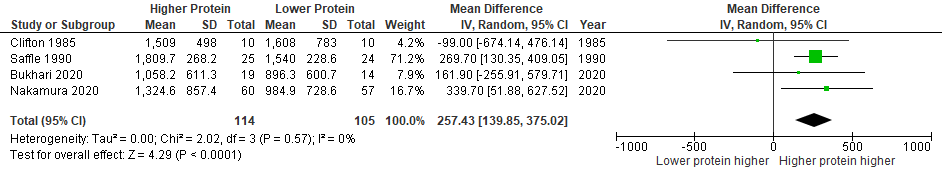


### m) C-reactive protein (mg/dL)


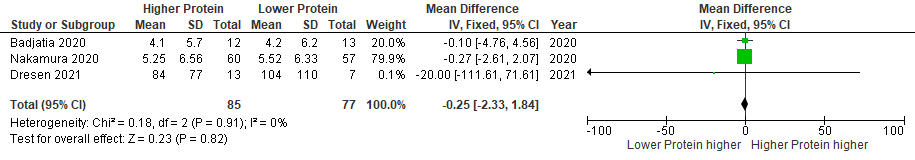


### n) Interleukin 6 (ng/L)


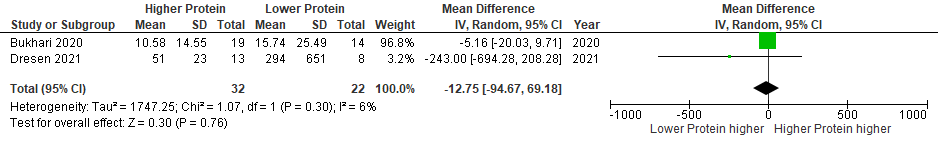


### o) Phosphate level (mmol/L)


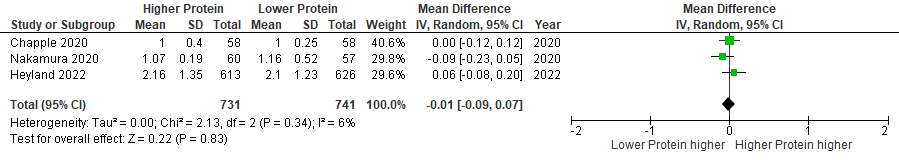


### p) Triglycerides (mmol/L)


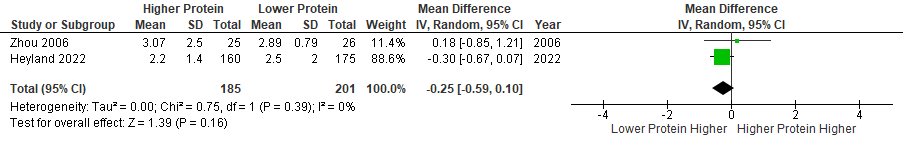


## Figure S7 Subgroup analysis (low vs other ROB)

### a) Overall mortality

**
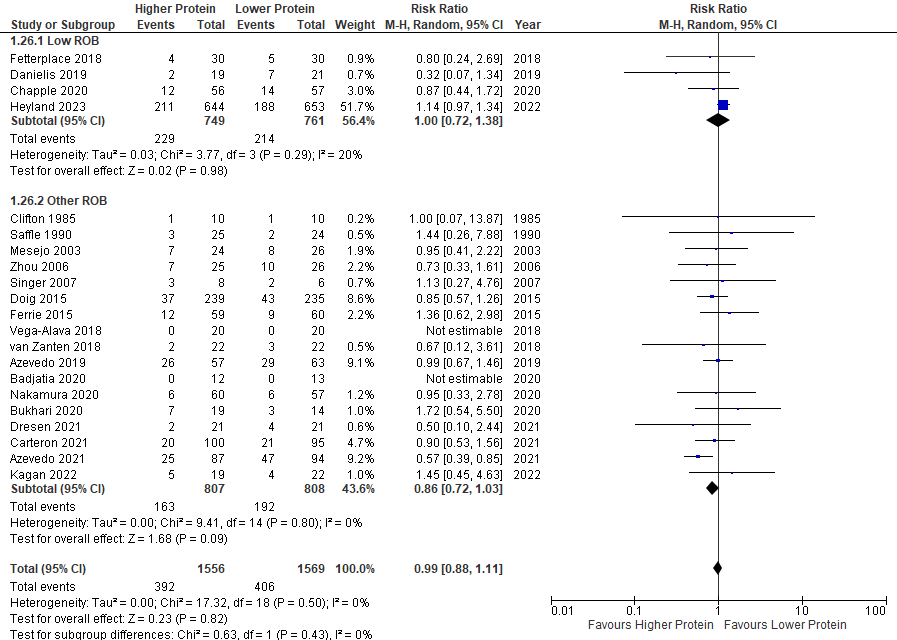
**

### b) Infectious complications (all studies had some concerns)

### c) ICU LOS

**
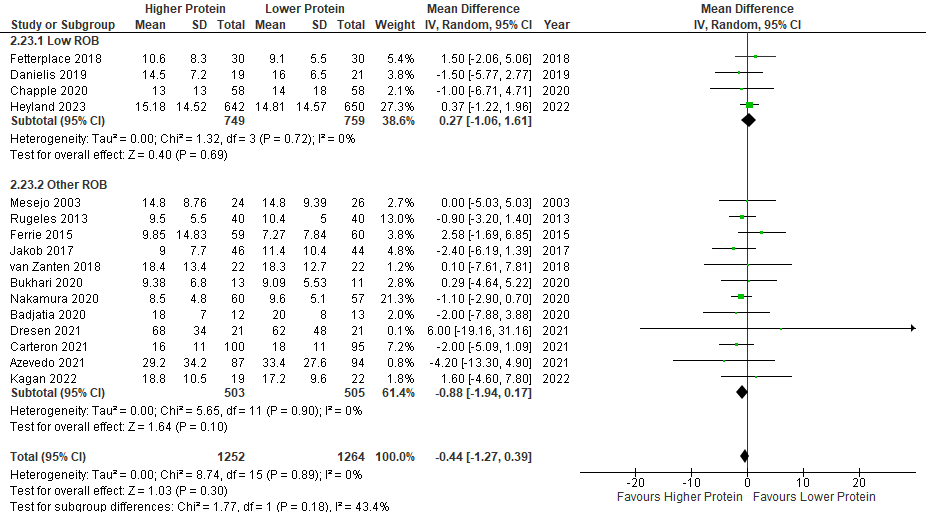
**

### d) Hospital LOS

**
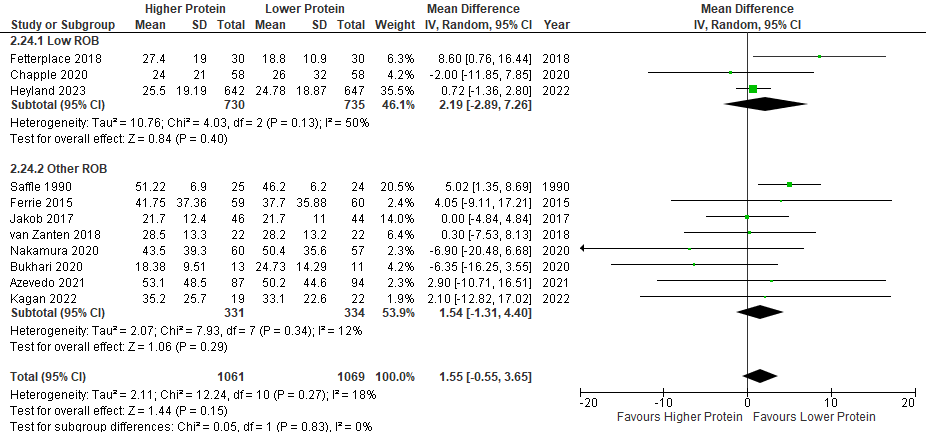
**

### e) Length of mechanical ventilation

**
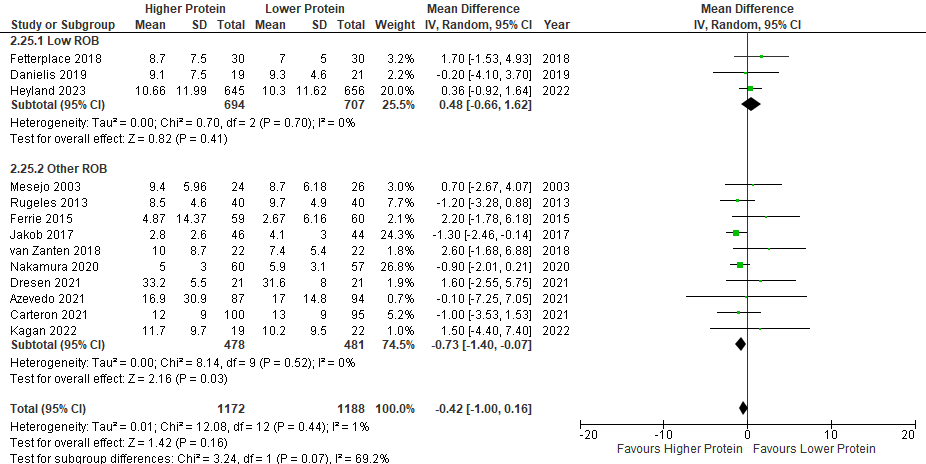
**

### f) Muscle wasting per week


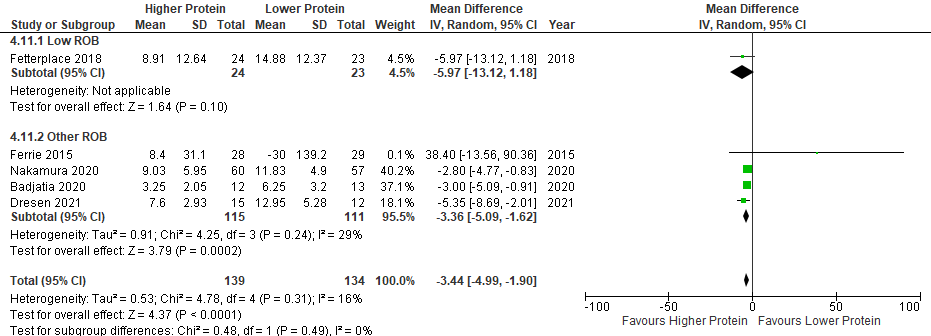


### g) Handgrip strength (all studies were high risk of bias)

### h) Discharge to rehabilitation facilities


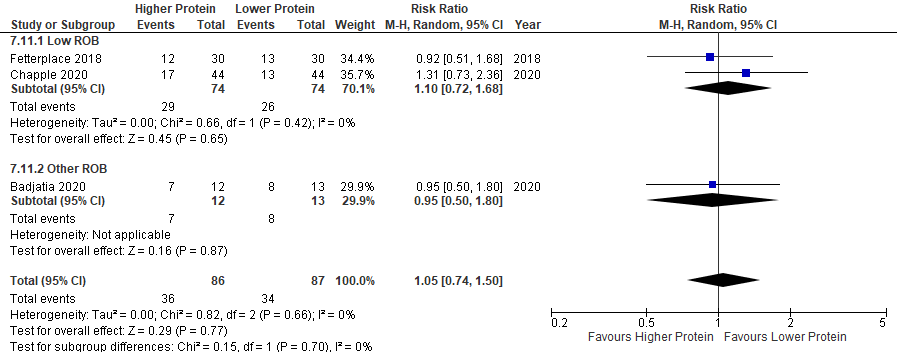


### i) Self-reported Quality of life physical function at day 90


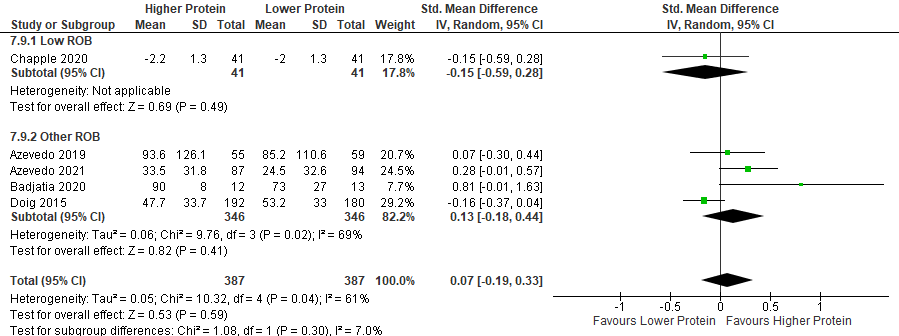


### j) Incidence of Diarrhea


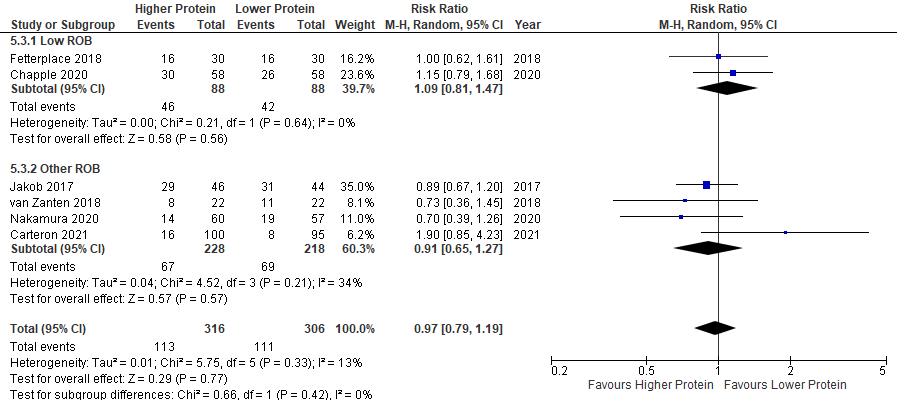


## Figure S8 Subgroup analysis (single vs multicenter studies)

### a) Overall mortality


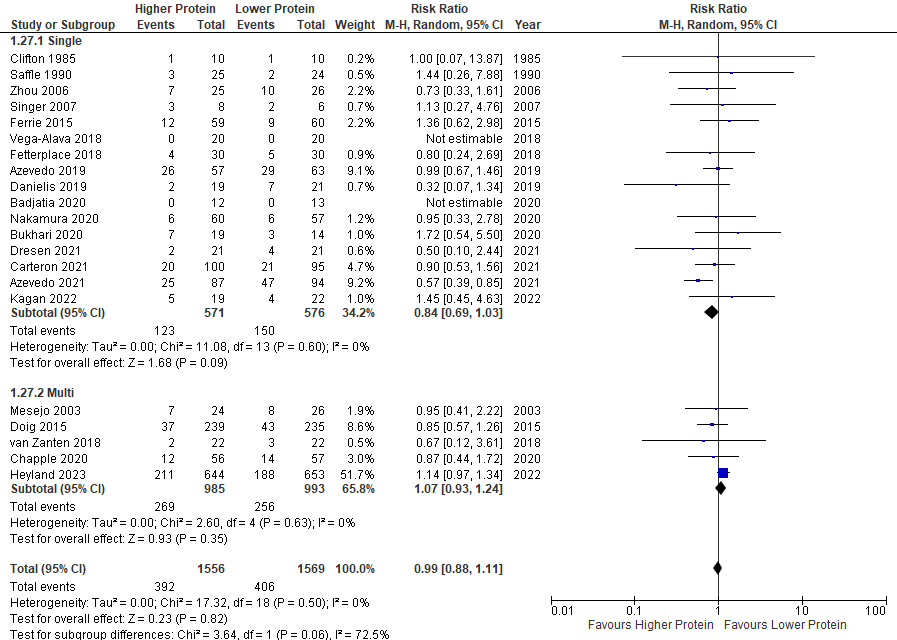


### b) Infectious complications (no multicenter studies reported this outcome)

### c) Duration of mechanical ventilation


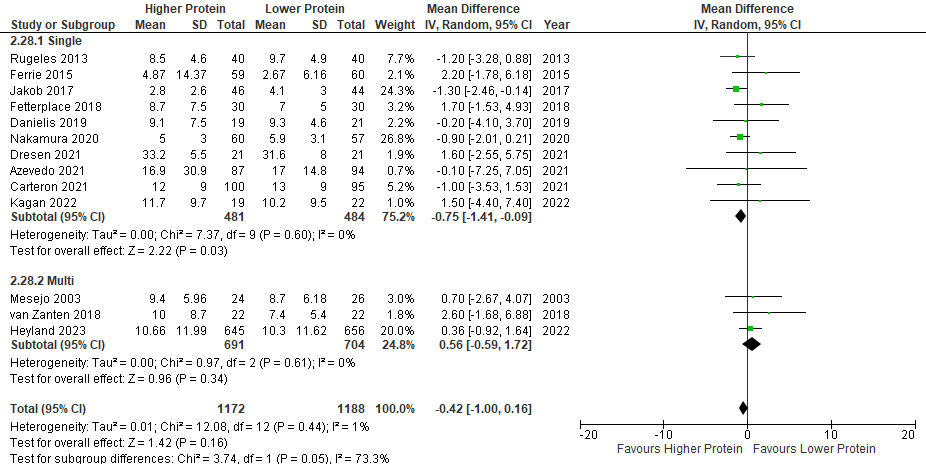


### d) ICU length of stay

**
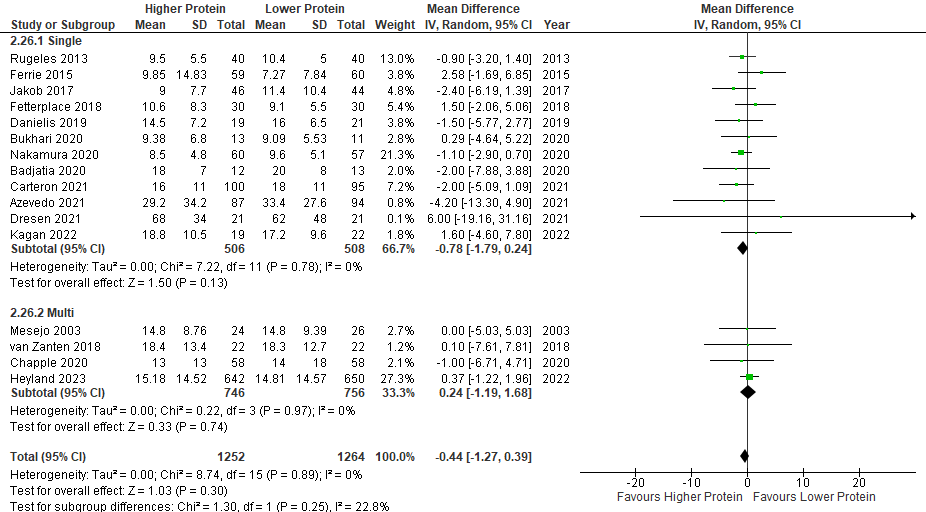
**

### e) Hospital length of stay

**
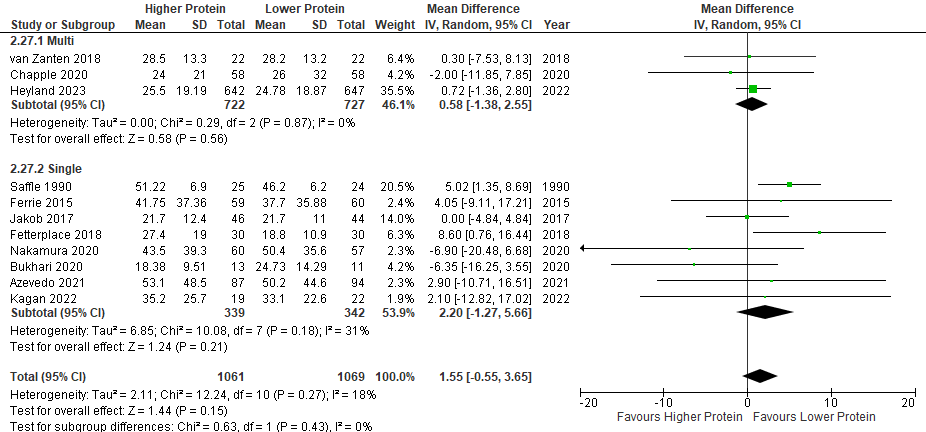
**

### f) Muscle mass (no multicenter studies reported this outcome)

### g) Handgrip strength (no multicenter studies reported this outcome)

### h) Discharge to rehabilitation facilities

**
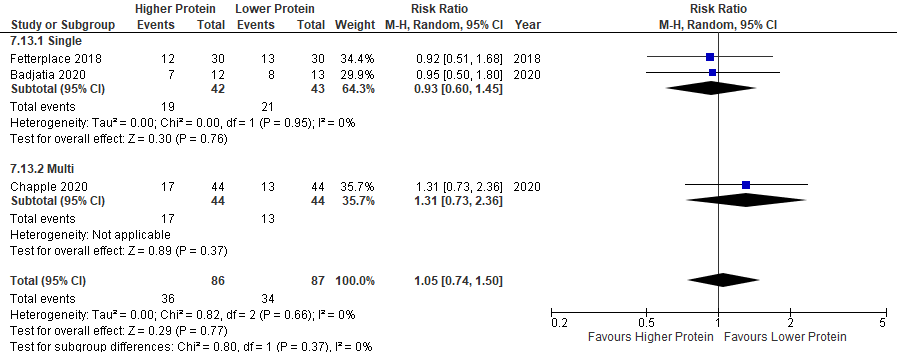
**

### i) Self-reported Quality of life Physical Measure at day 90

**
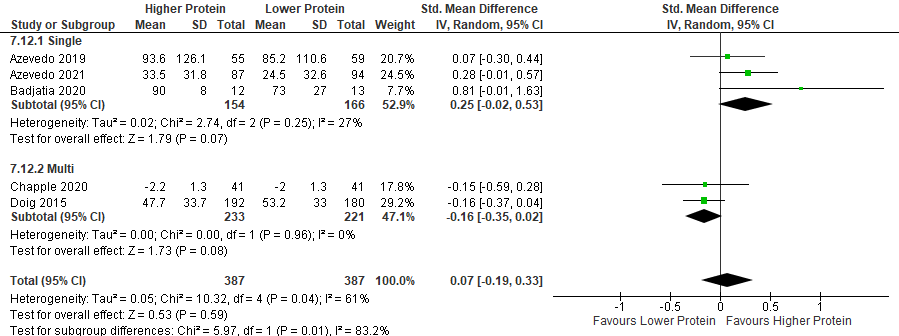
**

### j) Incidence of diarrhea

**
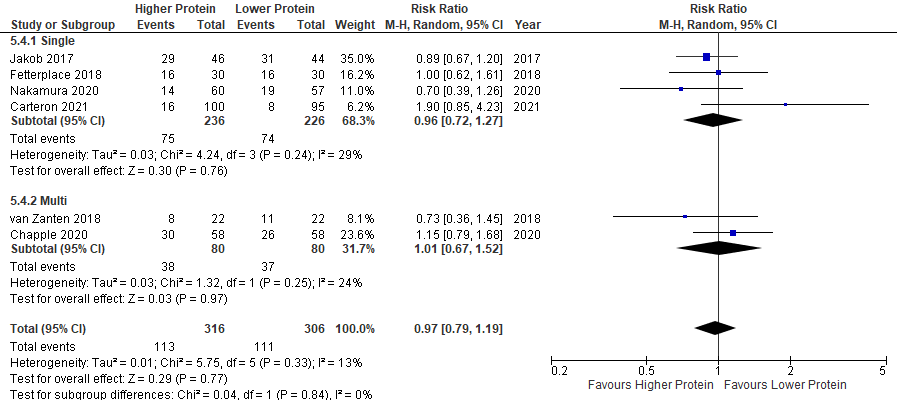
**

## Figure S9 Trial sequential analysis graphs (other outcomes)

### a) Muscle wasting per week (5 studies, n=273)


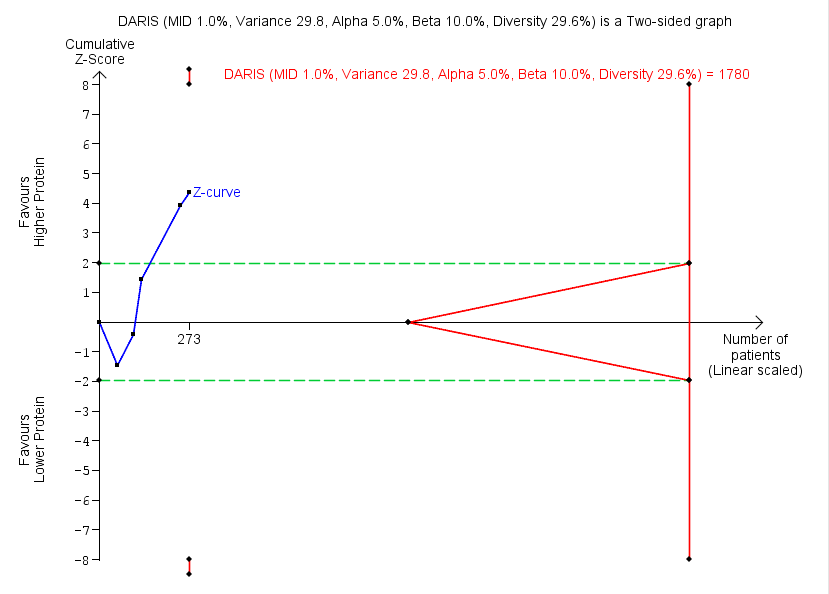


TSA adjusted mean difference (95% confidence interval): -3.44 (-9.74, 2.86)

TSA adjusted mean difference (95% confidence interval): 2.08 (-4.34, 8.50)

### b) Handgrip strength (2 studies, n=130)

**
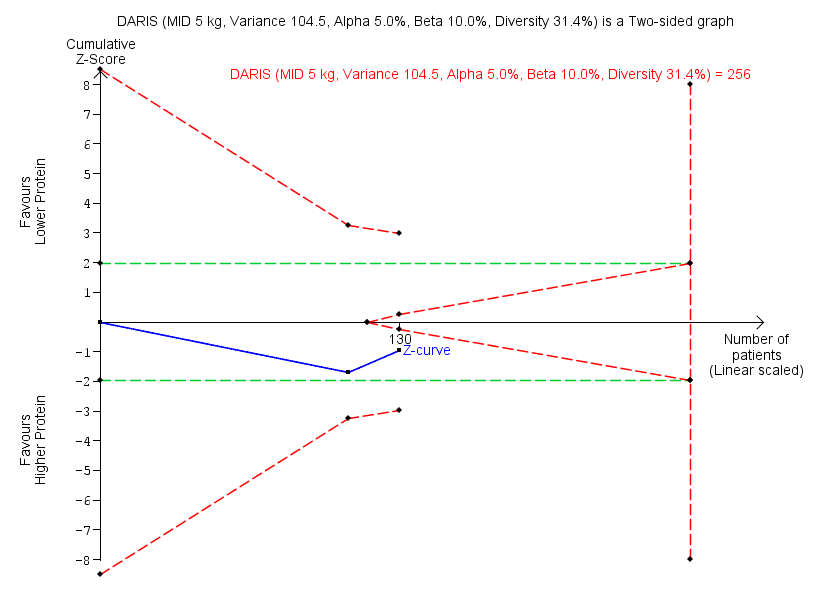
**

TSA adjusted mean difference (95% confidence interval): -3.44 (-9.74, 2.86)

### c) Incidence of diarrhoea (6 studies, n=662)


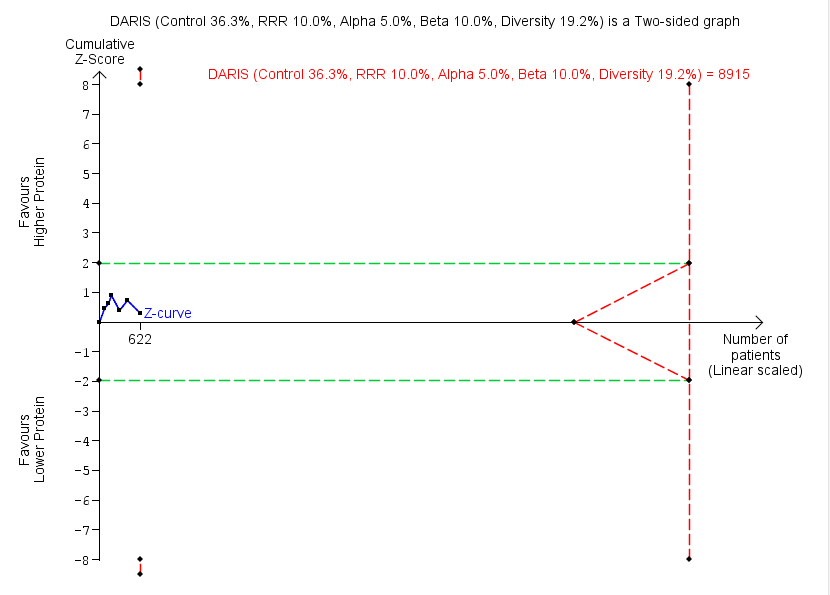


**TSA adjusted mean difference (95% confidence interval): 0.97 (0.42, 2.23)**

TSA was analyzed using DerSimonian and Laird random-effects model. The Z curve in blue measures the treatment effect (pooled relative risk). The parallel lines in green are the boundaries of conventional meta-analysis (alpha 5%), and the boundaries of benefit and harm are boundaries of conventional meta-analysis adjusted for between-trial heterogeneity and multiple statistical testing (TSA boundaries). A treatment effect outside the TSA boundaries of benefit/ harm indicates reliable evidence for a treatment effect, and a treatment effect within the futility zone (the triangle between the parallel lines) indicates that there is reliable evidence of no treatment effect.

DARIS: Diversity adjusted required information size is the calculated optimum sample size for statistical inference, MID: Minimally important difference, RRR: Relative risk reduction, TSA: Trial sequential analysis

## References

1. Arsham H. Pooling the Means, and Variances. https://home.ubalt.edu/ntsbarsh/business-stat/otherapplets/Pooled.htm

2. Bukhari A, Taslim NA, As’ad S, et al. Comparison of different early enteral feeding formulas on critically ill patients. *J Nutr Sci Vitaminol*. 2020;66:S2-S10. doi:10.3177/jnsv.66.S2

3. Saffle J, Larson C, Sullivan J. A randomized trial of indirect calorimetry-based feedings in thermal injury. *J Trauma*. 1990;30(7):776-782. doi:10.1097/00005373-199007000-00003

4. Imberger G, Thorlund K, Gluud C, Wetterslev J. False-positive findings in Cochrane meta-analyses with and without application of trial sequential analysis: an empirical review. *BMJ Open*. 2016;6(e011890). doi:10.1136/bmjopen-2016

5. Lee ZY, Yap CSL, Hasan MS, et al. The effect of higher versus lower protein delivery in critically ill patients: a systematic review and meta-analysis of randomized controlled trials. *Crit Care*. 2021;25(1). doi:10.1186/s13054-021-03693-4

6. Doig GS, Simpson F, Bellomo R, et al. Intravenous amino acid therapy for kidney function in critically ill patients: a randomized controlled trial. *Intensive Care Med*. 2015;41(7):1197-1208. doi:10.1007/s00134-015-3827-9

7. Lee ZY, Ong SP, Ng CC, et al. Association between ultrasound quadriceps muscle status with premorbid functional status and 60-day mortality in mechanically ventilated critically ill patient: A single-center prospective observational study. *Clin Nutr*. 2021;40(3):1338-1347. doi:10.1016/j.clnu.2020.08.022

8. Bobos P, Nazari G, Lu Z, MacDermid JC. Measurement Properties of the Hand Grip Strength Assessment: A Systematic Review With Meta-analysis. *Arch Phys Med Rehabil*. 2020;101(3):553-565. doi:10.1016/j.apmr.2019.10.183

9. Clifton GL, Robertson CS, Contant CF. Enteral hyperalimentation in head injury. *J Neurosurg*. 1985;62:186-193. doi:10.3171/jns.1985.62.2.0186

10. Mesejo A, Acosta JA, Ortega C, et al. Comparison of a high-protein disease-specific enteral formula with a high-protein enteral formula in hyperglycemic critically ill patients. *Clin Nutr*. 2003;22(3):295-305. doi:10.1016/S0261-5614(02)00234-0

11. Zhou CP, Su Y ying. Effect of the Equal Non-protein-calorie but Different Protein Intake on Enteral Nutritional Metabolism in 51 Patients with Severe Stroke. A Randomized Controlled Study. *Chinese J Clin Nutr*. 2006;14(6):351-355.

12. Singer P. High-dose amino acid infusion preserves diuresis and improves nitrogen balance in non-oliguric acute renal failure. *Wien Klin Wochenschr*. 2007;119(7-8):218-222. doi:10.1007/s00508-007-0794-3

13. Rugeles SJ, Rueda JD, Díaz CE, Rosselli D. Hyperproteic hypocaloric enteral nutrition in the critically ill patient: A randomized controlled clinical trial. *Indian J Crit Care Med*. 2013;17(6):343-349. doi:10.4103/0972?5229.123438

14. Ferrie S, Allman-Farinelli M, Daley M, Smith K. Protein requirements in the critically ill: a randomised controlled trial using parenteral nutrition. *JPEN J Parenter Enteral Nutr*. 2016;40(6):795-805. doi:10.1177/0148607115618449

15. Jakob SM, Butikofer L, Berger D, Coslovsky M, Takala J. A randomized controlled pilot study to evaluate the effect of an enteral formulation designed to improve gastrointestinal tolerance in the critically ill patient-the SPIRIT trial. *Crit Care*. 2017;21:140. doi:10.1186/s13054-017-1730-1

16. Fetterplace K, Deane AM, Tierney A, et al. Targeted Full Energy and Protein Delivery in Critically Ill Patients: A Pilot Randomized Controlled Trial (FEED Trial). *JPEN J Parenter Enteral Nutr*. 2018;42(8):1252-1262. doi:10.1002/jpen.1166

17. van Zanten ARH, Petit L, De Waele J, et al. Very high intact-protein formula successfully provides protein intake according to nutritional recommendations in overweight critically ill patients: a double-blind randomized trial. *Crit Care*. 2018;22(156):1-12.

18. Vega-Alava KM, Sy RAG, Domado AM. The effect of whey protein supplementation on duration of mechanical ventilation: A pilot study. *Philipp J Intern Med*. 2018;56(2):71-76.

19. Azevedo JRA de, Lima HCM, Montenegro WS, et al. Optimized calorie and high protein intake versus recommended caloric-protein intake in critically ill patients: a prospective, randomized, controlled phase II clinical trial. *Rev Bras Ter Intensiva*. 2019;31(2):171-179. doi:10.5935/0103-507X.20190025

20. Danielis M, Lorenzoni G, Azzolina D, et al. Effect of Protein-Fortified Diet on Nitrogen Balance in Critically Ill Patients: Results from the OPINiB Trial. *Nutrients*. 2019;11(5):972. doi:10.3390/nu11050972

21. Badjatia N, Sanchez S, Judd G, et al. Neuromuscular Electrical Stimulation and High‑Protein Supplementation After Subarachnoid Hemorrhage: A Single‑Center Phase 2 Randomized Clinical Trial. *Neurocrit Care*. Published online 2020. doi:10.1007/s12028-020-01138-4

22. Chapple L anne S, Summers MJ, Bellomo R, et al. Use of a High Protein Enteral Nutrition Formula to Increase Protein Delivery to Critically Ill Patients: A Randomized, Blinded, Parallel‐group, Feasibility Trial. *JPEN J Parenter Enteral Nutr*. Published online 2020. doi:10.1002/jpen.2059

23. Nakamura K, Nakano H, Naraba H, et al. High protein versus medium protein delivery under equal total energy delivery in critical care: A randomized controlled trial. *Clin Nutr*. Published online 2020. doi:10.1016/j.clnu.2020.07.036

24. de Azevedo JRA, Lima HCM, Frota PHDB, et al. High-protein intake and early exercise in adult intensive care patients: a prospective, randomized controlled trial to evaluate the impact on functional outcomes. *BMC Anesthesiol*. 2021;21(1). doi:10.1186/s12871-021-01492-6

25. Carteron L, Samain E, Winiszewski H, et al. Semi‑elemental versus polymeric formula for enteral nutrition in brain‑injured critically ill patients:a randomized trial. *Crit Care*. 2021;25(31):1-12. doi:10.1186/s13054-020-03456-7

26. Dresen E, Weißbrich C, Fimmers R, Putensen C, Stehle P. Medical high-protein nutrition therapy and loss of muscle mass in adult ICU patients: a randomized controlled trial. *Clin Nutr*. Published online 2021. doi:10.1016/j.clnu.2021.02.021

27. Kagan I, Cohen J, Bendavid I, et al. Effect of Combined Protein-Enriched Enteral Nutrition and Early Cycle Ergometry in Mechanically Ventilated Critically Ill Patients—A Pilot Study. *Nutrients*. 2022;14(8). doi:10.3390/nu14081589

28. Heyland DK, Patel J, Compher C, et al. The effect of higher protein dosing in critically ill patients with high nutritional risk (EFFORT Protein): an international, multicentre, pragmatic, registry-based randomised trial. *Lancet*. 2023;401(10376):568. doi:10.1016/s0140-6736(22)02469-2

29. Zhu R, Allingstrup MJ, Perner A, Doig GS. The effect of IV amino acid supplementation on mortality in ICU patients may be dependent on kidney function: Post hoc subgroup analyses of a multicenter randomized trial. *Crit Care Med*. 2018;46(8):1293-1301. doi:10.1097/CCM.0000000000003221
